# Middle Stone Age Ochre Processing and Behavioural Complexity in the Horn of Africa: Evidence from Porc-Epic Cave, Dire Dawa, Ethiopia

**DOI:** 10.1371/journal.pone.0164793

**Published:** 2016-11-02

**Authors:** Daniela Eugenia Rosso, Africa Pitarch Martí, Francesco d’Errico

**Affiliations:** 1 UMR-CNRS 5199 de la Préhistoire à l'Actuel: Culture, Environnement et Anthropologie (PACEA), Université de Bordeaux, Pessac, France; 2 Seminari d'Estudis i Recerques Prehistòriques (SERP), Departament de Prehistòria, Història Antiga i Arqueologia, Universitat de Barcelona, Barcelona, Spain; 3 Grup de Recerca Aplicada al Patrimoni Cultural (GRAPAC), Departament de Biologia Animal, de Biologia Vegetal i d'Ecologia, Universitat Autònoma de Barcelona, Bellaterra, Spain; 4 Evolutionary Studies Institute and DST/NRF Centre of Excellence in Palaeosciences, and School of Geosciences, University of the Witwatersrand, Johannesburg, South Africa; Universidade do Algarve, PORTUGAL

## Abstract

Ochre is a common feature at Middle Stone Age (MSA) sites and has often been interpreted as a proxy for the origin of modern behaviour. However, few ochre processing tools, ochre containers, and ochre-stained artefacts from MSA contexts have been studied in detail within a theoretical framework aimed at inferring the technical steps involved in the acquisition, production and use of these artefacts. Here we analyse 21 ochre processing tools, i.e. upper and lower grindstones, and two ochre-stained artefacts from the MSA layers of Porc-Epic Cave, Dire Dawa, Ethiopia, dated to ca. 40 cal kyr BP. These tools, and a large proportion of the 4213 ochre fragments found at the site, were concentrated in an area devoted to ochre processing. Lower grindstones are made of a variety of raw materials, some of which are not locally available. Traces of use indicate that different techniques were employed to process ochre. Optical microscopy, XRD, μ-Raman spectroscopy, and SEM-EDS analyses of residues preserved on worn areas of artefacts show that different types of ferruginous rocks were processed in order to produce ochre powder of different coarseness and shades. A round stone bearing no traces of having been used to process ochre is half covered with residues as if it had been dipped in a liquid ochered medium to paint the object or to use it as a stamp to apply pigment to a soft material. We argue that the ochre reduction sequences identified at Porc-Epic Cave reflect a high degree of behavioural complexity, and represent ochre use, which was probably devoted to a variety of functions.

## Introduction

Evidence for systematic exploitation of ochre has been reported at several Middle Stone Age (MSA) sites from North and South Africa [1–9], as well as Mousterian and Châtelperronian sites in Europe [10–16] and the Middle East [17,18]. Here, we define "ochre" as rocks containing a high proportion of iron oxides, often mixed with silicates and other mineral substances, which are red or yellow in colour, or are streaked with such shades [19]. The use of these iron-rich minerals has often been interpreted to reflect high cognitive functions and symbolic thinking [1,3,9,20,21]. However, this view has been contested, as some evidence indicates that ochre may have also been used for functional purposes [19,22–29].

With the exception of a few sites in Sub-Saharan and North Africa, information on the way ochre was selected, processed, stored, and used is still scarce. This complicates the detection of behavioural similarities and what such behaviours may represent in terms of cognition and cultural complexity. Analysis of artefacts stained with ochre and involved, to varying degrees, in the treatment, storage and use of ochre have been conducted on Middle Stone Age and Mousterian knapped lithics (possibly stained by hafting or ochre processing) [27,28,30,31], shell containers [5,16], and personal ornaments [16,32–35]. Such analyses remain few in number due to the rarity of these objects in the archaeological record and the methodological challenges associated with the analysis of microscopic ochre residues.

In order to gain a better understanding of ochre processing and use in the East African MSA, and to evaluate the degree of behavioural complexity reflected by these activities, we present the first detailed analyses of ochre processing tools (OPT), namely upper and lower grindstones, and ochre-stained artefacts (OSA), consisting of stained pebbles and cobbles, recovered by Kenneth D. Williamson [36] in 1975 and 1976 from the MSA layers of Porc-Epic Cave (Dire Dawa, Ethiopia). The interest of these objects lies in their number, variety, excellent state of preservation of surface modifications, consistent presence of ochre residues, and the fact that they are associated with the most abundant collection of ochre pieces ever found at a Palaeolithic site [37]. In addition, research on the spatial distribution of pigment lumps and ochre processing tools has shown that concentrations of these artefacts are present at the site. The location of these concentrations shift through time, thereby offering the possibility of documenting temporally changing behavioural patterns. Thus, one has a unique opportunity to comprehensively reconstruct technical processes involved in the treatment and use of ochre in an area of the African continent virtually unexplored in this respect and for a key period for hominin cultural and biological evolution.

### Early ochre processing tools and ochre containers

Ochre processing tools, ochre containers, and ochre-stained artefacts from MSA and Middle Palaeolithic contexts are, in most cases, only briefly mentioned in the literature and have rarely been analysed in detail [5]. Additionally, they often present a poor state of preservation, with little or no trace of residue.

In Africa, the earliest tools that may have been used to process ochre are found in early MSA contexts. At the site of GnJh-15, in the Kapthurin formation, Kenya, possible grindstones stained with ochre were found in layers dated to 500–284 ka [8,38]. A quartzite cobble with ochre stains, interpreted as an ochre processing tool, was recovered at Twin Rivers, in Zambia [1,39]. At Sai 8-B-11, Sai Island, Sudan, sandstone mortars shaped by knapping and small chert pebbles with residues of red and yellow ochre are reported from levels dated to ca. 180 ka [40,41].

At Blombos Cave, South Africa, two toolkits used for the production and storage of ochre-rich compounds were recovered from layers dated to 100 ka [5]. These toolkits include two large abalone shells containing an ochre-rich compound composed of ochre powder and microflakes of two types of ferruginous siltstone (composed of quartz, hematite, muscovite/illite, and goethite), fragments of crushed trabecular bone, crushed compact bone, charcoal, and fragments of grindstones made of quartz, quartzite and silcrete. The two shells were found in close proximity to utilized ochre lumps, bones, as well as upper and lower grindstones. Two rhyolite grinders, and a faceted quartz mortar with ochre residues are reported from Ngalue Cave, in Mozambique, in levels dated between >42 ka and 105 ka. One of these shows the presence of possible starch residues [42,43]. At Klasies River, South Africa, a piece of tabular quartzite, battered on one edge, and bearing possible ochre residues, was found in shelter 1A in levels dated to 80–65 ka [44]. Two ochre-stained upper grindstones (one of which is quartzitic sandstone) and several ochre-stained artefacts are reported from middle–late MSA layers at Die Kelders, South Africa [45–47]. Nine backed tools with ochre on the cutting edges were found at Rose Cottage Cave in layers dated to 68–60 ka [31]. At Sibudu, South Africa, the presence of cemented hearths with ochre powder deposits was observed in layers dated to ca. 58 ka, suggesting that they were used as receptacles for ochre powder or as work surfaces on which grindstones were placed during the processing of ochre pieces [48]. Sandstone slabs, dolerite and hornfels tools with yellow or red residues were also recovered at the site [49]. Scrapers and flakes from late MSA layers with ochre residues on their working edges were interpreted as ochre processing tools [26,28,30]. Two diorite chunks and one diorite cobble with pigment residues that suggest grinding or scoring were found in MSA layers (dated to ca. 119–46 ka) at Yserfontein, South Africa [50]. Six broken pieces of quartzite with ochre residues, interpreted either as bearing paint or as "ochre-smeared slabs of non-artefactual stone" were found in MSA layers at Umhlatuzana Rock Shelter, South Africa [51]. At Bushman Rockshelter, South Africa, several broken grindstones, some with traces of ochre, were found both in MSA (ca. 47–43 ^14^C kyr BP) and LSA levels [52–55].

One sandstone fragment coated with red ochre was found in the MSA layers of Sehonghong, Lesotho [56]. Grindstones stained with ochre were also reported in Botswana, in the late MSA levels of ≠Gi [8]. In Mali, MSA levels of Songona I, dated to 55–35 ka, yielded sandstone artefacts with smoothed areas interpreted as possible ochre processing tools [57,58]. In Zimbabwe, granite slabs with ochre residues are found at Nswatugi and Pomongwe, in late MSA layers [8,59,60]. In East Africa, two flakes with traces of red ochre and one small ochre-stained lower grindstone were found at Enkapune Ya Muto, Kenya [61]. Grindstones have been recovered from other East African late MSA sites where the presence of ochre lumps is reported. However, it is not specified whether these tools bear ochre residues. Cases in point are Mochena Borago Rockshelter, in Ethiopia [62], and Mumba and Nasera rockshelters, in Tanzania [38,63].

In the Middle East, a possible ochre processing tool was found at Qafzeh cave, Israel, in layers dated to 100–90 ka. A centripetal recurrent mode Levallois core displays a concentration of ochre residues in the concavity of a large negative scar. This is interpreted as a core recycled into an ochre receptacle [18]. *Glycymeris* shells found in the same layers [64] have been interpreted as possible receptacles for ochre by some authors [35], or as ochered shell beads by others [65].

In Europe, grindstones found in early Mousterian levels (250–200 ka) at Beçov I, Czech Republic, were apparently used to process pigments [66,67]. In Germany, sandstone slabs with modifications attributed to the grinding of mineral material are reported in late middle Pleistocene levels of Rheindahlen [13,68]. At Cioarei-Boroşteni Cave, Romania, eight concave fragments of stalagmites and stalagmite crusts, showing ochre residues in concave areas, as well as scraping and polishing marks, were found in levels dated to ca. 52–45 ka BP are thought to be ochre containers [69–71]. In the same site was found an apparently painted geode, in levels dated to ca. 48 ka BP [70]. Grinding stones possibly used for mineral processing were also reported at Barakaevskaya Cave, in southern Russia [72,73].

In Spain, grindstones possibly used for ochre processing are reported from Mousterian levels at Cueva del Castillo and Cueva Morín [8,54], but the presence of ochre residues on these objects is not documented. At Cueva de los Aviones, Spain [16], in levels dated to ca. > 50 cal kyr BP, ochre residues were found on the inner side of an upper valve of a *Spondylus gaederopus* shell and have been interpreted as evidence for use of this shell as an ochre container. A use as ochre containers was also suggested for a *Callista chione* and two lower valve fragments of *Pecten maximus*. However, it has been argued that *S*. *gaederopus* upper valves have a limited volumetric capacity, which is insufficient for use for ochre processing and storing. A perforated *Glycymeris* shell with red residues identified as hematite was also found. In addition to these finds, an unmodified ancillary metatarsal of *Equus* sp. with orange residues on one extremity is reported from this site, suggesting that it functioned as a tool for the preparation or application of ochre [16].

At Pech-de-l'Azé I, France, a sandstone slab with black residues and diagnostic use-wear of grinding was found in Mousterian of Acheulean Tradition (MTA) levels, which are older than 43 cal kyr BP [15,74–76]. A limestone slab with pigment residues was also found in MTA levels at Le Moustier, France [12]. The absence of smoothed areas or homogenous pigment stains, suggested to the excavator that it was a painted rock rather than a grindstone. At Grotte de Néron, also in France, a limestone block interpreted as an ochre receptacle, possibly modified along its base by knapping and characterized by a central pit with ochre residues, was found in late Mousterian context [53,77]. The Châtelperronian levels of Grotte du Renne, France, have yielded an abundant collection of grindstones with red and black residues [14,53,78].

The earliest evidence of ochre processing tools in Sahul may date back to more than 50 kya. At Madjedbebe (Malakunanja II), in northern Australia, grindstones that sometimes show streaks of red residues were recovered in layers dated to ca. 55–45 ka [79]. At Nauwalabila I, also in northern Australia, levels dated to ca. 53 ka yielded a grindstone made of quartzite with flaked edges and abrasion marks on one face [80,81]. It shows no traces of pigment, but was stratigraphically associated with a large piece of worked hematite.

Ochre processing tools and ochre containers become ubiquitous at LSA and Upper Palaeolithic sites, including rock art contexts [53,82–84]. However relatively few artefacts and associated residues have been subjected to detailed analyses.

### Archaeological context

**Fig 1:**
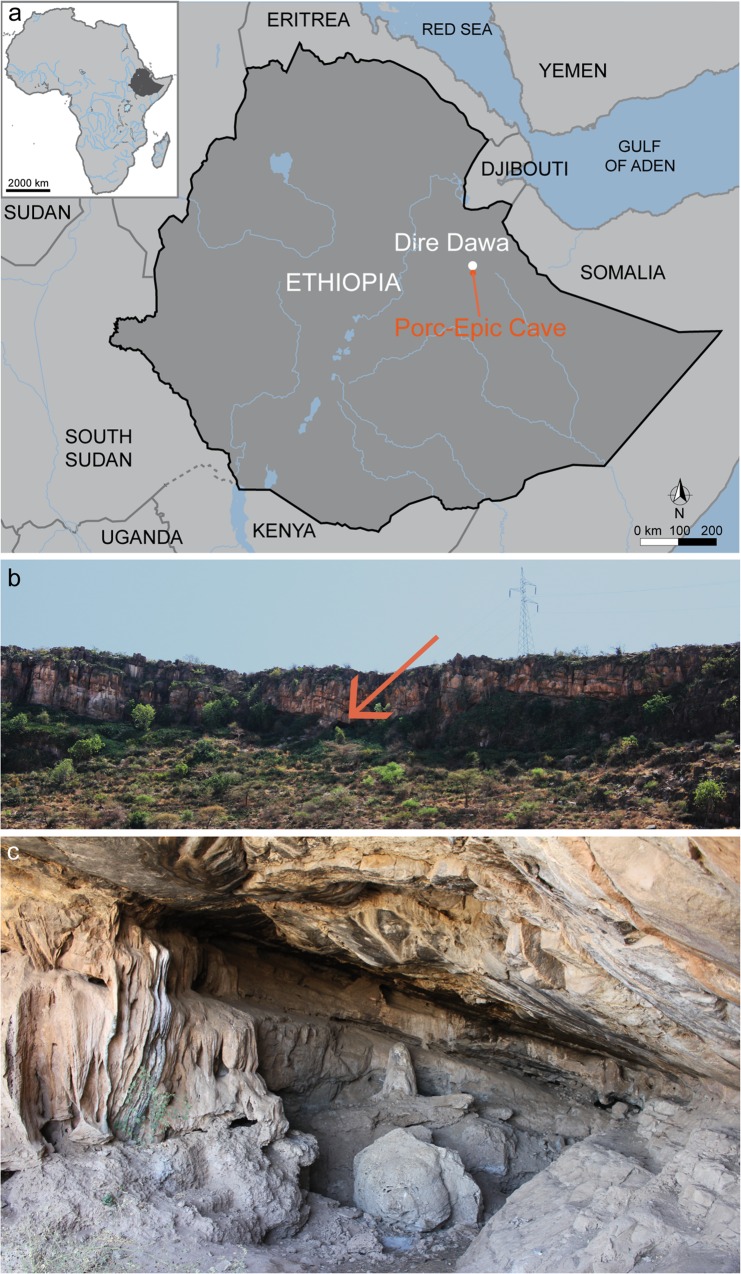
Location of Porc-Epic Cave. a: map of Ethiopia with location of the site; b: view of the cliff where the site is located. The arrow indicates the entrance of the cave; c: view of the cave from its entrance (photo A. Herrero).

Porc-Epic Cave is a key Palaeolithic site located between the Afar Depression and the Somali Plateau (Fig 1). It is situated 3 km south of Dire Dawa (Ethiopia), near the top of the Garad Erer hill, 140 m above the wadi Laga Dächatu, and opens at the base of a Jurassic limestone cliff. In 1929, Pierre Teilhard de Chardin and Henry de Monfreid discovered the site and carried out an initial survey that identified the presence of Palaeolithic levels [85] and rock art of a "later schematic style" [36,86]. The excavation was extended by Henri Breuil and Paul Wernert in 1933 [87] and by John Desmond Clark in 1974 [36,88]. In 1975–1976, the trench excavated by Desmond Clark was enlarged by Kenneth D. Williamson, covering an area of approximately 49 m^2^. In 1998, new data on Porc-Epic’s stratigraphic sequence were collected during fieldwork conducted by a team from the *Muséum National d’Histoire Naturelle*, Paris, and the Authority for Research and Conservation of Cultural Heritage (ARCCH) of Ethiopia [89].

**Fig 2:**
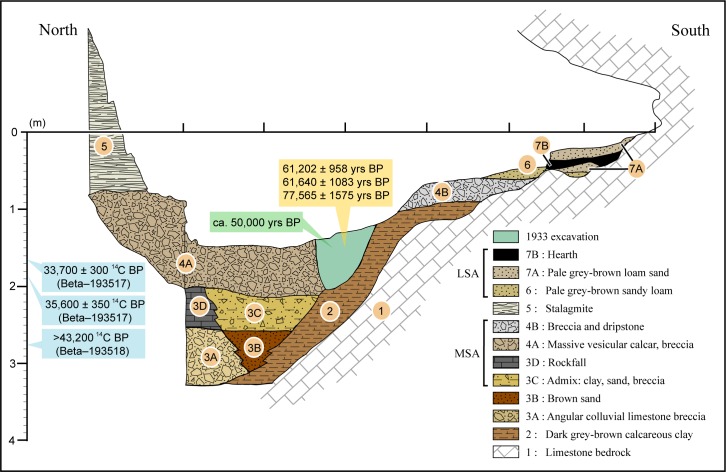
Porc-Epic Cave's stratigraphy. Eastern profile (09W-10W) at the end of the 1974 excavation. The gamma-spectrometry age of the human mandible and the obsidian hydration ages for artefacts recovered during the 1933 excavation are indicated in green and orange respectively. ^14^C ages obtained from gastropod opercula are indicated in blue. Their position within the stratigraphy is approximate, as only the depth and square at which these objects were found is known, and cannot be correlated to a specific layer. This figure is similar but not identical to the image from [37], and is therefore for illustrative purposes only.

The stratigraphy of the site (Fig 2) shows a succession of clayish, sandy levels and breccia, which were divided into seven stratigraphic units [36,37]. MSA artefacts were present between 60 and 220–230 cm below datum. According to Desmond Clark and Williamson [36], the earliest MSA artefacts were found in a layer of calcareous clay with angular rubble and a wedge of sand (level 2), and in layers of sand, clay and calcareous breccia with roof-fall deposits of limestone rubble (levels 3C and 3D). The main MSA assemblage was collected in levels consisting of calcareous breccia deposits (levels 4A and 4B) and sealed by the main dripstone. Post-dripstone activity and erosion removed deposits to a depth of approximately one meter at the entrance and towards the centre of the cave, where brown loam accumulated. LSA and Neolithic artefacts were found at the top of these poorly consolidated sediments, between 0 and 60 cm below datum. In these levels, consisting of fine sands and loam with interstratified hearth material, some post-depositional mixing may have occurred [90]. However, with the exception of the cave entrance, the main MSA assemblage was sealed and stratigraphically distinct [36].

Analysis of the lithic artefacts from the MSA levels of Porc-Epic Cave [36,89,91–96] revealed that the main raw materials exploited at the site were flint, basalt, obsidian [97,98] and sandstone/quartzite. Levallois, Discoid and Laminar reduction methods, employing direct hard-hammer percussion were used in the production of flakes, blades, bladelets and points. According to Desmond Clark and Williamson [36], the LSA levels can be clearly differentiated from the MSA levels due to the presence of microliths, small scrapers and *outils écaillés*. This, though, is not supported by Pleurdeau [89,94–96], who identifies the presence of a small number of microliths and backed bladelets in the MSA assemblage. According to this researcher, the presence of LSA features in levels attributed to the MSA may reflect a gradual evolution from the MSA to the LSA. However, recent studies [91] suggest that the presence of microliths may be the result of mixing with the overlying LSA layers, or of an intensive reduction of raw materials such as obsidian, which may occasionally produce micro-laminar blanks.

The analysis of the faunal remains showed the presence in the MSA levels of a variety of mammal taxa, suggesting the proximity of a water source and widespread grasslands. According to Assefa [90], the skeletal element profile suggests a selective transport of nutritional high-ranking elements to the site, which may have been a residential base during the MSA.

The MSA levels of the site also yielded a few human cranial fragments and a partial mandible, which exhibits a combination of modern and archaic features [99].

An accumulation of more than 400 gastropod opercula belonging to the terrestrial species *Revoilia guillainopsis* was found in the MSA levels. According to Assefa [100], this accumulation cannot result from natural processes and may be interpreted as evidence for symbolic behaviour, even though the analysis of the perforations shows no evidence of anthropogenic modifications.

A number of attempts have been made to radiometrically date the MSA layers (for a summary, see [37]). Three artefacts attributed to the MSA found during the 1933 excavation [101] were dated by obsidian hydration to 61,202 ± 958, 61,640 ±1083, and 77,565 ± 1575. However, this dating method is now considered unreliable [102,103]. The human mandible found in 1933 [99] provided a date of ca. 50 ka through high-resolution low-background gamma-ray spectrometry [91]. Accelerator mass spectrometry (AMS) radiocarbon determinations for three samples of gastropod opercula from the MSA layers [90] yielded ^14^C ages of 33,700 ± 300 (Beta–193517), 35,600 ± 350 (Beta–193516), and >43,200 (Beta–193518). The 95.4% probability range of the two finite ages are 38,800–37,049 cal BP, and 41,084–39,421 cal BP (IntCal13; OxCal 4.2; [104]).

### Ochre processing and use at Porc-Epic Cave

**Fig 3:**
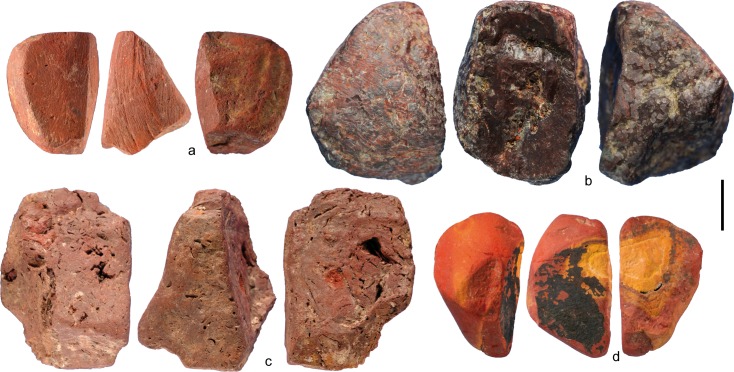
Ochre fragments from Porc-Epic Cave. a, b, d: fragments modified by grinding; c: fragment showing no modifications. Scale = 1 cm.

Ochre fragments and ochre processing tools were mentioned by Breuil et al. [105], and according to Desmond Clark and Williamson [36], 214 ochre fragments and "one sub-rectangular lower grindstone fragment of limestone showing one well-smoothed face and reddish alteration due to having been burnt" were recovered during the 1974 excavation. Analysis of the material from the 1975–1976 excavation has recently identified a hitherto unknown ochre assemblage comprising 4213 lumps (40 kg) of red, brown and yellow iron rich minerals (Fig 3) often modified by grinding (Fig 3A, 3B and 3D), as well as 23 possible ochre processing tools [37].

**Fig 4:**
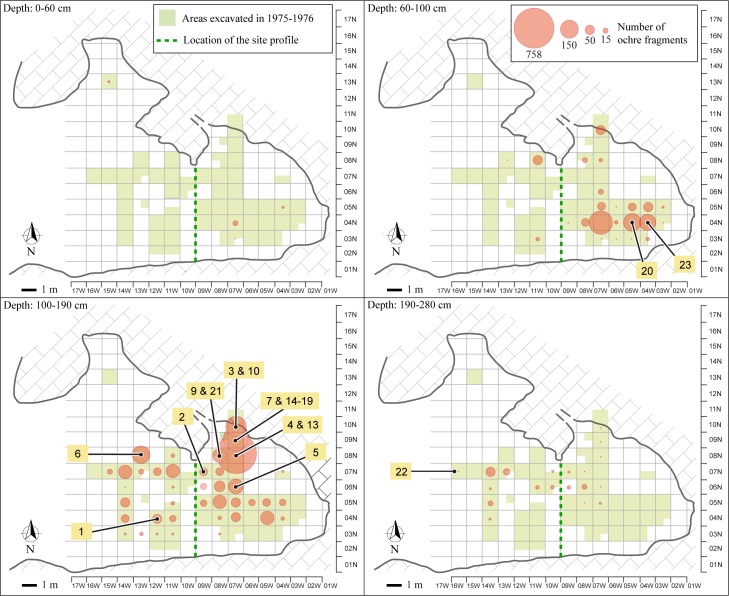
Spatial distribution of ochre fragments, ochre processing tools and ochre-stained artefacts. Bubble sizes reflect the frequency of ochre fragments per grid unit. Numbers refer to ochre processing tools and ochre-stained artefacts. The objects' identification number is the same as presented in Figs 5–11, Tables 1–5, S1 and S2 Figs, S1 Table, S1 Text. This figure is similar but not identical to the image from [37], and is therefore for illustrative purposes only.

The analysis of the spatial and stratigraphic distribution of these artefacts [37] clearly shows concomitant changes in the location of concentrations of ochre processing tools and ochre fragments (Fig 4). Between 60 and 100 cm below datum, an accumulation of ochre, accounting for 62,27% of the ochre pieces recovered within this depth interval, and two ochre processing tools, are observed in the southeastern area of the site (squares 04N-04W, 04N-05W and 04N-07W). Another concentration, accounting for 1373 ochre fragments (50,73% of the ochre fragments present at that depth interval), comes from a depth of 100 to 190 cm below datum, and is found in northeastern squares (squares 08N-07W, 08N-08W, 09N-07W, 10N-07W). Most of the ochre processing tools (n = 17) and two ochre-stained artefacts occur between 110 and 180 cm below datum. Among those tools, 76,4% (n = 13) were found in the same squares where the ochre fragment concentration occurs. The abundance of both ochre processing tools and ochre fragments in the same excavation units suggests that there were areas devoted to ochre processing, whose location shifted through time. The lower levels (190–280 cm below datum) yielded only one ochre processing tool at the northwestern area of the cave, towards the entrance.

Comparison of the distribution of ochre fragments and gastropod opercula dated by ^14^C suggests that the MSA deposits accumulated over a relatively short period of time and that the transport and use of ochre pieces at Porc-Epic most likely started around, or slightly before, 45 ka cal BP, and was particularly intense ca. 40 cal kyr BP [37]. However, the low number of ^14^C determinations and the fact that the dated samples come from three different areas of the site make it difficult to precisely establish for how long ochre was used.

## Materials and Methods

### Data collection, macroscopic and microscopic analysis

**Fig 5:**
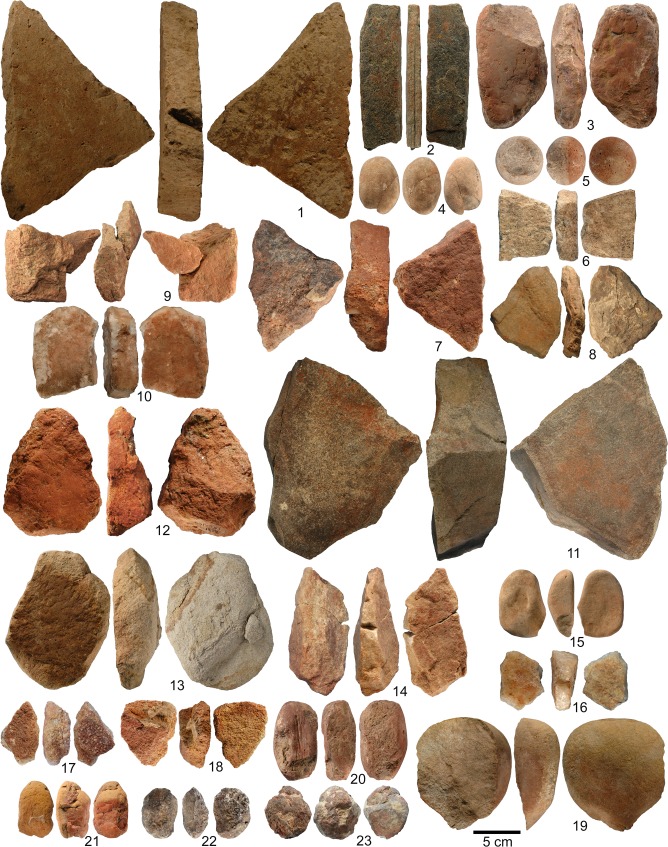
Ochre processing tools and ochre-stained artefacts found at Porc-Epic Cave. The objects' identification number is the same as presented in Figs 4 and 6–11, Tables 1–5, S1 and S2 Figs, S1 Table, S1 Text.

The material analysed in this study is curated at the National Museum of Ethiopia, in Addis Ababa. It includes twenty-three artefacts (OPT 1–4, 6–14, 16–23, OSA 5, 15; Fig 5 and S1 and S2 Figs) identified by one of us (DR) when examining the material from Williamson’s excavations [37]. Chipped stone tools bearing ochre residues were identified in the 1975–1976 material, but were excluded from this study. A permit to study the archaeological material and to export it temporarily was granted by the Authority for Research and Conservation of Cultural Heritages of Ethiopia (ARCCH). Material recovered during Teilhard de Chardin and de Monfreid’s survey (1929), Breuil and Wernert’s excavation (1933), and Desmond Clark’s excavation (1974) are not analysed in this paper.

Contextual, mineralogical, technological and morphometric variables were recorded for each object, and samples of residues were collected on a number of them (see below). In particular, we collected information on the spatial and stratigraphic provenance of the artefact, type of raw material, as well as object length, width, and thickness. The location and type of modifications, as well as the location of residue were also recorded.

Characterisation of the raw material was based on macroscopic and microscopic observation of the objects.

**Fig 6:**
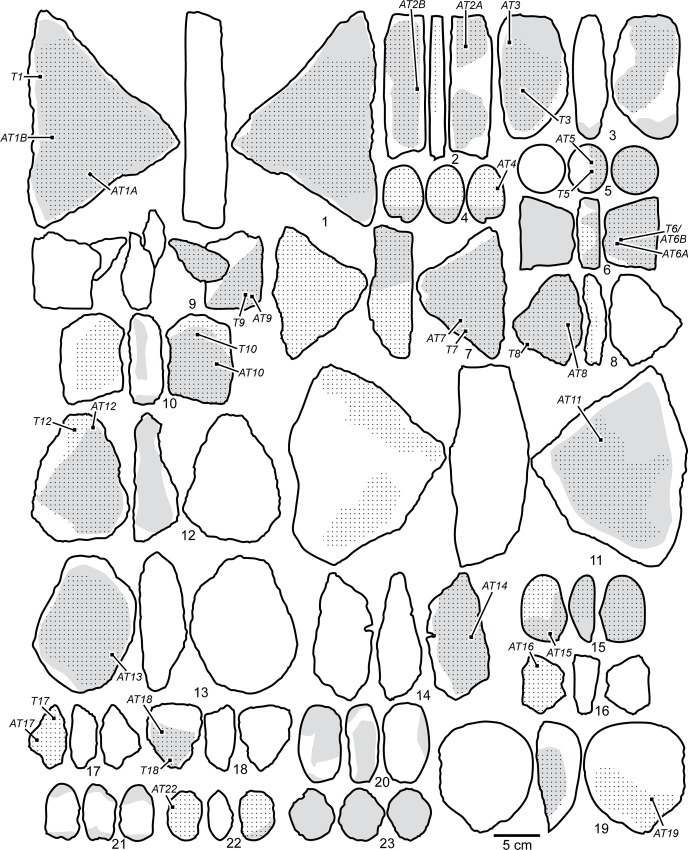
Drawings of the ochre processing tools and ochre-stained artefacts from Porc-Epic Cave. Ochre residues (dotted areas), traces of modification (gray areas), and location of the samples are indicated. The objects' identification number is the same as presented in Figs 4, 5 and 7–11, Tables 1–5, S1 and S2 Figs, S1 Table, S1 Text.

**Fig 7:**
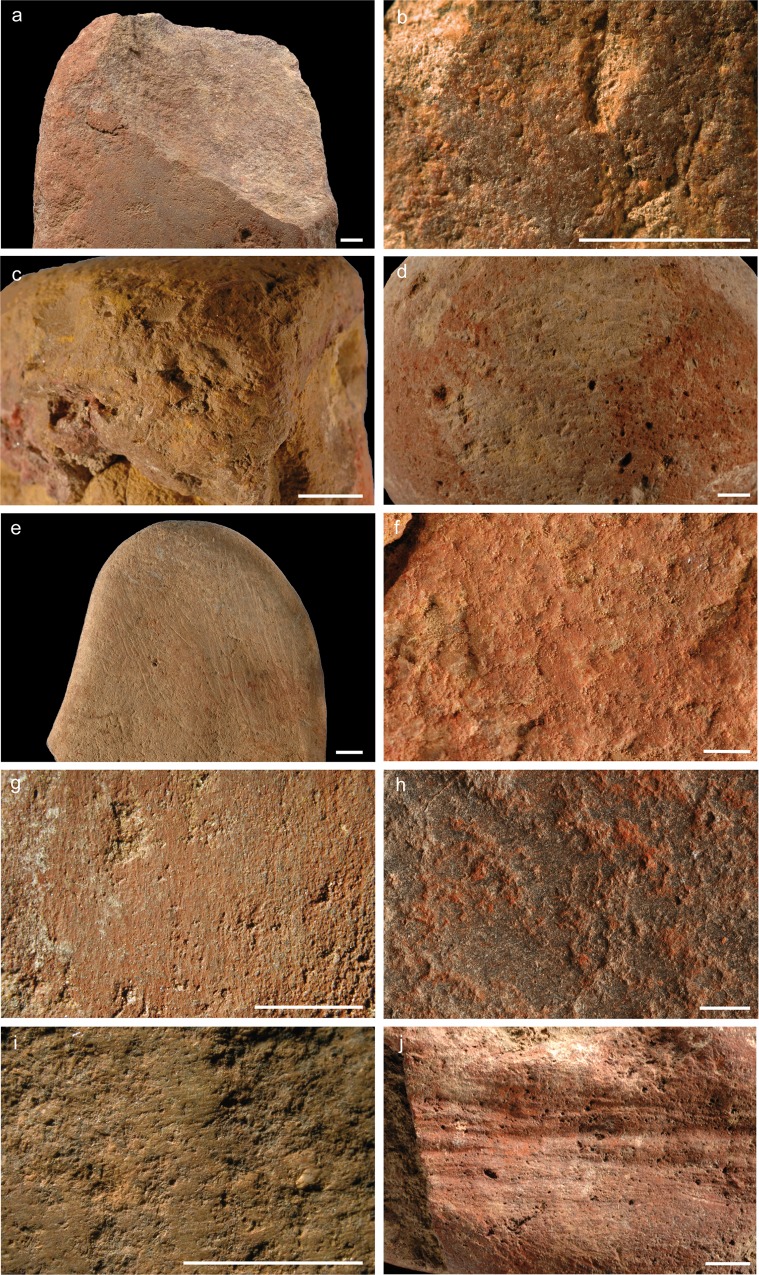
Modifications and use-wear on Porc-Epic Cave's ochre processing tools and ochre-stained artefacts. a: flake scar on OPT 3; b, c: pits on OPT 1 and 21; d: linear impressions on OSA 5; e: linear impressions and striations on OSA 15; f: smoothed areas on OPT 9; g-i: smoothed areas and microstriations on OPT 3, 11 and 13 respectively; j: deep grooves on OPT 20. Scales = 5 mm.

Anthropogenic (Fig 6) and natural modifications were also analysed and photographically documented using a Leica Z6 APO macroscope. We recorded the presence of flake scars (Fig 7A), pits (Fig 7B and 7C), linear impressions (Fig 7D and 7E), smoothed/levelled areas (Fig 7F*–*7I), microstriations (Fig 7G*–*7I), deep composite grooves (Fig 7J), and striations (Fig 7E). We define pits as depressions produced by a pounding action [82,106–108]. Linear impressions are elongated, irregular marks produced by a lithic edge impacting the stone surface during retouching [82]. Smoothing is the state of a surface that has lost, comparatively to neighbouring areas, irregularities and projections through abrasive action [82,106,109–111]. Smoothed areas may be covered in some cases by groups of superficial microstriations [107,109,112,113] not exceeding a width of 50 μm. Deep grooves are sub-parallel, slightly curved marks displaying on their walls multiple striae produced by the asperities of a lithic cutting edge vigorously scraping the object's surface [19,114]. Striations are produced by the punctual tangential contact of a sharp tool, such as a lithic cutting edge or a bone artefact, with a pebble surface [82].

### Ochre residue sampling and analysis

Residues from twenty artefacts were sampled with a scalpel under the microscope, on areas of approximately 2 mm^2^. The sampling (Fig 6) did not produce any visible damage to the objects. Sampling was easy to conduct on OPT 1–4, 6–8, 11, 16–19 and OSA 15 due to the abundance and softness of the residue, but was more difficult on OPT 9, 10, 12–14, 22 and OSA 5, due to the small amount and hardness of the identified residues.

**Fig 8:**
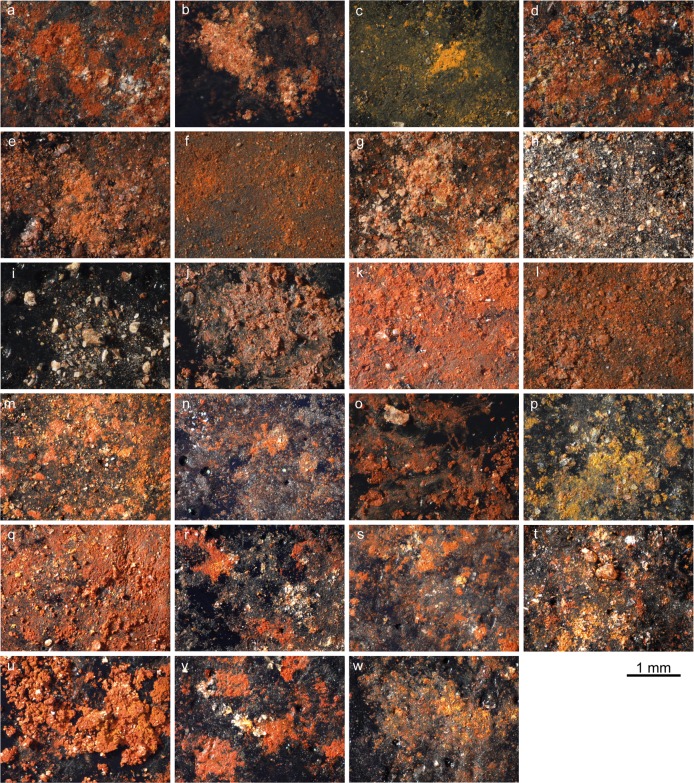
Ochre residues on carbon adhesive tabs. a: sample AT1A (OPT 1); b: sample AT1B (OPT 1); c: sample AT2A (OPT 2); d: sample AT2B (OPT 2); e: sample AT3 (OPT 3); f: sample AT4 (OPT 4); g: sample AT5 (OSA 5); h: sample AT6A (OPT 6); i: sample AT6B (OPT 6); j: sample AT7 (OPT 7); k: sample AT8 (OPT 8); l: sample AT9 (OPT 9); m: sample AT10 (OPT 10); n: sample AT11 (OPT 11); o: sample AT12 (OPT 12); p: sample AT13 (OPT 13); q: sample AT14 (OPT 14); r: sample AT15 (OSA 15); s: sample AT16 (OPT 16); t: sample AT17 (OPT 17); u: sample AT18 (OPT 18); v: sample AT19 (OPT 19); w: sample AT22 (OPT 22).

Sampled residues were collected following two protocols: (1) by applying carbon adhesive tabs to residues loosened by scalpel blades (samples AT1–19, AT22; Fig 8); or (2) by placing loosened residues in sample tubes (samples T1, T3, T5–10, T12, T17, T18). The second sampling method was carried out only when residues were abundant.

Residues were examined and photographed with a motorised Leica Z6 APOA macroscope equipped with a DFC420 digital camera. Images were treated with Leica Application Suite (LAS) equipped with a Multifocus module, and Leica Map DCM 3D computer software, which allowed for images with extended depths of field.

Micro-Raman spectroscopy (μ-RS), X-ray diffraction (XRD) and scanning electron microscopy coupled with energy dispersive X-ray spectroscopy (SEM-EDS) were used to characterise the composition of the residues (Table 1). The combined application of these three methods allows for the identification of the mineralogical and elemental composition of inorganic residues [5,16] such as those present on the studied objects. These techniques are complementary in that XRD provides an overall assessment of the minerals phases present in the sample, and μ-RS identifies the mineral composition of specific particles, which may in parallel be analysed with SEM-EDS for their elemental composition, morphology and distribution. Micro-RS was applied to all samples, XRD only when the quantity of residue was sufficient for obtaining reliable results, SEM-EDS when examination of the residue under optical microscopy indicated that the sample was not or less contaminated by sediment and/or tool fragments.

**Table 1:** Analyses conducted on ochre residue samples.

	SEM-EDS		μ-RS		XRD	
Num	Sample*	Num of an	Sample*	Num of an	Sample*	Num of an
1	AT1A	14	AT1A, B	5	T1	1
2	AT2A, B	14	AT2A, B	7	-	-
3	AT3	12	AT3	5	T3	1
4	AT4	8	AT4	6	-	-
5	AT5	11	AT5	4	T5	1
6	AT6B	9	AT6A	3	T6	1
7	AT7	13	AT7	12	T7	1
8	-	-	AT8	5	T8	1
9	AT9	15	AT9	8	T9	1
10	-	-	AT10	8	T10	1
11	-	-	AT11	9	-	-
12	AT12	6	AT12	5	T12	1
13	AT13	9	AT13	4	-	-
14	-	-	AT14	7	-	-
15	AT15	9	AT15	7	-	-
16	-	-	AT16	6	-	-
17	-	-	AT17	9	T17	1
18	-	-	AT18	7	T18	1
19	-	-	AT19	7	-	-
20	-	-	-	-	-	-
21	-	-	-	-	-	-
22	AT22	18	AT22	2	-	-
23	-	-	-	-	-	-

Num: number; An: analyses; SEM-EDS: Scanning electron microscopy coupled with energy dispersive X-ray spectroscopy; μ-RS: micro-Raman spectroscopy; XRD: X-ray diffraction. The objects' identification number is the same as presented in Figs 4–11, Tables 2–5, S1 and S2 Figs, S1 Table, S1 Text.

* Samples AT1–AT19, AT22 refer to residues stuck on carbon adhesive tabs, and samples T1, T3, T5–T10, T12, T17, T18 refer to loose residues kept in sample tubes.

Mineralogical analyses were carried out using a SENTERRA Dispersive Raman Microscope (Bruker), equipped with an internal calibration system. The working area was examined through the integrated colour camera. Spectra were acquired with a 785 nm laser, in a spectral range from 50 to 1500 cm^-1^, a laser power of 1 mW necessary to avoid thermal transformation of mineral phases, an integration time of 20 s, and with a number of co-additions ranging between 20 to 40 depending on the presence of fluorescence radiation and signal-to-noise ratio. Spectra were collected with the OPUS 7.2. software, and compared to those of the RRUFF spectra library [115] in order to identify mineral phases.

Mineralogical composition was also established by XRD using PANalytical X’pert MPD-PRO diffractometer (Bragg Brentano Theta-Theta geometry), with a copper anticathod (mean lambaKalpha = 15,418 Å). The working tension and intensity were set at 45 kV and 40 mA, respectively, and the time of analysis was of 19.30 h and 9.30 h, depending on the sample. Mineralogical phases were identified by using the routine DIFFRAC.SUITE™ EVA software package (Bruker AXS GmbH, Germany), combined with the specific powder diffraction file (PDF2) database (International Centre for Diffraction Data—ICDD, Pennsylvania, USA).

Elemental analyses were performed through SEM-EDS by using a FEI Quanta 200 with SiLi detector, and SDD-EDAX detector. The EDS analyses were performed at the same working distance (10 mm) and with the same acquisition time (100 s). Backscattered electron images (BSE) and elemental analyses were obtained under a low vacuum mode with an accelerating voltage of 10 kV and 15 kV.

## Results

### Artefact analysis

We identify artefacts displaying areas covered with ochre residues, associated with use-wear, as ochre processing tools (OPT). However, three ferruginous rock nodules on which no ochre residues could be identified were also included in this category. The shape and size of these objects, as well as the presence of use-wear (pits on the extremities or on the entire surface of the objects, and in one case smoothed areas) are consistent with a use for ochre processing. Among the ochre processing tools, we distinguish lower grindstones (i.e., flat or concave slabs used as passive tools to crush, pulverize, and grind iron-rich minerals) from upper grindstones (i.e., pebbles, cobbles or blocks used as active implements to facilitate the crushing or grinding of iron-rich minerals) [53,54,116–118]. One tool shows modifications indicative of a use as both an upper and lower grindstone. In addition, our collection includes a number of ochre-stained artefacts (OSA) that carry ambiguous or no use-wear traces.

#### Lower grindstones

**Fig 9:**
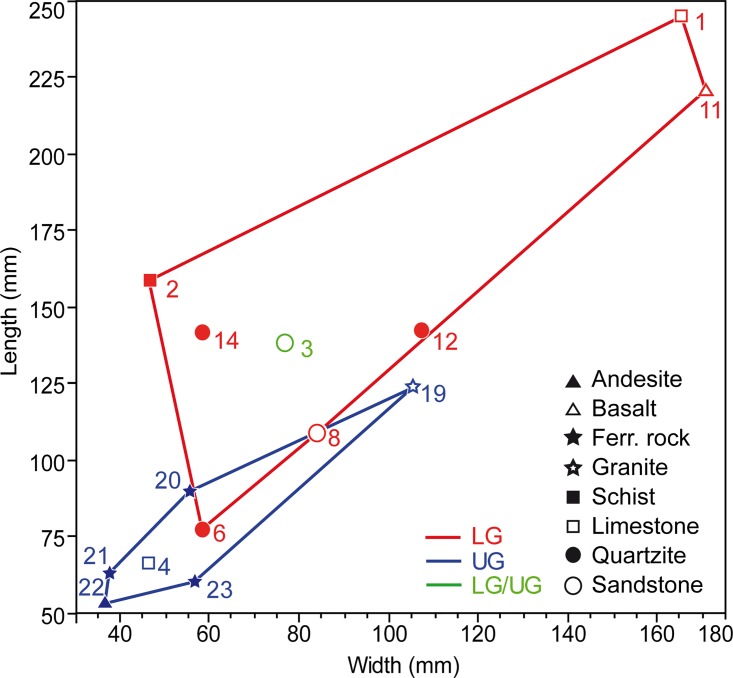
Biplot of length and width of Porc-Epic grindstones of different raw materials. LG: lower grindstone; UG: upper grindstone; ferr. rock: ferruginous rock. The objects' identification number is the same as presented in Figs 4–8, 10 and 11, Tables 1–5, S1 and S2 Figs, S1 Table, S1 Text.

Fourteen tools are interpreted as lower grindstones (Fig 5 no. 1, 2, 6–14, 16–18, Table 2, Figs A, B, F–K in S1 Fig, Figs A–C, E–G in S2 Fig). They are predominantly made of quartzite (n = 5) and sandstone (n = 3), with basalt, conglomerate, granite, granitoid, limestone and schist only used occasionally. Eight show recent fractures (Table 2). Undamaged lower grindstones are generally larger than upper grindstones (Fig 9), and two exceed 200 mm in length (OPT 1 and 11).

**Table 2:** Contextual and descriptive data of ochre processing tools and ochre-stained artefacts from Porc-Epic Cave.

Num	Square	Subsq	Depth	OCA	Preserv	Length*	Width*	Thick*	Rock type	Tool type	Supp inf
1	04N-12W	SE	130–140	-	C	245.0	165.0	46.4	Limestone	LG	Fig A in S1 Figs
2	07N-09W	NE	170–180	-	C	159	46.43	15.63	Schist	LG	Fig B in S1 Figs
3	10N-07W	SW	160–170	NEA	C	138.02	77.02	33.18	Sandstone	LG/UG	Fig C in S1 Figs
4	08N-07W	-	150–160	NEA	RF	65.83	46.51	42.39	Limestone	UG	Fig D in S1 Figs
5	06N-07W	NW	110–120	-	C	52.45	50.26	41.65	Limestone	OSA	Fig E in S1 Figs
6	08N-13W	-	150–160	-	OF	77.08	58.32	28.94	Quartzite	LG	Fig F in S1 Figs
7	09N-07W	SW	110–120	NEA	RF	(141,72)	(99,32)	(39,75)	Conglomerate	LG	Fig G in S1 Figs
8	-	NW	120–130	-	C	108.43	84.06	21.76	Sandstone	LG	Fig H in S1 Figs
9	08N-08W	NE	110–120	NEA	RF	(82,02)	-	(31,15)	Granite	LG	Fig I in S1 Figs
10	10N-07W	NE	120–130	NEA	RF	(100,67)	(80,07)	(26,9)	Quartzite	LG	Fig J in S1 Figs
11	-	-	-	-	C	220	170	56.73	Basalt	LG	Fig K in S1 Figs
12	-	-	150–160	-	C	142.19	107.52	53.45	Quartzite	LG	Fig A in S2 Figs
13	08N-07W	NW	150–160	NEA	RF	(152,56)	(109,09)	(43,16)	Granitoid**	LG	Fig B in S2 Figs
14	09N-07W	SE	120–130	NEA	RF	141.86	58.75	41.02	Quartzite	LG	Fig C in S2 Figs
15	09N-07W	SE	120–130	NEA	OF	71.49	50.12	28.06	Limestone	OSA	Fig D in S2 Figs
16	09N-07W	SE	120–130	NEA	RF	(66,99)	(49,63)	(29,4)	Quartzite	LG	Fig E in S2 Figs
17	09N-07W	SE	120–130	NEA	RF	(71,07)	(42,36)	(29,97)	Sandstone	LG	Fig F in S2 Figs
18	09N-07W	SE	120–130	NEA	RF	(72,36)	(62,47)	(28,4)	Sandstone	LG	Fig G in S2 Figs
19	09N-07W	SE	120–130	NEA	OF	123.34	105.74	46.81	Granite	UG	Fig H in S2 Figs
20	04N-05W	SW	70–80	SEA	C	88.84	55.75	39.39	Ferruginous rock	UG	Fig I in S2 Figs
21	08N-08W	SE	120–130	NEA	C	62.59	38.02	37.86	Ferruginous rock	UG	Fig J in S2 Figs
22	07N-16W	SW	210–220	-	C	52.14	37.06	27.02	Andesite	UG	Fig K in S2 Figs
23	04N-04W	SW	70–80	SEA	C	59.76	56.75	50.48	Ferruginous rock	UG	Fig L in S2 Figs

**Fig 10:**
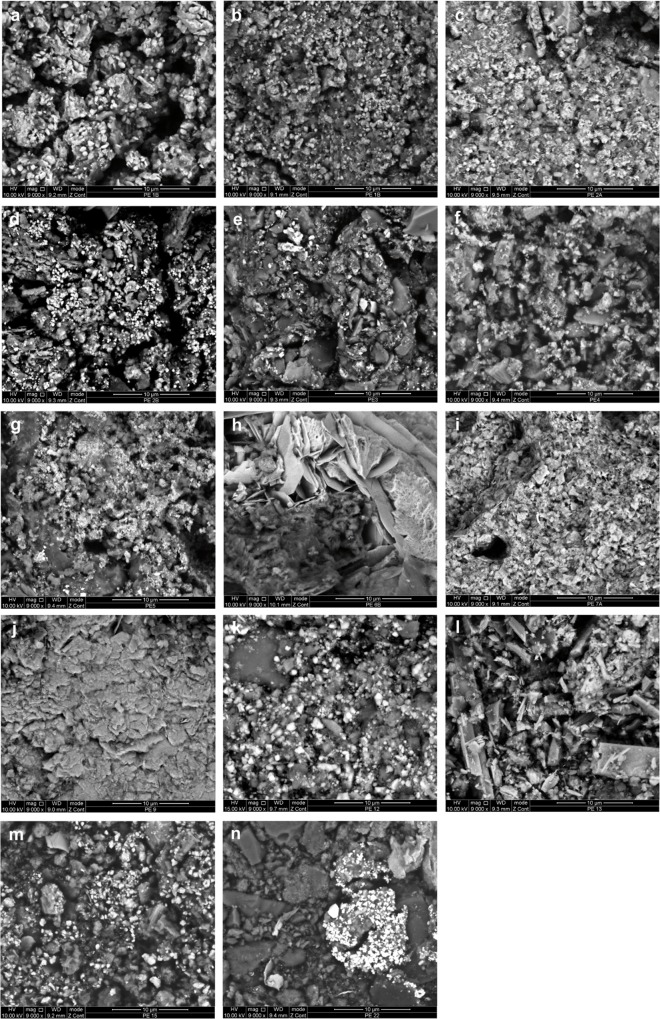
SEM images of ochre residues from Porc-Epic Cave's ochre processing tools and ochre-stained artefacts. All figures are in BSE mode. a: OPT 1, sample AT1 zone 1; b: OPT 1, sample AT1 zone 2; c: OPT 2, sample AT2A; d: OPT2, sample AT2B; e: OPT 3, sample AT3; f: OPT 4, sample AT4; g: OSA 5, sample AT5; h: OPT 6, sample AT6; i: OPT 7, sample AT7; j: OPT 9, sample AT9; k: OPT 12, sample AT 12; l: OPT 13, sample AT13; m: OSA 15, sample AT15; n: OPT 22, sample AT22. Scales = 10 μm.

**Fig 11:**
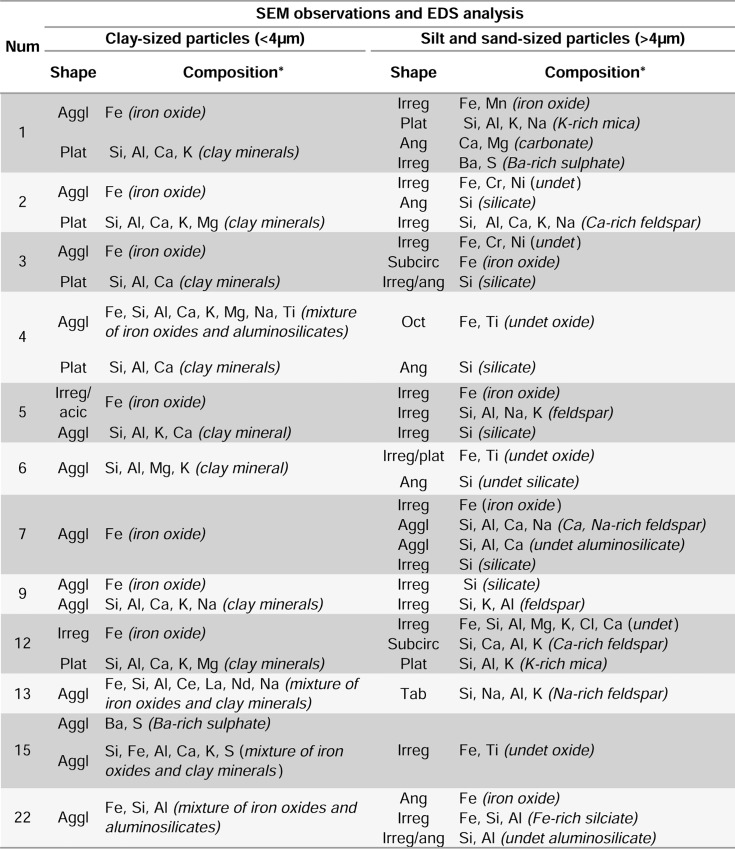
Results of SEM-EDS analyses on residues from Porc-Epic Cave's ochre processing tools and ochre-stained artefacts. Num: number; aggl: agglomerate; irreg: irregular; plat: platy; ang: angular; subcirc: subcircular; oct: octahedral; acic: acicular; tab: tabular. (*) Interpretation in brackets. The objects' identification number is the same as presented in Figs 4–10, Tables 1–5, S1 and S2 Figs, S1 Table, S1 Text.

Num: number; subsq: subsquare; OCA: ochre concentration area; NEA: northeastern area; SEA: southeastern area; preserv: preservation; C: complete; RF: recent fracture; OF: old fracture; thick: thickness; LG: lower grindstone; UG: upper grindstone; OSA: ochre-stained artefact; supp inf: supporting information. The objects' identification number is the same as presented in Figs 4–11, Tables 1 and 3–5, S1 and S2 Figs, S1 Table, S1 Text.

* In brackets, measurements on objects bearing recent fractures.

** Uncertain identification.

Lower grindstones display smoothed areas (Fig 7F, 7H and 7I, Table 3, Figs A, B, F–K in S1 Fig, Figs A–C and G in S2 Fig), identified on all pieces except OPT 16 and 17. On nine tools, smoothed areas are present on one flat surface (OPT 7–14, 18), on three, on both flat surfaces (OPT 1, 2, 6). In OPT 6, 7 and 12, smoothing is also observed on the lateral aspect. This is consistent with an abrasion, which requires large flat surfaces. On three lower grindstones (OPT 11, 13, 14), smoothed areas are covered by microstriations (Fig 7H and 7I, Table 3, Fig K in S1 Fig, Figs B, C in S2 Fig) oriented randomly (OPT 11, 14) or along the main axis of the artefact (OPT 13). Only on one artefact (OPT 1), the largest of the collection, smoothed areas also display pits (Fig 7B, Table 3, Fig A in S1 Fig), probably indicating a use for both abrading and pounding ochre lumps.

**Table 3:** Modifications and residues on Porc-Epic Cave's ochre processing tools and ochre-stained artefacts.

Num	FS	Pits	LI	Sm	Micr	DG	Str	Residue colour*	Residue loc
1	-	2F	-	2F	-	-	-	R + (Y)	2F
2	-	-	-	2F	-	-	-	R + Y	2F, L
3	2E	2E	-	2F	1F	-	-	R	2F
4	-	1E	-	-	-	-	-	R + (Y)	ES
5	-	-	1F	-	-	-	-	R + B	1F
6	1E, L	-	-	2F, L	-	-	-	R + (Y)	1F, L
7	-	-	-	1F, L	-	-	-	R + (Y)	ES
8	-	-	-	1F	-	-	-	R + (Y)	1F, L
9	-	-	-	1F	-	-	-	R + (B +Y)	1F
10	L	-	-	1F	-	-	-	R + (Y)	2F
11	-	-	-	1F	1F	-	-	R	2F
12	-	-	-	1F, L	-	-	-	R	1F
13	-	-	-	1F	1F	-	-	BRR + Y	1F
14	-	-	-	1F	1F	-	-	R + (Y)	1F
15	L	-	1F	-	-	-	2F, L	R + (Y)	ES
16	-	-	-	-	-	-	-	R + (Y)	1F
17	-	-	-	-	-	-	-	R + (Y)	1F
18	-	-	-	1F	-	-	-	R + (Y + O)	1F
19	-	L	-	-	-	-	-	R + (Y)	1F, L
20	2E	2E	-	1F, L	1F	1F	-	-	-
21	2E	2E	-	-	L	-	-	-	-
22	-	1E	-	-	-	-	-	R + (Y)	2F
23	-	ES	-	-	-	-	-	-	-

Num: number; FS: flake scars; LI: linear impressions; sm: smoothing; micr: microstriations; DG: deep grooves; str: striations; loc: location; 1E: one end; 2E: two ends; 1F: one face; 2F: two faces; L: lateral face(s); ES: entire surface; R: red; Y: yellow; B: brown; BRR: brownish red; O: orange. The objects' identification number is the same as presented in Figs 4–11, Tables 1, 2, 4 and 5, S1 and S2 Figs, S1 Table, S1 Text.

* In brackets, coloured residue visible under optical microscope.

Lower grindstones show residues ranging in colour from yellow to dark brownish red. In nine cases (OPT 6, 8, 9, 12–14, 16–18; Figs F, H, I in S1 Fig, Figs A–C, E–G in S2 Fig), residues are present on one flat face, on four (OPT 1, 2, 10, 11; Figs A, B, J, K in S1 Fig) they are detected on both flat faces (Fig 6, Table 3). Three also show residues laterally (OPT 2, 6, 8; Fig 6, Table 3, Figs B, F, H in S1 Fig). OPT 7 (Fig 6, Table 3, Fig G in S1 Fig) is the only artefact that shows ochre residues across the entire surface. Residues are systematically found on smoothed areas, but also on areas with no use-wear (Fig 6).

In OPT 13 (Fig B in S2 Fig) and 10 (Fig J in S1 Fig), residues homogenously cover one (OPT 13) or two (OPT 10) larger surfaces. Two layers of residue are identified on OPT 13: a thin brown-red layer overlying a yellow layer, visible in areas in which the former is eroded, and along its perimeter. This is likely due to the successive treatment of two ochre types. It cannot be attributed to heating of the residue, leading to a transformation of goethite into hematite, since no traces of such an event (rubefaction, cracks, etc) are detected on the tool.

In two cases (OPT 2 and 7; Figs B, G in S1 Fig), patchy residues of different colour or texture are associated on the same face. On OPT 2, a few spots of yellow residue are observed in areas covered by red residue. On OPT 7, patchy ochre residues coexist with small accumulations of homogenous residue.

OPT 11 (Fig K in S1 Fig) represents a special case. A dark and defined ring-like deposit runs along the edge of both faces, reflecting a "coffee-ring effect" [119]. During the evaporation of a liquid substance, suspended particles are pushed to the edges of the area covered by the mixture, creating a ring-like deposit [120]. Residues of a different colour, resulting from a more recent use of the same surface, are also present in the centre of this artefact's face.

Continuous flake scars on the edges of two lower grindstones (OPT 6 and 10; Figs F, J in S1 Fig) suggest a use of these tools as hammerstones after their use for ochre treatment, since no traces of ochre are found on the flake scars.

#### Upper grindstones

The six tools interpreted as upper grindstones are made of limestone, andesite, granite and ferruginous rocks (Fig 5 no. 4, 19–23, Table 2, Fig D in S1 Fig, Figs H–L in S2 Fig). They are often smaller than lower grindstones (Fig 9).

Pits produced by pounding (Fig 7C, Table 3) are generally present on one or both ends (OPT 4, 20–22; Fig D in S1 Fig, Figs I–K in S2 Fig), and in two cases on a margin (OPT 19; Fig H in S2 Fig) and across the entire surface (OPT 23; Fig L in S2 Fig). They are in some cases associated with tangential small flake scars resulting from fractures produced during pounding (OPT 20, 21; Table 3, Figs I, J in S2 Fig). OPT 19 (Fig H in S2 Fig) is a fragment of a tool that accidentally split during use.

With the only exception of one object (OPT 19; Fig H in S2 Fig), which has only one area homogeneously covered with ochre, ochre residues appear on these tools in the form of individual spots, often present on the pitted areas (Fig 6). On the tools made of ferruginous rocks, they cannot be differentiated from the tools’ raw material (Figs I, J, L in S2 Fig). Deep grooves produced by a longitudinal scraping (Fig 7J, Table 3) and flat facets produced by abrasion are also present on one upper grindstone made of ferruginous rock (OPT 20; Fig I in S2 Fig). The facets may be due to extraction of pigment powder by rubbing the object against a lower grindstone or by using it to finely powder more friable iron-rich rocks. Striations due to abrasion (Table 3) are also visible on another grindstone made of ferruginous rock (OPT 21; Fig J in S2 Fig).

#### Upper and lower grindstone

An elongated sandstone cobble (OPT 3) displays modifications indicating use as both a lower and upper grindstone (Fig 5 no. 3; Table 2; Fig C in S1 Fig). Smoothed areas covered by longitudinally oriented microstriations (Fig 7G), with respect to the main axis of the artefact, are detected on both faces (Table 3). They are homogeneously covered with ochre. Pits are identified at both ends, along with a large flake scar created by the use of the tool in a pounding action posterior to its use as a lower grindstone (Fig 7A).

#### Ochre-stained artefacts

Two limestone artefacts (OSA 5, 15) display ochre residues with traces of modification unrelated to ochre treatment (Fig 5 no. 5 and 15, Table 2, Fig E in S1 Fig, Fig D in S2 Fig).

OSA 15 (Fig 5 no. 15, Fig D in S2 Fig) is a flat cobble homogeneously stained with ochre, which displays linear impressions produced by its use as a retoucher and groups of subparallel individual thin striations, resulting from scraping, or use of the cobble to retouch a lithic edge (Fig 7E, Table 3). In this artefact, the homogeneity of the residues associated with linear impacts and striations, suggests an unintentional staining during its use as a retoucher. This may have been produced either by it being used by a person covered in ochre, or with ochre-stained hands, or by close contact with ochre powder or ochre fragments, for example by carrying them in the same container. This is consistent with the analysis of ochre residues (see below), which suggests the presence of two different ochre types.

OSA 5 (Fig 5 no. 5; Fig E in S1 Fig) is a subspherical pebble. Half of its surface is homogeneously covered by an ochre stain, which is removed at one spot by linear impressions due to its use as a retoucher (Fig 7D; Table 3). The location and appearance of the stain suggests that the artefact was dipped in a liquid ochered medium, which was absorbed by the limestone. The pattern is consistent with a single production or use. Multiple uses would have probably left pigment residue of different shades and traces of pigment on the half of the surface that is not covered in ochre.

#### Rock type and spatial distribution

The depth (110–130 cm below datum) and area within the cave (northeastern zone [37]) where most of the processing tools are found (Fig 4, Table 2), also features the highest variety of rock types used (conglomerate, granite, quartzite, limestone, sandstone, and ferruginous rock). Two ochre processing tools (Fig 4, Table 2) made of andesite and schist (OPT 22 and 2) were found in lower levels (210–220 cm and 170–180 cm below datum respectively). No spatial information is available for the only object made of basalt (OPT 11; Table 2).

### Residue analysis

Under optical microscopy most residues from the ochre processing tools and ochre-stained artefacts appear as agglomerates of fine-grained powder (Fig 8). These agglomerates are particularly compact in the case of residues from OPT 9, 12 and 18 (Fig 8L, 8O and 8U, Fig I in S1 Fig, Figs A, G in S2 Fig). Residues sampled on OPT 6 (Fig 8H and 8I, Fig F in S1 Fig) are instead composed of isolated coarse grains, and those from OPT 7 (Fig 8J, Fig G in S1 Fig) take the form of small homogenous accumulations.

Even though macroscopically only two processing tools (OPT 2, 13; Table 3, Fig B in S1 Fig, Fig B in S2 Fig) show the presence of yellow residues, microscopically, samples display in most cases a majority of fine red grains associated with few yellow grains (Fig 8; Table 3). Four samples (from OPT 3, 11, 12 and OSA 5; Fig 8E, 8G, 8N and 8O, Figs C, E, K in S1 Fig, Fig A in S2 Fig) only feature red grains and two others (one of the samples from OPT 2 –AT2A– and the sample from OPT 13; Fig 8C and 8P, Fig B in S1 Fig, Fig B in S2 Fig) only contain yellow grains with rare red grains. All residues also contain translucent, white and black coarse particles in variable proportions (Fig 8).

Elemental (Figs 10 and 11, Figs A–G, I in S1 Fig, Figs A, B, D, K in S2 Fig, Table A in S1 Table, Texts A–G, I, L, M, O, T in S1 Text) and mineralogical (Tables 4 and 5; Figs A, C, E–J in S1 Fig, Figs A, F, G in S2 Fig; Table B in S1 Table; S1 Text) analyses indicate that the residues are composed of numerous minerals, including several types of oxides, aluminosilicates (clays, micas, and feldspars), silicates, carbonates, sulphates, and phosphates.

**Table 4:** Results of μ-RS analyses on residues from Porc-Epic Cave's ochre processing tools and ochre-stained artefacts.

Num	Sample	ab	an	aug	cal	dol	gth	gp	hem	kln	lep	mag	man	mnt	ms	qz	un. alum.	un. Mn ox.	C
1	AT1A,B						•		•						•		•		
2	AT2A,B						•		•						•	•	•	•	
3	AT3								•							•			
4	AT4						•	•	•							•	•		
5	AT5				•				•							•			
6	AT6A						•		•										
7	AT7						•		•		•					•			•
8	AT8						•	•	•							•			
9	AT9						•	•	•							•			
10	AT10				•		•		•				•			•	•		
11	AT11		•					•	•			•				•	•		
12	AT12	•							•							•	•		
13	AT13	•					•		•							•			•
14	AT14				•		•		•					•		•	•		•
15	AT15					•	•		•					•			•		
16	AT16						•	•	•							•			
17	AT17						•	•	•	•						•			
18	AT18						•	•	•			•				•			
19	AT19			•			•		•							•			
22	AT22						•		•					•		•			

Num: number; un: undetermined; alum: aluminosilicate; ox: oxide; C: carbon; abbreviations of minerals are based on the nomenclature suggested by [121], except for lepidocrocite (lep), and manganite (man). The objects' identification number is the same as presented in Figs 4–11, Tables 1–3 and 5, S1 and S2 Figs, S1 Table, S1 Text.

**Table 5:** Results of XRD analyses on residues from Porc-Epic Cave's ochre processing tools and ochre-stained artefacts.

Num	Sample	ab	an	ant	aug	ber	cal	clc	gth	gp	hal	hem	kln	lmt	mc	mgh	mnt	or	qz	ram	sa	sap
1	T1		•				•					•	•						•			
3	T3					•						•	•						•			
5	T5									•		•		•			•		•	•	•	
6	T6	•	•		•			•			•	•							•			•
7	T7											•							•			
8	T8			•								•	•			•			•			
9	T9	•	?									•			?		•		•			
10	T10						•					•		•					•		•	
12	T12								•			•							•			
17	T17						•					•	•					?	•			
18	T18											•	•						•			

Num: number; abbreviations of minerals are based on the nomenclature suggested by [121], except for bernalite (ber), halloysite (hal) and ramsdellite (ram). The objects' identification number is the same as presented in Figs 4–11, Tables 1–4, S1 and S2 Figs, S1 Table, S1 Text.

SEM-EDS and mineralogical analysis (Figs 10 and 11, Tables 4 and 5, Figs A–G, I in S1 Fig, Figs A, B, D, K in S2 Fig, S1 Table, S1 Text) of the residues from twelve ochre processing tools reveals that six (OPT 3, 6, 7, 9, 12, 13; Fig 10E, 10H, 10I, 10J, 10K and 10L, Figs C, F, G, I in S1 Fig, Figs A, B in S2 Fig) include element associations and morphological features indicating the presence of a single type of ochre per tool; five (OPT 1, 2, 4 and OSA 5, 15; Fig 10A, 10B, 10C, 10D, 10F, 10G and 10M, Figs A, B, D, E in S1 Fig, Fig D in S2 Fig) display associations suggesting the presence of two types of ochre. A single tool (OPT 22; Fig 10N and Fig 11, Table 4, Fig K in S2 Fig, S1 Table, Text T in S1 Text), made of an iron-rich rock, shows residues that come either from surface alteration or from use. Components interpreted as contamination from the tool, surrounding sediment and post-depositional processes are discussed in more detail in S1 Text.

Among those from the first group, samples from OPT 7 and 9 (Fig 10I, 10J and Fig 11, Tables 4 and 5, Fig G, I in S1 Fig, S1 Table, Texts G, I in S1 Text) are very similar and almost exclusively composed of a pure earthy hematite characterised by agglomerates of iron oxide platelets. Goethite is also found on both samples. The only difference lies in the size of the platelets, smaller in OPT 7 (2–4 μm) than in OPT 9 (5–10 μm), and the presence of lepidocrocite in the sample from OPT 7. Ca and Na-rich feldspars, quartz grains, iron oxide particles in OPT 7, and plagioclase feldspars (albite, anorthite), alkali feldspars (probably microcline), quartz grains, clays from the smectite group (probably montmorillonite), iron oxide particles, and gypsum in OPT 9 probably derive from the grinding tool and the surrounding sediment.

The sample from OPT 3 (Fig 10E and Fig 11, Tables 4 and 5, Fig C in S1 Fig, S1 Table, Text C in S1 Text) contains exogenous iron-rich submicrometric particles identified as hematite associated with clay minerals from the kaolinite-serpentine group (Si, Al, Ca, K, Mg and traces of Ti) and quartz grains–cemented by iron oxides–, which probably derive from the grinding tool. Fragments of quartz grains with clean edges (5–10 μm) may be interpreted as an additive or resulting from fragmentation of grains originating from the tool.

The residue from OPT 12 (Fig 10K and Fig 11, Tables 4 and 5, Fig A in S2 Fig, S1 Table, Text L in S1 Text) features subcircular iron-rich particles, 1–2 μm in width, with Mn traces, identified as hematite and goethite, consistently associated with micrometric platelets of clay minerals (Si, Al, K, Ca, Mg), a few K-rich platelets interpreted as micas (Si, Al, K, Na), and Ca-rich feldspar particles (possibly albite). Quartz grains certainly derived from the OPT were also detected.

The sample from OPT 13 (Fig 10L and Fig 11, Table 4, Fig B in S2 Fig, S1 Table, Text M in S1 Text) contains agglomerates of submicrometric acicular and irregular iron oxide particles with traces of Mn, identified as goethite and hematite, associated with clays (Si, Al, Na, with low contents of K, Ca, Mg, and traces of Ti). Large tabular Na-rich feldspars (possibly albite), quartz grains, and rare earth elements (La, Ce, Nd) are interpreted as originating from the tool stone.

Ochre residues from OPT 6 (Fig 10H and Fig 11, Tables 4 and 5, Fig F in S1 Fig, S1 Table, Text F in S1 Text) include grains of 55–134 μm in length containing thin platelets composed of Fe and Ti. Hematite and goethite were identified by mineralogical analysis. Agglomerates of submicrometric grains interpreted as clay minerals (Si, Al, Mg, K), possibly saponite and halloysite, coat, in places, the large oxide grains. Clay minerals do not appear to originate from the iron rich ground rock, nor from the processing tool, which make of them good candidates for being additives. Angular, coarse grains (Si, Ca, Mg, Fe, Al, Ti, with traces of Na; approximately 194 μm in length) identified as mineral phases from chlorite and pyroxene groups (probably clinochlore and augite) were also detected in the sample. These grains, commonly found in metamorphic rocks, as well as quartz and plagioclase feldspars (such as albite and anorthite), probably originate from the processing tool.

Among the samples containing two ochre types, that from OSA 5 (Fig 10G and Fig 11, Tables 4 and 5, Fig E in S1 Fig, S1 Table, Text E in S1 Text) is composed of individual, large (17–38 μm in length), and numerous small (1–2 μm in length) iron-rich particles, identified as hematite. The latter, which occasionally present an acicular morphology, are embedded in a clay matrix (Si, Al, K, Ca, with traces of Mg, Na and Ti) identified as montmorillonite. Calcite and laumontite originate from the tool. Gypsum may have grown post-depositionally. Manganese oxides (ramsdellite), alkali feldspars (possibly sanidine) and quartz grains could either come from the tool or could be part of the ochre residue.

OPT 4 (Fig 10F and Fig 11, Table 4, Fig D in S1 Fig, S1 Table, Text D in S1 Text) includes octahedral undetermined Fe-Ti oxides with a length of 58–63 μm, and agglomerates of submicrometric grains made of hematite and goethite that are probably naturally associated with clay minerals (Si, Al, Ca, and traces of K, Mg, Na). Quartz grains are interpreted as part of the ochre residue since such grains are rare in the rock composing this tool. They could come from the processed ochre fragments or represent an additive intentionally mixed to the ochre powder. The presence of gypsum is likely due to post-depositional processes.

The sample from OPT 1 (Fig 10A, 10B and Fig 11, Tables 4 and 5, Fig A in S1 Fig, S1 Table, Text A in S1 Text) is composed of two different mineral associations. The first shows aggregates of submicrometric iron oxides (Fe) identified as hematite and goethite mixed with clay minerals (Si, Al, Ca, K, Mg, and traces of Ti and Na) from the kaolinite group, as well as micas consisting of muscovite (Si, Al, K, Na). The second is more compact, features submicrometric and large aggregates (approximately 39 μm in length) of iron oxides (Fe, Mn), plagioclase feldspars (probably anorthite), absent in the former, and does not contain titanium in the clay minerals. Calcite, and quartz are probably part of the rock composing the tool. Detected barium sulphates and carbonates could derive from surrounding sediment or post-depositional processes.

A different type of iron oxide is detected on each of the two samples collected on OPT 2 (Fig 10C, 10D and Fig 11, Table 4, Fig B in S1 Fig, S1 Table, Text B in S1 Text). Yellow in colour, sample AT2A (Fig 10C) contains compact agglomerates of submicrometric acicular grains made of goethite and, occasionally hematite, with traces of Ti mixed with platy particles of clay minerals (Si, Al, Ca, K, with traces of Mg, Ti, Na). Red in colour, the second sample (AT2B, Fig 10D) is composed of submicrometric grains of hematite (Fe, with traces of Ti) associated with platy grains of clay minerals (Si, Al, Ca, K, and traces of Mg, Na), taking the form of less compact agglomerates. Angular silicate grains (approximately 42–156 μm in length) identified as quartz, and undetermined Mn-rich minerals detected in both residues probably come from the OPT. This is also the case for irregular Ca-rich grains corresponding to plagioclase feldspars (Si, Al, Ca, K, Na; approximately 62 μm in length), and K-rich micas (muscovite), observed in AT2A.

Of the two ochre components identified in the sample from OSA 15 (Fig 10M and Fig 11, Table 4, Fig D in S2 Fig, S1 Table, Text O in S1 Text), the first includes submicrometric grains of hematite and goethite (Fe with traces of Ti) associated with clay minerals (Si, Al, Ca, K, with traces of Na, Mg) from the smectite group (probably montmorillonite). The consistent combination of iron oxides and clay minerals suggests that they come from the same rock. The second type of ochre displays large particles of Fe oxides richer in Ti, with traces of Mn (approximately 20–79 μm in length). Fine-grained agglomerates composed of submicrometric to micrometric Ba-rich sulphates (Ba, S) and dolomite were also identified, but probably originate from the tool.

In summary, SEM-EDS and mineralogical analyses identify three main ochre groups:

- (1) ferruginous clay rocks (OPT 1–4, 12, 13, and OSA 5 and 15);
- (2) pure earthy hematite with a low content of goethite (OPT 7, 9);
- (3) isolated coarse iron oxide particles (OSA 5) sometimes containing titanium (OPT 4, 6, and OSA 15).

Differences between groups 1 and 2 are most likely due to the fact that two types of rock were processed. Group 3 is more difficult to interpret, as it may represent either an extreme in variation of ferruginous clay rocks or a different rock type.

In addition, these three categories may contain quartz, feldspars and other silicates, clays, calcite, and calcium or barium sulphates. They may originate from the processed ochre, the grindstone, loading agents, surrounding sediments, or be the result of post-depositional processes.

XRD (Table 5) and μ-RS analysis (Table 4, Table B in S1 Table) of samples not studied with SEM-EDS identified the same minerals found on the samples also studied with SEM-EDS (Texts H, J, K, N, P–S in S1 Text).

### Processing tools and processed ochre

No obvious relationship is observed between the type of processed ochre and the raw material from which the ochre processing tools are made (Table 2). Ferruginous clay powder was detected on limestone, schist, sandstone, granitoid and quartzite tools. Isolated iron oxide particles were found on limestone and quartzite tools. Earthy hematite was identified on conglomerate and granite tools. Cases in point are the ochre type identified on OPT 1 and OPT 12 (Fe and Mn oxides, clay minerals, K-rich micas and Ca-rich feldspars), and samples from OPT 7 and 9 (earthy hematite). Ochre types identified on OSA 15 also show similarities with ochre residues from one of the samples from OPT 2 (AT2B, submicrometric iron oxides with traces of Ti, associated with clay minerals), and the sample from OPT 6 (Fe-Ti oxides).

## Discussion

Results of the analysis of ochre-stained artefacts and ochre processing tools from Porc-Epic Cave provide a means of identifying technical preferences, evaluating the behavioural complexity behind these activities and understanding, to some extent, their goals. The appearance, granulometry and colour of ground ochre powder depends on the grinding/pounding technique, the nature and state of freshness of the grinding tools, the composition, texture and hardness of the processed iron-rich rock, the pressure applied, the duration of the grinding/pounding actions, and the eventual presence of loading or binding agents [6,14,19].

Porc-Epic’s MSA inhabitants used a variety of rock types as grindstones. The time and effort necessary to locate and transport these rocks to the site is difficult to evaluate since the only available information comes from geological maps [122], and studies on the provenance of rocks used for knapped lithics [94]. We know that limestone, sandstone, and conglomerate are found locally. Basalt sources are located approximately 5–10 km to the north and the northeast. Quartzite and schist sources are found 30–50 km to the south. Granite occurrences are not signalled in the geological map. However, it can be found in Precambrian to Ordovician formations present in the region [89]. Allochthonous rocks displaced by fluvial transport may have been collected along the wadi Laga Dächatu. However, collection from such deposits implies transport of the raw materials from the wadi floor to the site, an uphill transport of 400 m with a slope of 140 m. Furthermore, twelve grindstones (OPT 1, 2, 6, 7, 9–12, 14, 16–18), including some made of raw material not available locally, display clean edges–a characteristic incompatible with prolonged fluvial transport. The diversified nature of the rock types, the distant origin of some of them, and the fact that tools made of different rocks were found in the same area and at the same depth, indicates that ochre reduction sequences carried out at Porc-Epic required grindstones made of different rocks, which the site inhabitants acquired when moving across the landscape or through exchange with neighbouring groups. Porc-Epic represents the earliest known case in which the use of diversified rock types for ochre treatment is documented.

How was ochre powder produced? Iron-rich rock clasts may have been first crushed to reduce their size. The resulting fragments were probably abraded directly on lower grindstones, or reduced into powder by pounding/grinding them with upper grindstones. The latter is the technique used at present, for example, by Ovahimba women [123]. At Porc-Epic, all objects identified as upper grindstones display percussion pits reflecting pounding but virtually no facets or striations indicating their actual use to grind ochre. This suggests that ochre clasts were in some instances fragmented to reduce their size before being individually rubbed against lower grindstones rather than ground between lower and upper grindstones. This is consistent with preliminary analyses conducted on ochre fragments, which identify abrasion facets on numerous pieces [124]. Additionally, smoothed areas on lower grindstones made of hard rocks such as quartzite or basalt suggest a long-term use of these tools for rubbing ochre. The fact that only one lower grindstone (OPT 1), made of limestone, shows percussion pits associated with ochre residues does not contradict the hypothesis that ochre was in some instances crushed by pounding it on lower grindstones before being abraded. This activity may have been carried elsewhere, only on a reduced number of ochre fragments, or may have left little or no trace on lower grindstones made of hard rocks [125].

Why did Porc-Epic inhabitants use different rocks for their grindstones? The coarseness and hardness of grinding tools conditions the granulometry, shade, and composition of the ochre powder [19]. A grindstone made of a relatively soft rock such as limestone or sandstone releases a powder that is incorporated into the ochre powder produced by the grinding process [126]. The colour of the powder is generally lighter when using those tools. The same technique using grindstones made of hard rocks such as schist, quartzite, basalt, granitoid, or fine-grained ferruginous rocks, results in little or virtually no release and incorporation of particles derived from the tool. When using these rocks, the granulometry of the powder mostly depends on the roughness of the tool surface, as well as the pressure exerted during the work.

The above considerations, and the variety of rock types used for grindstones suggest that the MSA inhabitants of this site may have produced, according to their needs, ochre powders of different shades, consistency, composition, granulometry and colour. This is confirmed by the analysis of the residues collected on the grindstones, which reveals the use of different ochre types and, in many cases, the presence of more than one type on the same processing tool. The number of processed ochre types per tool is probably underestimated as sampled residues represent a minimal part of the ochre present on each processing tool, which is not necessarily representative of all iron-rich rocks processed on it. Such palimpsests of ochre types may result either from intentional grinding and mixing of different types of ochre at the same time, or, more probably, different ochre processing episodes. Whatever the case, the elemental and mineralogical analysis of the residues clearly indicates that different iron-rich minerals, with different properties, iron oxides types, and iron contents were sought after and processed.

What are the reasons behind such a high behavioural variability? Recent studies [19] have reached the conclusion that differences in granulometry, consistency and colour intensity of ochre powder can be related to its function. Fine, clayish sorted ochre powder is more suitable for cosmetic or symbolic activities such as body painting, whereas mixed grain size ochre would be more adapted to utilitarian activities such as hafting [6]. The use of grinding tools made of different raw materials, allowing the production of powders of different granulometries, the identification of different types of ochre in the residues collected on the ochre processing tools and among the ochre fragments recovered at the site [124] indicate that ochre was processed at Porc-Epic Cave to perform a variety of activities. Analysis of OSA 5 suggest that some of these activities may have been symbolic in nature. Considering the absence on this object of use-wear related to ochre processing, two hypotheses account for the red spot present on its surface: decoration of the pebble with red paint or use as a stamp or “pintadera” [127] to produce a subcircular print on soft surfaces such as human or animal skin. Both uses better fit symbolic rather than utilitarian functions. Supplementary evidence that, in some instances, a small quantity of a liquid binder was added to ground ochre powder comes from OPT 11, which displays a ring-like deposit resulting from the drying of such mixture. Toolkits to produce and store ochered liquid compounds were recovered at Blombos Cave in layers dated to 100 ka BP [5]. The use of ochre to change the appearance of personal ornaments is known at MSA sites from Northern and Southern Africa dated to ca. 80–70 ka BP [33,128]. The production of ochre and milk paint has been recently identified on residue adhering to a stone flake found at Sibudu Cave, South Africa, in layers dated to 49 ka BP [129]. Apart from the colouring of ornaments, however, virtually nothing is known about other non-utilitarian activities in which ochre may have been involved in the MSA. OSA 5 represents the earliest known example of a painted object from an MSA context or evidence that pigment powder was mixed with a liquid binder to produce a paint used to print red spots, perhaps arranged to create recognizable and meaningful visible patterns. Future experiments may clarify which of these two possibilities is the most likely. They may also document the elemental and mineralogical composition of powder produced by grinding different iron-rich rocks on grindstones made of a variety of raw materials. This would allow a better characterisation and quantification of the grindstone contribution to the resulting powder.

In conclusion, Porc-Epic is at present the only Palaeolithic site from the Horn of Africa that has yielded a collection of ochre pieces and processing tools allowing comprehensive documentation of practices related to ochre acquisition, processing, and use. The analysis of the processing tools and ochre-stained artefacts conducted in this study needs to be complemented in the future by the analysis of the ochre fragments found in the same layers. However, the results presented here already reveal an original, hitherto unknown, behavioural complexity. This complexity is evidenced by the diversity and exogenous provenance of the raw material used for the processing tools, the techniques and motions applied to modify iron-rich rocks, the variety of rocks chosen to produce ochre powder of different colour, composition, consistency and shade, and their probable involvement in utilitarian as well as symbolic activities.

To counter the notion that ochre was symbolically used in the MSA and the Mousterian, it has been argued that utilitarian functions suffice to explain the evidence. The opposition symbolic vs. functional represents, however, a false dichotomy, as, firstly, symbolic behaviour is more often than not embedded in what, to the western eye, would seem to be purely functional activities and, secondly, ochre may have been used in the context of both "symbolic" and “utilitarian” actions. The detailed analysis of the tools used to process ochre and the ochre residues still attached to them has the potential to advance the debate beyond this false either/or dichotomy.

## Supporting Information

S1 Fig Results of analyses conducted on ochre processing tools 1–4; 6–11 and ochre-stained artefact 5. Photos of the artefacts, modification marks and residues, SEM-EDS images and XRD diffractograms. The objects' identification number is the same as presented in Figs 4–11, Tables 1–5, S2 Fig, S1 Table, S1 Text.
(PDF)

S2 Fig Results of analyses conducted on ochre processing tools 12–14; 16–23 and ochre-stained artefact 15. Photos of the artefacts, modification marks and residues, SEM-EDS images and XRD diffractograms. The objects' identification number is the same as presented in Figs 4–11, Tables 1–5, S1 Fig, S1 Table, S1 Text.
(PDF)

S1 Table Detailed results of elemental and mineralogical analyses conducted on ochre processing tools and ochre-stained artefacts. SEM-EDS and μ-RS analyses. The objects' identification number is the same as presented in Figs 4–11, Tables 1–5, S1 and S2 Figs, S1 Text.
(PDF)

S1 Text Results of residue analysis on ochre processing tools and ochre-stained artefacts from Porc-Epic Cave. SEM-EDS, μ-RS and XRD analyses. The objects' identification number is the same as presented in Figs 4–11, Tables 1–5, S1 and S2 Figs, S1 Table.
(PDF)

## References

[1] Barham LS. Systematic Pigment Use in the Middle Pleistocene of South‐Central Africa. Curr Anthropol. 2002;43(1): 181–190. 10.1086/338292

[2] d’Errico F, García Moreno R, Rifkin RF. Technological, elemental and colorimetric analysis of an engraved ochre fragment from the Middle Stone Age levels of Klasies River Cave 1, South Africa. J Archaeol Sci. 2012;39(4): 942–952. 10.1016/j.jas.2011.10.032

[3] d’Errico F, Stringer CB. Evolution, revolution or saltation scenario for the emergence of modern cultures? Philos Trans R Soc B Biol Sci. 2011;366(1567): 1060–1069. 10.1098/rstb.2010.0340 21357228 PMC3049097

[4] Dayet L, Texier P-J, Daniel F, Porraz G. Ochre resources from the Middle Stone Age sequence of Diepkloof Rock Shelter, Western Cape, South Africa. J Archaeol Sci. 2013;40(9): 3492–3505. 10.1016/j.jas.2013.01.025

[5] Henshilwood CS, d’Errico F, Van Niekerk KL, Coquinot Y, Jacobs Z, Lauritzen S-E, et al A 100,000-Year-Old Ochre-Processing Workshop at Blombos Cave, South Africa. Science. 2011;334(6053): 219–222. 10.1126/science.1211535 21998386

[6] Hodgskiss T. An investigation into the properties of the ochre from Sibudu, KwaZulu-Natal, South Africa. South Afr Humanit. 2012;24(1): 99–120.

[7] Marean CW, Bar-Matthews M, Bernatchez J, Fisher E, Goldberg P, Herries AIR, et al Early human use of marine resources and pigment in South Africa during the Middle Pleistocene. Nature. 2007;449(7164): 905–908. 10.1038/nature06204 17943129

[8] McBrearty S, Brooks AS. The revolution that wasn’t: a new interpretation of the origin of modern human behavior. J Hum Evol. 2000;39(5): 453–563. 10.1006/jhev.2000.0435 11102266

[9] Watts I. The pigments from Pinnacle Point Cave 13B, Western Cape, South Africa. J Hum Evol. 2010;59(3–4): 392–411. 10.1016/j.jhevol.2010.07.006 20934093

[10] Bordes F. Sur l’usage probable de la peinture corporelle dans certaines tribus moustériennes. Bull Société Préhistorique Fr. 1952;49(3): 169–171.

[11] Dayet L, d’Errico F, Garcia-Moreno R. Searching for consistencies in Châtelperronian pigment use. J Archaeol Sci. 2014;44: 180–193. 10.1016/j.jas.2014.01.032

[12] Peyrony D. Le Moustier: ses gisements, ses industries, ses couches géologiques. Rev Anthropol. 1930;40: 48–76, 155–176.

[13] Roebroeks W, Sier MJ, Nielsen TK, De Loecker D, Parés JM, Arps CES, et al Use of red ochre by early Neandertals. Proc Natl Acad Sci U S A. 2012;109(6): 1889–1894. 10.1073/pnas.1112261109 22308348 PMC3277516

[14] Salomon H. Les matières colorantes au début du Paléolithique supérieur: sources, transformations et fonctions. Doctoral dissertation, Université Bordeaux 1. 2009.

[15] Soressi M, d’Errico F. Pigments, gravures, parures: les comportements symboliques controversés des Néandertaliens In: Vandermeersch B, Maureille B, editors. Les Néandertaliens: Biologie et Cultures. Paris: Editions du CTHS; 2007 p. 297–309.

[16] Zilhão J, Angelucci DE, Badal-García E, d’Errico F, Daniel F, Dayet L, et al Symbolic use of marine shells and mineral pigments by Iberian Neandertals. Proc Natl Acad Sci. 2010;107(3): 1023–1028. 10.1073/pnas.0914088107 20080653 PMC2824307

[17] d’Errico F, Salomon H, Vignaud C, Stringer C. Pigments from the Middle Palaeolithic levels of Es-Skhul (Mount Carmel, Israel). J Archaeol Sci. 2010;37(12): 3099–3110. 10.1016/j.jas.2010.07.011

[18] Hovers E, Ilani S, Bar‐Yosef O, Vandermeersch B. An Early Case of Color Symbolism: Ochre Use by Modern Humans in Qafzeh Cave. Curr Anthropol. 2003;44(4): 491–522. 10.1086/375869

[19] Rifkin RF. Processing ochre in the Middle Stone Age: Testing the inference of prehistoric behaviours from actualistically derived experimental data. J Anthropol Archaeol. 2012;31(2): 174–195. 10.1016/j.jaa.2011.11.004

[20] Watts I. Ochre in the Middle Stone Age of Southern Africa: Ritualised Display or Hide Preservative? South Afr Archaeol Bull. 2002;57(175): 1–14. 10.2307/3889102

[21] Zilhão J. The Emergence of Ornaments and Art: An Archaeological Perspective on the Origins of “Behavioral Modernity”. J Archaeol Res. 2007;15(1): 1–54. 10.1007/s10814-006-9008-1

[22] Kozowyk PRB, Langejans G, Poulis JA. Lap Shear and Impact Testing of Ochre and Beeswax in Experimental Middle Stone Age Compound Adhesives. PLoS ONE. 2016;11(3): e0150436 10.1371/journal.pone.0150436 26983080 PMC4794155

[23] Rifkin R. Assessing the efficacy of red ochre as a prehistoric hide tanning ingredient. J Afr Archaeol. 2011;9(2): 131–58. 10.3213/2191-5784-10199

[24] Rifkin RF, d’Errico F, Dayet-Boulliot L, Summers B. Assessing the photoprotective effects of red ochre on human skin by in vitro laboratory experiments. South Afr J Sci. 2015;111(3–4): 1–8. 10.17159/sajs.2015/20140202

[25] Rossano MJ. Making friends, making tools, and making symbols. Curr Anthropol. 2010;51(S1): S89–S98. 10.1086/650481

[26] Wadley L. Putting ochre to the test: replication studies of adhesives that may have been used for hafting tools in the Middle Stone Age. J Hum Evol. 2005;49(5): 587–601. 10.1016/j.jhevol.2005.06.007 16126249

[27] Wadley L, Hodgskiss T, Grant M. Implications for complex cognition from the hafting of tools with compound adhesives in the Middle Stone Age, South Africa. Proc Natl Acad Sci. 2009;106(24): 9590–9594. 10.1073/pnas.0900957106 19433786 PMC2700998

[28] Wadley L, Williamson B, Lombard M. Ochre in hafting in Middle Stone Age southern Africa: a practical role. Antiquity. 2004;78(301): 661–675. 10.1017/S0003598X00113298

[29] Wynn T, Coolidge FL. Beyond Symbolism and Language: An Introduction to Supplement 1, Working Memory. Curr Anthropol. 2010;51(S1): S5–S16. 10.1086/650526

[30] Williamson BS. Middle Stone Age tool function from residue analysis at Sibudu Cave. South Afr J Sci. 2004;100(3): 174–178.

[31] Gibson NE, Wadley L, Williamson BS. Microscopic residues as evidence of hafting on backed tools from the 60 000 to 68 000 year-old Howiesons Poort layers of Rose Cottage Cave, South Africa. South Afr Humanit. 2004;16: 1–11.

[32] d’Errico F, Vanhaeren M, Barton N, Bouzouggar A, Mienis H, Richter D, et al Additional evidence on the use of personal ornaments in the Middle Paleolithic of North Africa. Proc Natl Acad Sci. 2009;106(38): 16051–16056. 10.1073/pnas.0903532106 19717433 PMC2752514

[33] d’Errico F, Backwell L. Earliest evidence of personal ornaments associated with burial: The *Conus* shells from Border Cave. J Hum Evol. 2016;93: 91–108. 10.1016/j.jhevol.2016.01.002 27086058

[34] Peresani M, Vanhaeren M, Quaggiotto E, Queffelec A, d’Errico F. An Ochered Fossil Marine Shell From the Mousterian of Fumane Cave, Italy. PLoS ONE. 2013;8(7): e68572 10.1371/journal.pone.0068572 23874677 PMC3707824

[35] Vanhaeren M, D’Errico F, Stringer C, James SL, Todd JA, Mienis HK. Middle paleolithic shell beads in Israel and Algeria. Science. 2006;312(5781): 1785–1788. 10.1126/science.1128139 16794076

[36] Desmond Clark J, Williamson KD. A Middle Stone Age occupation site at Porc Epic Cave, Dire Dawa (east-central Ethiopia), Part I. Afr Archaeol Rev. 1984;2(1): 37–64. 10.1007/BF01117225

[37] Rosso DE, d’Errico F, Zilhão J. Stratigraphic and spatial distribution of ochre and ochre processing tools at Porc-Epic Cave, Dire Dawa, Ethiopia. Quat Int. 2014;343: 85–99. 10.1016/j.quaint.2013.10.019

[38] Tryon CA, Faith JT. Variability in the Middle Stone Age of Eastern Africa. Curr Anthropol. 2013;54(S8): S234–S254. 10.1086/673752

[39] Desmond Clark J, Brown KS. The Twin Rivers Kopje, Zambia: Stratigraphy, Fauna, and Artefact Assemblages from the 1954 and 1956 Excavations. J Archaeol Sci. 2001;28(3): 305–330. 10.1006/jasc.2000.0563

[40] Van Peer P, Rots V, Vroomans J-M. A story of colourful diggers and grinders. Farming. 2004;2004/3 article 1: 1–28. 10.3828/bfarm.2004.3.1

[41] Van Peer P, Fullagar R, Stokes S, Bailey RM, Moeyersons J, Steenhoudt F, et al The Early to Middle Stone Age transition and the emergence of modern human behaviour at site 8-B-11, Sai Island, Sudan. J Hum Evol. 2003;45(2): 187–193. 10.1016/S0047-2484(03)00103-9 14529653

[42] Mercader J. Mozambican Grass Seed Consumption During the Middle Stone Age. Science. 2009;326(5960): 1680–1683. 10.1126/science.1173966 20019285

[43] Mercader J, Asmerom Y, Bennett T, Raja M, Skinner A. Initial excavation and dating of Ngalue Cave: A Middle Stone Age site along the Niassa Rift, Mozambique. J Hum Evol. 2009;57(1): 63–74. 10.1016/j.jhevol.2009.03.005 19487015

[44] Singer R, Wymer J. The Middle Stone Age at Klasies River Mouth in South Africa. Chicago: University of Chicago Press; 1982.

[45] Avery G, Cruz-Uribe K, Goldberg P, Grine FE, Klein RG, Lenardi MJ, et al The 1992–1993 Excavations at the Die Kelders Middle and Later Stone Age Cave Site, South Africa. J Field Archaeol. 1997;24(3): 263–291. 10.2307/530685

[46] Grine FE, Klein RG, Volman TP. Dating, archaeology and human fossils from the Middle Stone Age levels of Die Kelders, South Africa. J Hum Evol. 1991;21(5): 363–395. 10.1016/0047-2484(91)90113-A

[47] Thackeray AI. Middle Stone Age artefacts from the 1993 and 1995 excavations of Die Kelders Cave 1, South Africa. J Hum Evol. 2000;38(1): 147–168. 10.1006/jhev.1999.0354 10627401

[48] Wadley L. Cemented ash as a receptacle or work surface for ochre powder production at Sibudu, South Africa, 58,000 years ago. J Archaeol Sci. 2010;37(10): 2397–2406. 10.1016/j.jas.2010.04.012

[49] Soriano S, Villa P, Wadley L. Ochre for the Toolmaker: Shaping the Still Bay Points at Sibudu (KwaZulu-Natal, South Africa). J Afr Archaeol. 2009;7(1): 41–54.

[50] Avery G, Halkett D, Orton J, Steele T, Tunesius M, Klein R. The Ysterfontein 1 Middle Stone Age Rock Shelter and the evolution of coastal foraging. South Afr Archaeol Soc Goodwin Ser. 2008;10: 66–89.

[51] Kaplan J. The Umhlatuzana Rock Shelter sequence: 100 000 years of Stone Age history. South Afr Humanit. 1990;2: 1–94.

[52] de Beaune S. Origine du matériel de broyage au Paléolithique. In: Procopiou H, Treuil R, editors. Moudre et broyer: L’interprétation fonctionnelle de l’outillage de mouture et de broyage dans la Préhistoire et l’Antiquité. Actes de la table ronde internationale; 1995 Nov 30-Dec 2; Clermont-Ferrand, France. Paris: Editions du CTHS; 2002. p. 22–53.

[53] de Beaune S. Nonflint stone tools of the early Upper Paleolithic In: Knecht H, Pike-Tay A, White, editors. Before Lascaux: The Complex Record of the Early Upper Paleolithic. Boca Raton: CRC Press; 1993 p. 137–162.

[54] Kraybill N. Pre-Agricultural Tools for the Preparation of Foods in the Old World In: Reed CA, editor. Origins of Agriculture. The Hague: Walter de Gruyter; 1977 p. 485–522.

[55] Louw AW. Bushman Rock Shelter, Ohrigstad, Eastern Transvaal: A Preliminary Investigation, 1965. South Afr Archaeol Bull. 1969;24(94): 39–51. 10.2307/3887660

[56] Carter PL, Mitchell PJ, Vinnicombe P. Sehonghong: The Middle and Later Stone Age Industrial Sequence at a Lesotho Rock-shelter. Oxford: British Archaeological Reports, International series;406: Archaeopress; 1988.

[57] Huysecom E, Ozainne S, Robion-Brunner C, Mayor A, Ballouche A, Chaix L, et al Nouvelles données sur le peuplement du Pays dogon: la onzième année de recherches du programme “Peuplement humain et évolution paléoclimatique en Afrique de l’Ouest” Jahresbericht SLSA 2008. Zürich et Vaduz: Fondation Suisse-Liechtenstein pour les recherches archéologiques à l’étranger; 2009 p. 71–183.

[58] Huysecom E, Robion-Brunner C, Mayor A, Ozainne S, Ballouche A, Cissé L, et al La dixième année de recherche du programme “Peuplement humain et évolution paléoclimatique en Afrique de l’Ouest” Jahresbericht SLSA 2007. Zürich et Vaduz: Fondation Suisse-Liechtenstein pour les recherches archéologiques à l’étranger; 2008 p. 43–140.

[59] Larsson L. The Middle Stone Age of Zimbabwe: some aspects of former research and future aims In: Pwiti G, Soper R, editors. Aspects of African Archaeology. Harare: University of Zimbabwe Publications; 1996 p. 201–211.

[60] Walker NJ. Late Pleistocene and Holocene Hunter-Gatherers of the Matopos: An Archaeological Study of Change and Continuity in Zimbabwe Studies in African Archaeology. Uppsala: Societas Archaeologica Upsaliensis; 1995.

[61] Ambrose SH. Chronology of the Later Stone Age and Food Production in East Africa. J Archaeol Sci. 1998;25(4): 377–392. 10.1006/jasc.1997.0277

[62] Brandt SA, Fisher EC, Hildebrand EA, Vogelsang R, Ambrose SH, Lesur J, et al Early MIS 3 occupation of Mochena Borago Rockshelter, Southwest Ethiopian Highlands: Implications for Late Pleistocene archaeology, paleoenvironments and modern human dispersals. Quat Int. 2012;274: 38–54. 10.1016/j.quaint.2012.03.047

[63] Mehlman MJ. Late Quaternary archaeological sequences in northern Tanzania. Doctoral dissertation, University of Illinois. 1989.

[64] Taborin Y. La mer et les premiers hommes modernes In: Vandermeersch B, editor. Echanges et Diffusion dans la Préhistoire Méditerranéenne. Paris: Editions du CTHS; 2003 p. 113–122.

[65] Bar-Yosef Mayer DE, Vandermeersch B, Bar-Yosef O. Shells and ochre in Middle Paleolithic Qafzeh Cave, Israel: indications for modern behavior. J Hum Evol. 2009;56(3): 307–314. 10.1016/j.jhevol.2008.10.005 19285591

[66] Marshack A. On Paleolithic Ochre and the Early Uses of Color and Symbol. Curr Anthropol. 1981;22(2): 188–191. 10.1086/202650

[67] Šajnerová-Dušková A, Fridrich J, Fridrichová-Sýkorová I. Pitted and grinding stones from Middle Palaeolithic settlements in Bohemia: a functional study. In: Sternke F, Costa LJ, Eigeland L, editors. Non-flint Raw Material Use in Prehistory: Old Prejudices and New Directions. Proceedings of the XV Congress of the UISPP; 2006 Sept 4–9; Lisbon, Portugal. Oxford: British Archaeological Reports, International Series;1939: Archaeopress; 2009. p. 1–10.

[68] Thieme H. Schleif-oder Reibplatten des fundplatzes Rheindahlen-Ostecke, Ziegeleigrube Dressen, Stadtkr. Monchengladbach in Festschrift Hermann Schwabedissen. Teil I: Beiträge zum Paläolithikum und Neolithikum. Kolner Jahrb Für Vor- Frühgesch Berl. 1975;15: 24–30.

[69] Cârciumaru M, Niţu E-C, Nicolae A, Ionuț Lupu F, Dincă R. Contributions to understanding the Neanderthals symbolism. Examples from the Middle Paleolithic in Romania. Ann D’Université Valahia Targoviste. 2015;XVII(2): 7–31.

[70] Cârciumaru M, Niţu E-C, Cîrstina O. A geode painted with ochre by the Neanderthal man. Comptes Rendus Palevol. 2014;14: 31–41. 10.1016/j.crpv.2014.05.003

[71] Cârciumaru M, Ţuţuianu-Cârciumaru M. L’ocre et les recipients pour ocre de la grotte Cioarei, village Borosteni, commune Pestisani, dep. de Gorj, Roumanie. Ann D’Université Valahia Targoviste. 2009;XI(1): 7–19.

[72] Hoffecker JF. Desolate Landscapes: Ice-Age Settlement in Eastern Europe. Piscataway, NJ: Rutgers University Press; 2002.

[73] Smith RF. An individual-based comparative advantage model: did economic specialization mediate the fluctuating climate of the late Pleistocene during the transition from Neanderthals to modern humans? Doctoral dissertation, Rutgers, The State University of New Jersey. 2007.

[74] d’Errico F. Le rouge et le noir: implications of early pigment use in Africa, the Near East and Europe for the origin of cultural modernity. South Afr Archaeol Soc Goodwin Ser. 2008;10: 168–174.

[75] Heyes PJ, Anastasakis K, de Jong W, van Hoesel A, Roebroeks W, Soressi M. Selection and Use of Manganese Dioxide by Neanderthals. Sci Rep. 2016;6: 22159 10.1038/srep22159 26922901 PMC4770591

[76] Soressi M, Rendu W, Texier J-P, Claud E, Daulny L, d’Errico F, et al Pech-de-l’Azé I (Dordogne, France): nouveau regard sur un gisement moustérien de tradition acheuléenne connu depuis le 19ème siècle. Mém Société Préhistorique Fr. 2009;47: 95–132.

[77] Combier F. Soyons, Grotte de Néron (Ardèche) In: Mohen J-P, Olivier L, editors. Archéologie de la France 30 ans de découvertes. Paris: Editions de la Réunion des Musées Nationaux; 1989.

[78] Caron F, d’Errico F, Del Moral P, Santos F, Zilhão J. The Reality of Neandertal Symbolic Behavior at the Grotte du Renne, Arcy-sur-Cure, France. PLoS ONE. 2011;6(1): e21545 10.1371/journal.pone.0021545 21738702 PMC3126825

[79] Clarkson C, Smith M, Marwick B, Fullagar R, Wallis LA, Faulkner P, et al The archaeology, chronology and stratigraphy of Madjedbebe (Malakunanja II): A site in northern Australia with early occupation. J Hum Evol. 2015;83: 46–64. 10.1016/j.jhevol.2015.03.014 25957653

[80] Roberts RG, Jones R, Spooner NA, Head MJ, Murray AS, Smith MA. The human colonisation of Australia: optical dates of 53,000 and 60,000 years bracket human arrival at Deaf Adder Gorge, Northern Territory. Quat Sci Rev. 1994;13: 575–583. 10.1016/0277-3791(94)90080-9

[81] Bird MI, Turney CSM, Fifield LK, Jones R, Ayliffe LK, Palmer A, et al Radiocarbon analysis of the early archaeological site of Nauwalabila I, Arnhem Land, Australia: implications for sample suitability and stratigraphic integrity. Quat Sci Rev. 2002;21: 1061–1075. 10.1016/S0277-3791(01)00058-0

[82] de Beaune S. Pour une archéologie du geste: Broyer, moudre, piler, des premiers chasseurs aux premiers agriculteurs Paris: CNRS Editions; 2000.

[83] de Beaune S. Essai d’une classification typologique des galets et plaquettes utilisés au Paléolithique. Gall Préhistoire. 1989;31: 27–64.

[84] San-Juan C. Les matières colorantes dans les collections du Musée National de Préhistoire des Eyzies. Paléo. 1990;2: 229–242.

[85] Teilhard de Chardin P. Le Paléolithique en Somalie française et en Abyssinie. Anthropologie. 1930;40: 331–334.

[86] Breuil H. Peintures rupestres préhistoriques du Harrar, Abyssinie. Anthropologie. 1934;44(2): 473–483.

[87] Teilhard de Chardin P, Breuil H, Wernert P. Les Industries lithiques de Somalie française. Anthropologie. 1940;49: 497–522.

[88] Desmond Clark J, Williams MAJ. Recent Archaeological Research in Southeastern Ethiopia. 1974–1975. Ann Ethiop. 1978;11(1): 19–44. 10.3406/ethio.1978.902

[89] Pleurdeau D. Gestion des matières premières et comportements techniques dans le middle stone age africain: les assemblages lithiques de la grotte du Porc-Épic, Dire Dawa, Éthiopie. Oxford: British Archaeological Reports, International Series;1317: Archaeopress; 2004.

[90] Assefa Z. Faunal remains from Porc-Epic: Paleoecological and zooarchaeological investigations from a Middle Stone Age site in southeastern Ethiopia. J Hum Evol. 2006;51(1): 50–75. 10.1016/j.jhevol.2006.01.004 16545861

[91] Leplongeon A. Microliths in the Middle and Later Stone Age of eastern Africa: New data from Porc-Epic and Goda Buticha cave sites, Ethiopia. Quat Int. 2014;343: 100–116. 10.1016/j.quaint.2013.12.002

[92] Leplongeon A. La transition Middle Stone Age / Later Stone Age en Afrique de l’Est (Ethiopie). Doctoral dissertation, Muséum National d’Histoire Naturelle. 2013.

[93] Perlès C. Réexamen Typologique de L’Industrie du Porc-Épic Éthiopie: Les Pointes et Pièces Pointues. Anthropologie. 1974;78(3): 529–551.

[94] Pleurdeau D. Human Technical Behavior in the African Middle Stone Age: The Lithic Assemblage of Porc-Epic Cave (Dire Dawa, Ethiopia). Afr Archaeol Rev. 2005;22(4): 177–197. 10.1007/s10437-006-9000-7

[95] Pleurdeau D. The lithic assemblage of the 1975–1976 excavation of the Porc-Epic Cave, Dire-Dawa, Ethiopia. Implications for the East African Middle Stone Age. J Afr Archaeol. 2005;3(1): 117–126. 10.3213/1612-1651-10040

[96] Pleurdeau D. Le Middle Stone Age de la grotte du Porc-Épic (Dire Dawa, Éthiopie): gestion des matières premières et comportements techniques. Anthropologie. 2003;107(1): 15–48. 10.1016/S0003-5521(02)00003-1

[97] Negash A, Shackley MS. Geochemical provenance of obsidian artefacts from the MSA site of Porc Epic, Ethiopia. Archaeometry. 2006;48(1): 1–12. 10.1111/j.1475-4754.2006.00239.x

[98] Vogel N, Nomade S, Negash A, Renne PR. Forensic 40Ar/39Ar dating: a provenance study of Middle Stone Age obsidian artifacts from Ethiopia. J Archaeol Sci. 2006;33(12): 1749–1765. 10.1016/j.jas.2006.03.008

[99] Vallois HV. La Mandibule Humaine Fossile de la Grotte du Porc-Épic près de Diré-Daoua Abyssinie. Anthropologie. 1951;55: 231–238.

[100] Assefa Z, Lam YM, Mienis HK. Symbolic Use of Terrestrial Gastropod Opercula during the Middle Stone Age at Porc-Epic Cave, Ethiopia. Curr Anthropol. 2008;49(4): 746–756. 10.1086/589509

[101] Michels JW, Marean CA. A Middle Stone Age occupation site at Porc Epic Cave, Dire Dawa (east-central Ethiopia), Part II. Afr Archaeol Rev. 1984;2(1): 64–71. 10.1007/BF01117225

[102] Anovitz LM, Elam JM, Riciputi LR, Cole DR. The Failure of Obsidian Hydration Dating: Sources, Implications, and New Directions. J Archaeol Sci. 1999;26(7): 735–752. 10.1006/jasc.1998.0342

[103] Ridings R. Where in the World Does Obsidian Hydration Dating Work? Am Antiq. 1996;61(1): 136 10.2307/282308

[104] Bronk Ramsey C. Radiocarbon calibration and analysis of stratigraphy; the OxCal program. Radiocarbon. 1995;37(2): 425–430.

[105] Breuil H, Teilhard de Chardin P, Wernert P. Le Paléolithique du Harrar. Anthropologie. 1951;55: 219–230.

[106] Adams J, Delgado S, Dubreuil L, Hamon C, Plisson H, Risch R. Functional analysis of macro-lithic artefacts. In: Sternke F, Costa LJ, Eigeland L, editors. Non-flint Raw Material Use in Prehistory: Old Prejudices and New Directions. Proceedings of the XV Congress of the UISPP; 2006 Sept 4–9; Lisbon, Portugal. Oxford: British Archaeological Reports, International Series;1939: Archaeopress; 2009. p. 43–66.

[107] Hamon C. Broyage et abrasion au Néolithique ancien. Caractérisation technique et fonctionnelle des outillages en grès du Bassin parisien. Oxford: British Archaeological Reports, International Series;1551: Archaeopress; 2006.

[108] Verbaas A, Van Gijn A. Querns and other hard stone tools from Geleen-Janskamperveld In: Van de Velde P, editor. Excavations at Geleen-Janskamperveld 1990/1991. Leiden: University of Leiden; 2008 p. 191–204.

[109] Dubreuil L. Long-term trends in Natufian subsistence: a use-wear analysis of ground stone tools. J Archaeol Sci. 2004;31(11): 1613–1629. 10.1016/j.jas.2004.04.003

[110] Dubreuil L. Étude fonctionnelle des outils de broyage natoufiens: nouvelles perspectives sur l’émergence de l’agriculture au Proche-Orient. Doctoral dissertation, Université Bordeaux 1. 2002.

[111] Hamon C. Functional analysis of stone grinding and polishing tools from the earliest Neolithic of north-western Europe. J Archaeol Sci. 2008;35: 1502–1520. 10.1016/j.jas.2007.10.017

[112] Dubreuil L, Savage D, Delgado-Raack S, Plisson H, Stephenson B, de la Torre I. Current Analytical Frameworks for Studies of Use–Wear on Ground Stone Tools In: Marreiros JM, Bao JFG, Bicho NF, editors. Use-Wear and Residue Analysis in Archaeology. Springer International Publishing; 2015 p. 105–158.

[113] Dubreuil L, Savage D. Ground stones: a synthesis of the use-wear approach. J Archaeol Sci. 2014;48: 139–153. 10.1016/j.jas.2013.06.023

[114] Hodgskiss T. Identifying grinding, scoring and rubbing use-wear on experimental ochre pieces. J Archaeol Sci. 2010;37(12): 3344–3358. 10.1016/j.jas.2010.08.003

[115] Downs RT. The RRUFF Project: an integrated study of the chemistry, crystallography, Raman and infrared spectroscopy of minerals. Program and Abstracts of the 19th General Meeting of the International Mineralogical Association in Kobe, Japan. 2006. p. 3–13.

[116] Dubreuil L. Functional Studies of Prehistoric Grindingstones. Bull Cent Rech Fr à Jérusalem. 2001;9: 73–87.

[117] Goren-Inbar N, Sharon G, Alperson-Afil N, Herzlinger G. A new type of anvil in the Acheulian of Gesher Benot Ya’aqov, Israel. Philos Trans R Soc Lond B Biol Sci. 2015;370(1682): 20140353 10.1098/rstb.2014.0353 26483531 PMC4614716

[118] Logan EN, Fratt L. Pigment Processing at Homol’ovi III: A Preliminary Study. Kiva. 1993;58(3): 415–428.

[119] Deegan RD, Bakajin O, Dupont TF, Huber G, Nagel SR, Witten TA. Capillary flow as the cause of ring stains from dried liquid drops. Nature. 1997;389(6653): 827–829. 10.1038/39827

[120] Yunker PJ, Still T, Lohr MA, Yodh AG. Suppression of the coffee-ring effect by shape-dependent capillary interactions. Nature. 2011;476(7360): 308–311. 10.1038/nature10344 21850105

[121] Whitney DL, Evans BW. Abbreviations for names of rock-forming minerals. Am Mineral. 2010;95: 185–187.

[122] Tegegn F. Geological map of Dire Dawa, NC 37–12 [map]. Addis Ababa: Ethiopian Institute of Geological Surveys, Ministry of Mines and Energy; 1985.

[123] Rifkin RF, Dayet L, Queffelec A, Summers B, Lategan M, d’Errico F. Evaluating the Photoprotective Effects of Ochre on Human Skin by In Vivo SPF Assessment: Implications for Human Evolution, Adaptation and Dispersal. PloS One. 2015;10(9): e0136090 10.1371/journal.pone.0136090 26353012 PMC4564224

[124] Rosso DE. Le traitement des matières colorantes à la Grotte du Porc-Épic, Dire Dawa, Éthiopie. MSc. Thesis, Université Bordeaux 1. 2011.

[125] de la Torre I, Benito-Calvo A, Arroyo A, Zupancich A, Proffitt T. Experimental protocols for the study of battered stone anvils from Olduvai Gorge (Tanzania). J Archaeol Sci. 2013;40: 313–332. 10.1016/j.jas.2012.08.007

[126] Wadley L. Ochre crayons or waste products? Replications compared with MSA “crayons” from Sibudu Cave, South Africa. Farming. 2005;2005(3): 1–12. 10.3828/bfarm.2005.3.1

[127] Serradimigni M. Le pintaderas nel quadro del Neolitico italiano: arte, simbolismo e funzionalità. Preistoria Alp. 2012;46b: 181–188.

[128] d’Errico F, Henshilwood C, Vanhaeren M, Van Niekerk K. *Nassarius kraussianus* shell beads from Blombos Cave: evidence for symbolic behaviour in the Middle Stone Age. J Hum Evol. 2005;48(1): 3–24. 10.1016/j.jhevol.2004.09.002 15656934

[129] Villa P, Pollarolo L, Degano I, Birolo L, Pasero M, Biagioni C, et al A Milk and Ochre Paint Mixture Used 49,000 Years Ago at Sibudu, South Africa. PLoS ONE. 2015;10(6): e0131273 10.1371/journal.pone.0131273 26125562 PMC4488428

