# Supplementary material for: Middle Stone Age Ochre Processing and Behavioural Complexity in the Horn of Africa: Evidence from Porc-Epic Cave, Dire Dawa, Ethiopia
Source: PLoS One. 2016 Nov 2;11(11):e0164793. doi: 10.1371/journal.pone.0164793 (PMC5091854; doi:10.1371/journal.pone.0164793)
Supplement: S1 Fig — Photos of the artefacts, modification marks and residues, SEM-EDS images and XRD diffractograms. The objects' identification number is the same as presented in Figs 4–11, Tables 1–5, S2 Fig, S1 Table, S1 Text. (PDF) [file pone.0164793.s001.pdf]

Middle Stone Age Ochre Processing and Behavioural Complexity in the Horn of Africa: Evidence from Porc-Epic Cave, Dire Dawa, Ethiopia

Daniela Eugenia Rosso*, Africa Pitarch Martí, Francesco d'Errico

* Corresponding author
E-mail: d.rosso@pacea.u-bordeaux1.fr (DR)

S1 Figures. Results of analyses conducted on ochre processing tools 1–4; 6–11 and ochre-stained artefact 5.

Photos of the artefacts, modification marks and residues, SEM-EDS images and XRD diffractograms. The objects' identification number is the same as presented in Figs 4–11, Tables 1–5, S2 Figs, S1 Tables, S1 Texts.

Figure A. Results of analyses conducted on ochre processing tool 1 2
Figure B. Results of analyses conducted on ochre processing tool 2 3
Figure C. Results of analyses conducted on ochre processing tool 3 4
Figure D. Results of analyses conducted on ochre processing tool 4 5
Figure E. Results of analyses conducted on ochre-stained artefact 5............................6
Figure F. Results of analyses conducted on ochre processing tool 6 7
Figure G. Results of analyses conducted on ochre processing tool 7............................8
Figure H. Results of analyses conducted on ochre processing tool 8............................9
Figure I. Results of analyses conducted on ochre processing tool 9............................10
Figure J. Results of analyses conducted on ochre processing tool 10.........................11
Figure K. Results of analyses conducted on ochre processing tool 11 12

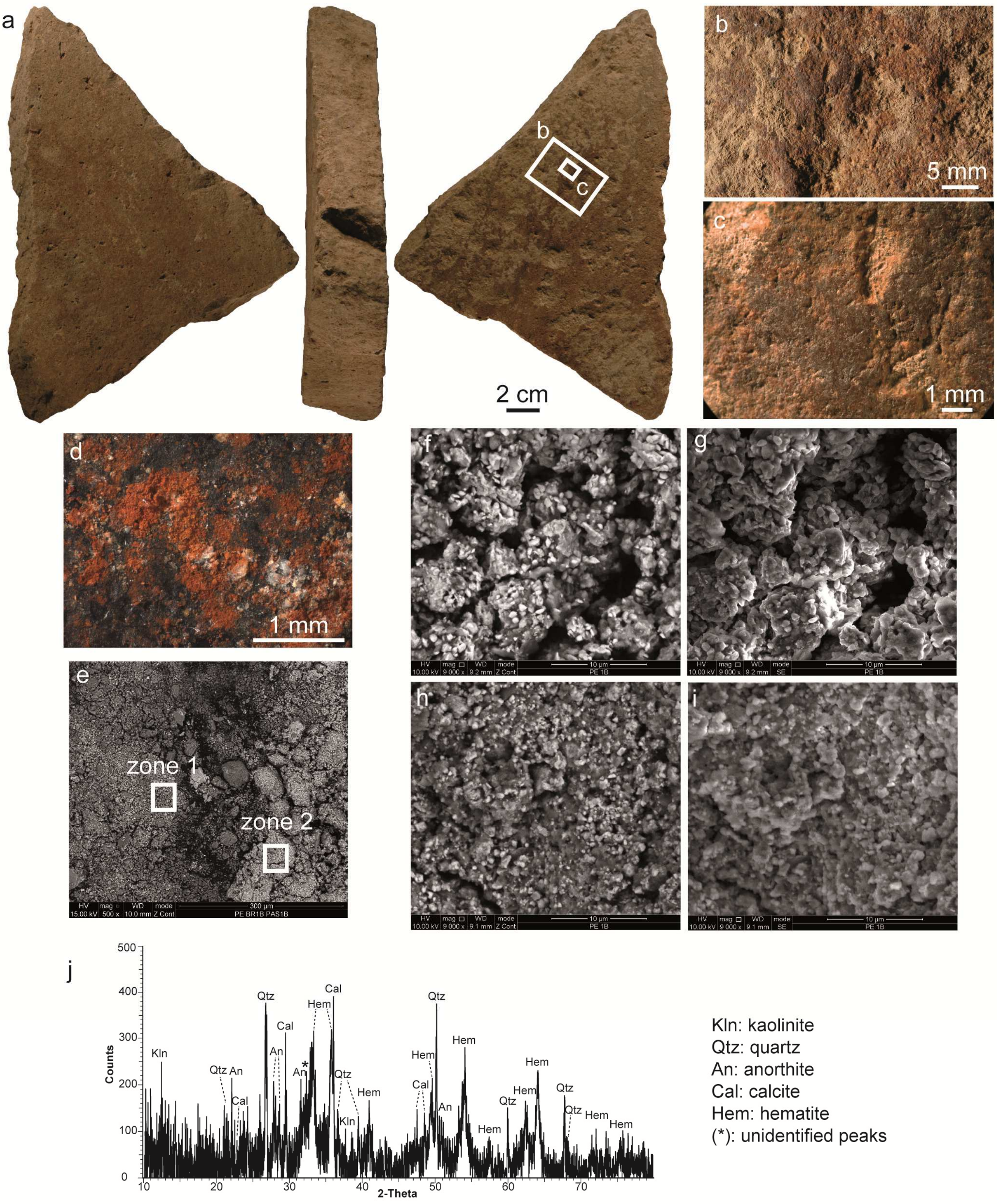

Fig A. Results of analyses conducted on ochre processing tool 1 (lower grindstone).

a: Photo of the object. Squares indicate the location of macro photos b and c; b, c: macro photos of smoothed area associated with pits and red residues; d: photo of the sampled ochre residue (sample AT1A); e: SEM image in BSE mode of sample AT1A (zones 1 and 2 indicate areas represented in f, g and h, i respectively); SEM images in BSE (f) and SE (g) modes of sample AT1A zone 1; SEM images in BSE (h) and SE (i) modes of sample AT1A zone 2; j: X-ray diffractogram of sample T1.

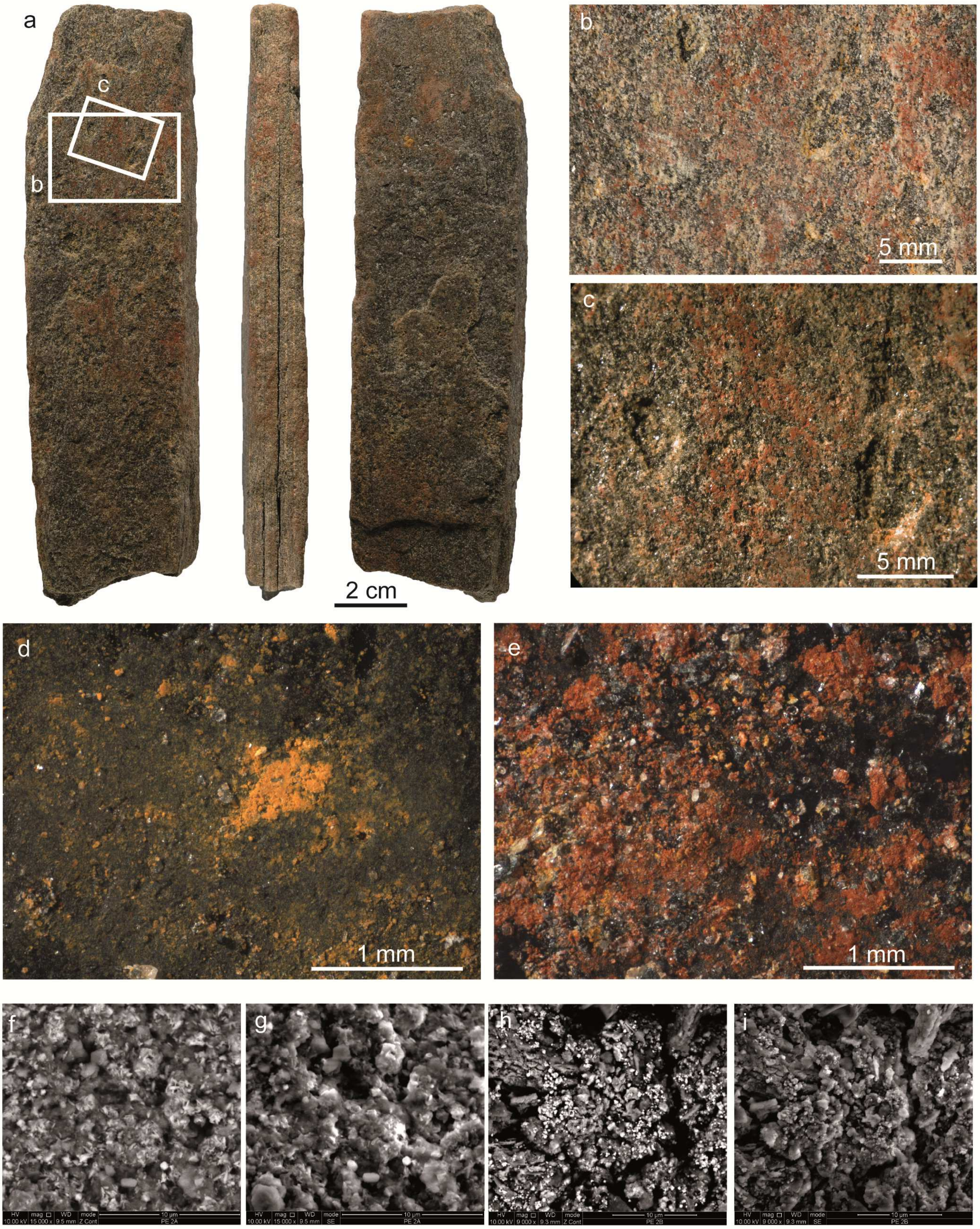

Fig B. Results of analyses conducted on ochre processing tool 2 (lower grindstone). a: Photo of the object. Squares indicate the location of macro photos b and c; b, c: macro photos of smoothed area associated with red and yellow residues; d, e: photos of the sampled ochre residues (samples AT2A and AT2B); SEM images in BSE (f) and SE (g) modes of sample AT2A; SEM images in BSE (h) and SE (i) modes of sample AT2B.

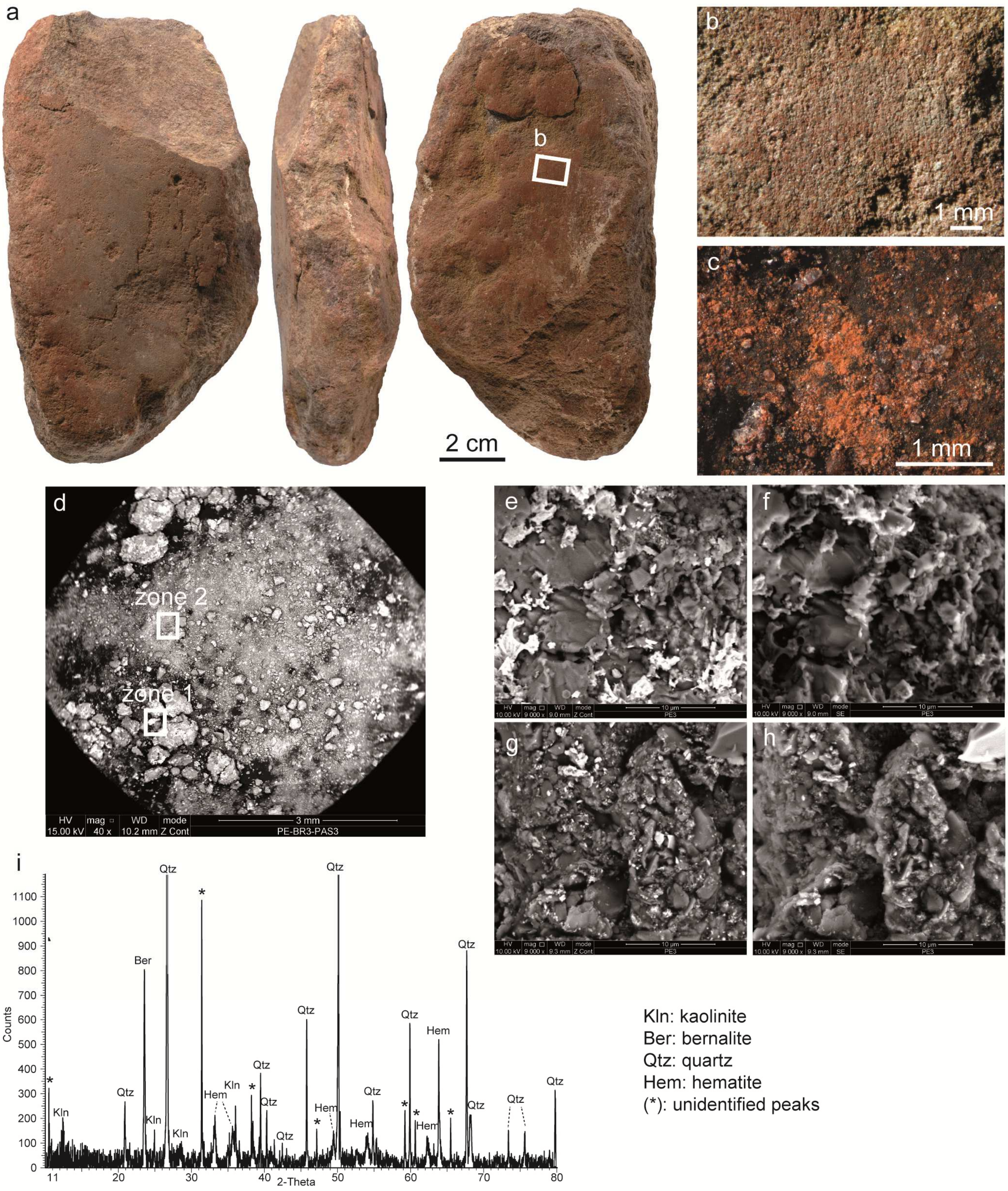

Fig C. Results of analyses conducted on ochre processing tool 3 (upper/lower grindstone).

a: Photo of the object. Square indicates the location of macro photo b; b: macro photo of smoothed areas associated with microstriations and red residues; c: photo of the sampled ochre residue (sample AT3); d: SEM image in BSE mode of sample AT3 (zones 1 and 2 indicate areas represented in e, f, and g, h respectively); SEM images in BSE (e) and SE (f) modes of sample AT3 zone 1; SEM images in BSE (g) and SE (h) modes of sample AT3 zone 2; i: X-ray diffractogram of sample T3.

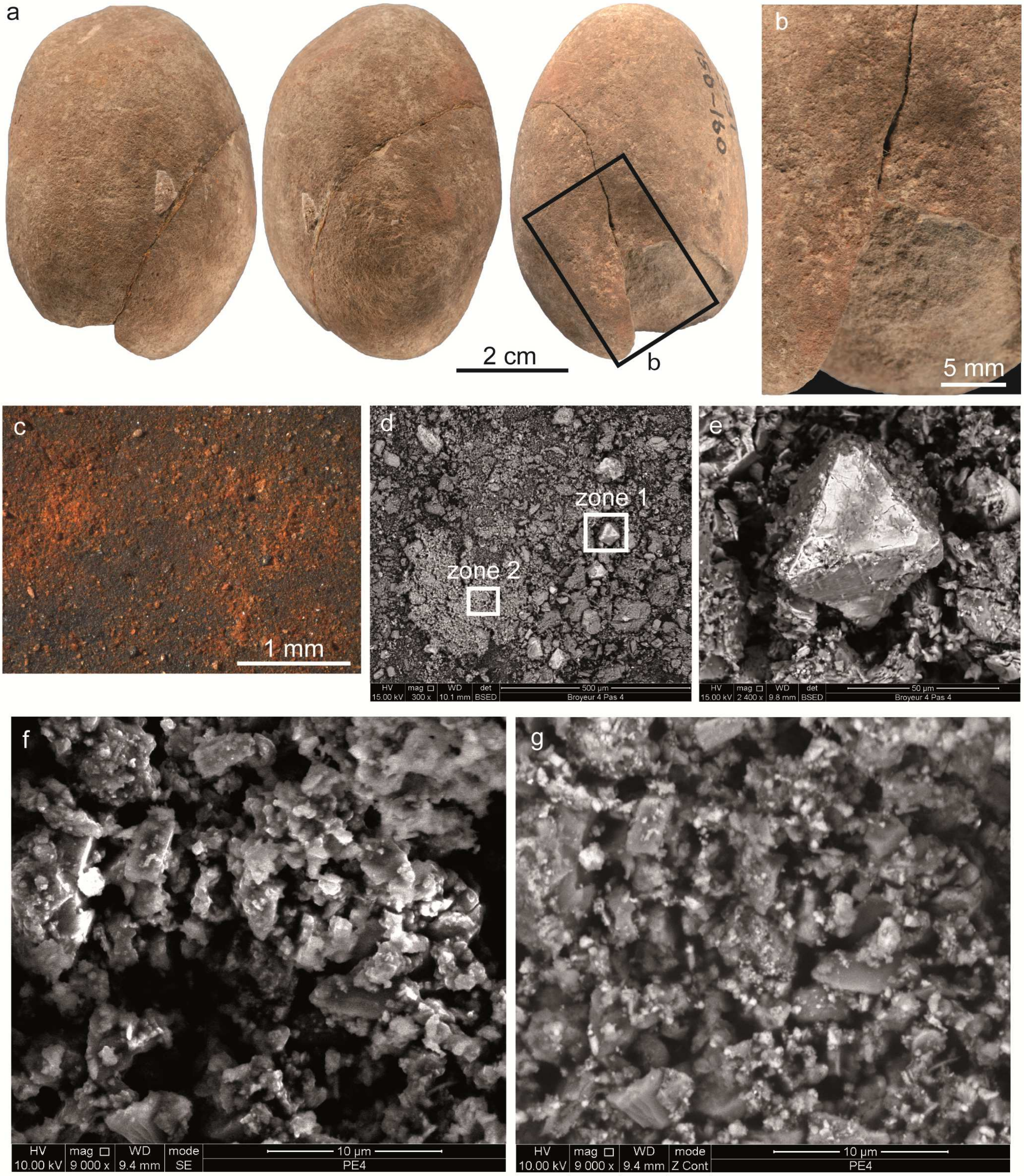

Fig D. Results of analyses conducted on ochre processing tool 4 (upper grindstone). a: Photo of the object. Square indicates the location of macro photo b; b: macro photo of pits associated with red residues; c: photo of the sampled ochre residue (sample AT4); d: SEM image in BSE mode of sample AT4 (zones 1 and 2 indicate areas represented in e and f, g respectively); e: SEM image in BSE mode of sample AT4 zone 1; SEM images in SE (f) and BSE (g) modes of sample AT4 zone 2.

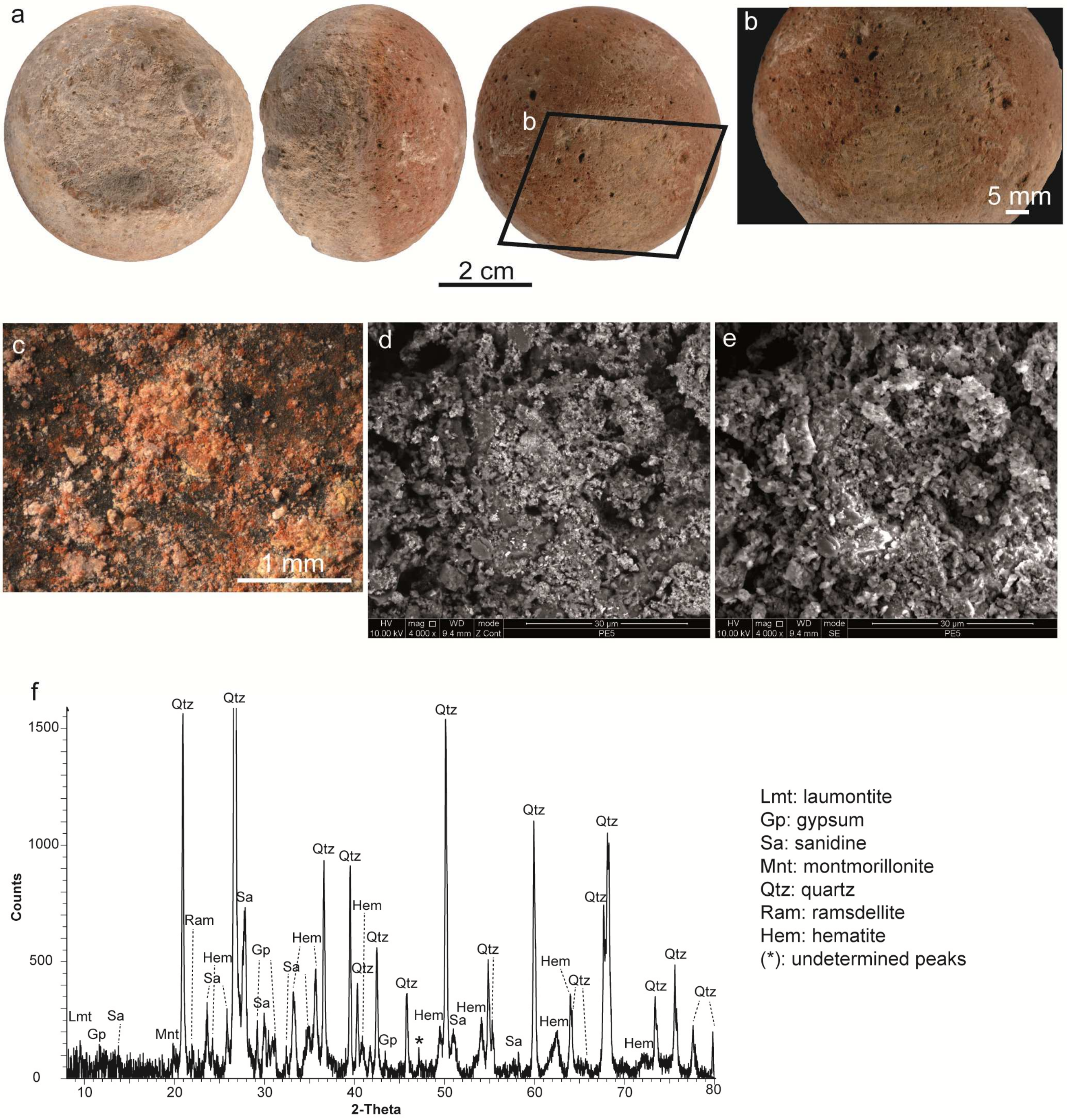

Fig E. Results of analyses conducted on ochre-stained artefact 5.
a: Photo of the object. Square indicates the location of macro photo b; b: macro photo of linear impressions associated with red residues; c: photo of the sampled ochre residue (sample AT5); SEM images in BSE (d) and SE (e) modes of sample AT5; f: X-ray diffractogram of sample T5.

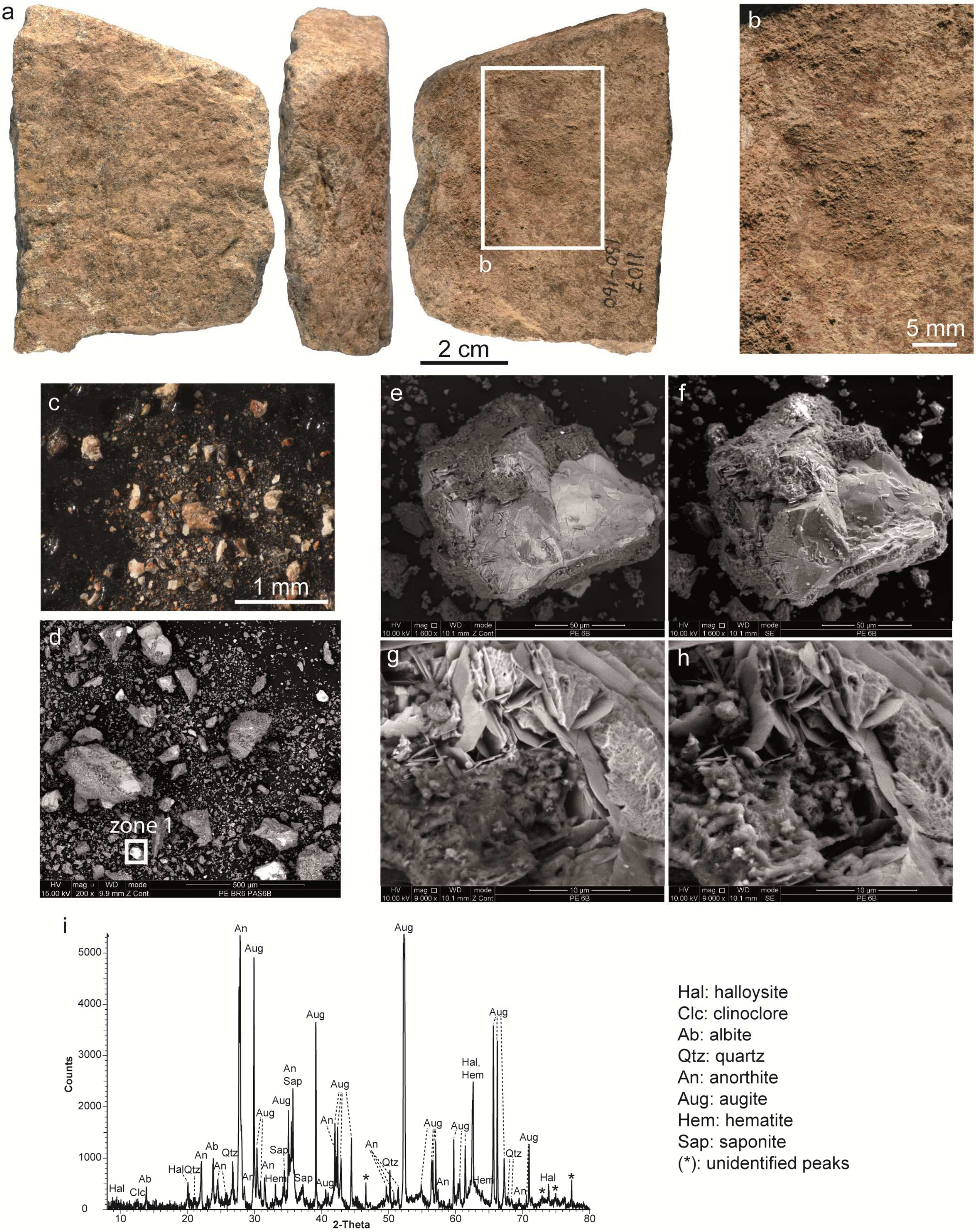

Fig F. Results of analyses conducted on ochre processing tool 6 (lower grindstone). a: Photo of the object. Square indicates the location of macro photo b; b: macro photo of smoothed area associated with red residues; c: photo of the sampled ochre residue (sample AT6); d: SEM image in BSE mode of sample AT6 (zone 1 indicates area represented in e–h); SEM image in BSE (e, g) and SE (f, h) modes of sample AT6 zone 1; i: X-ray diffractogram of sample T6.

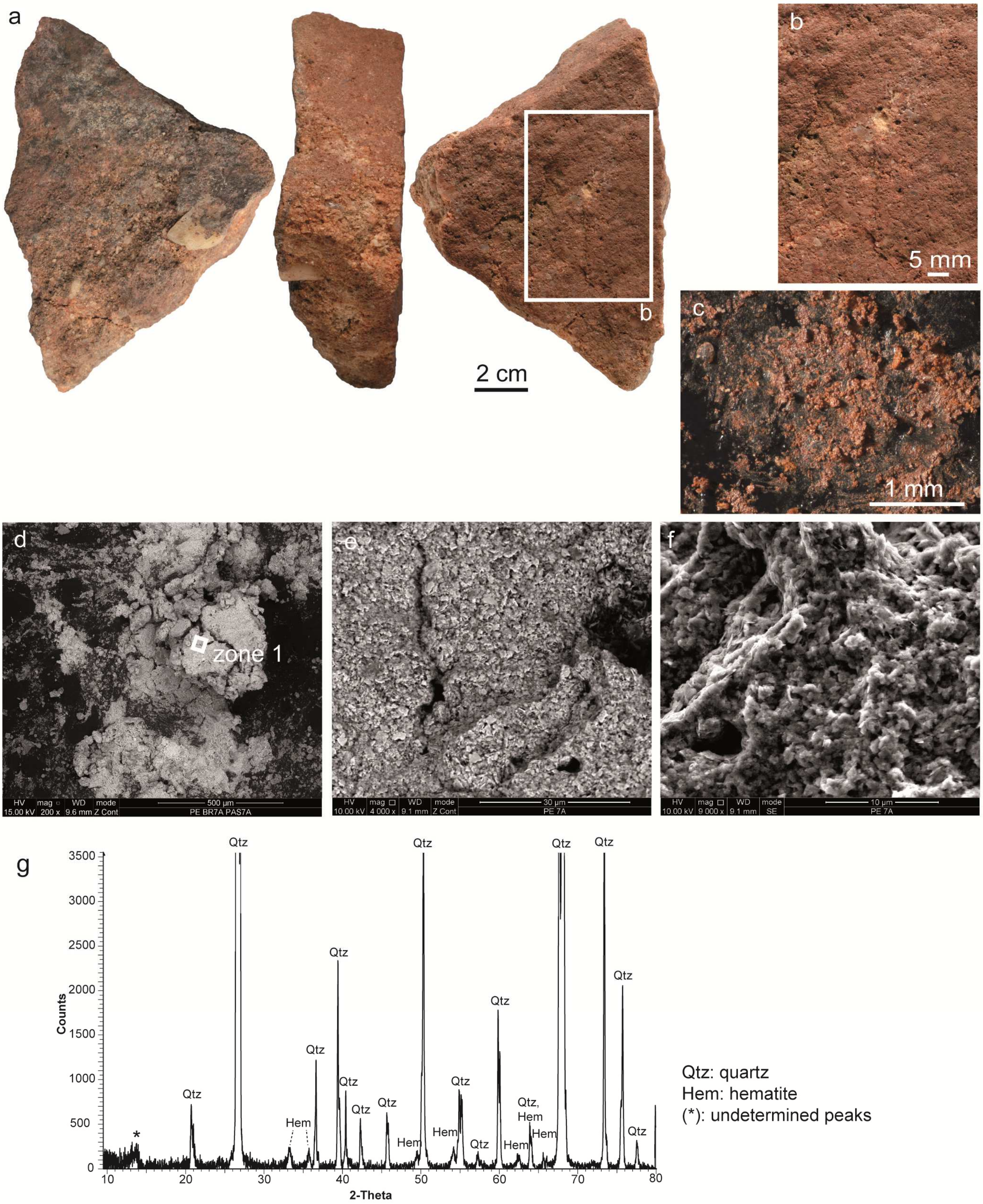

Fig G. Results of analyses conducted on ochre processing tool 7 (lower grindstone). a: Photo of the object. Square indicates the location of macro photo b; b: macro photo of smoothed area associated with red residues; c: photo of the sampled ochre residue (sample AT7); d: SEM image in BSE mode of sample AT7 (zone 1 indicates area represented in e, f); SEM images in BSE (e) and SE (f) modes of sample AT7 zone 1; j: X-ray diffractogram of sample T7.

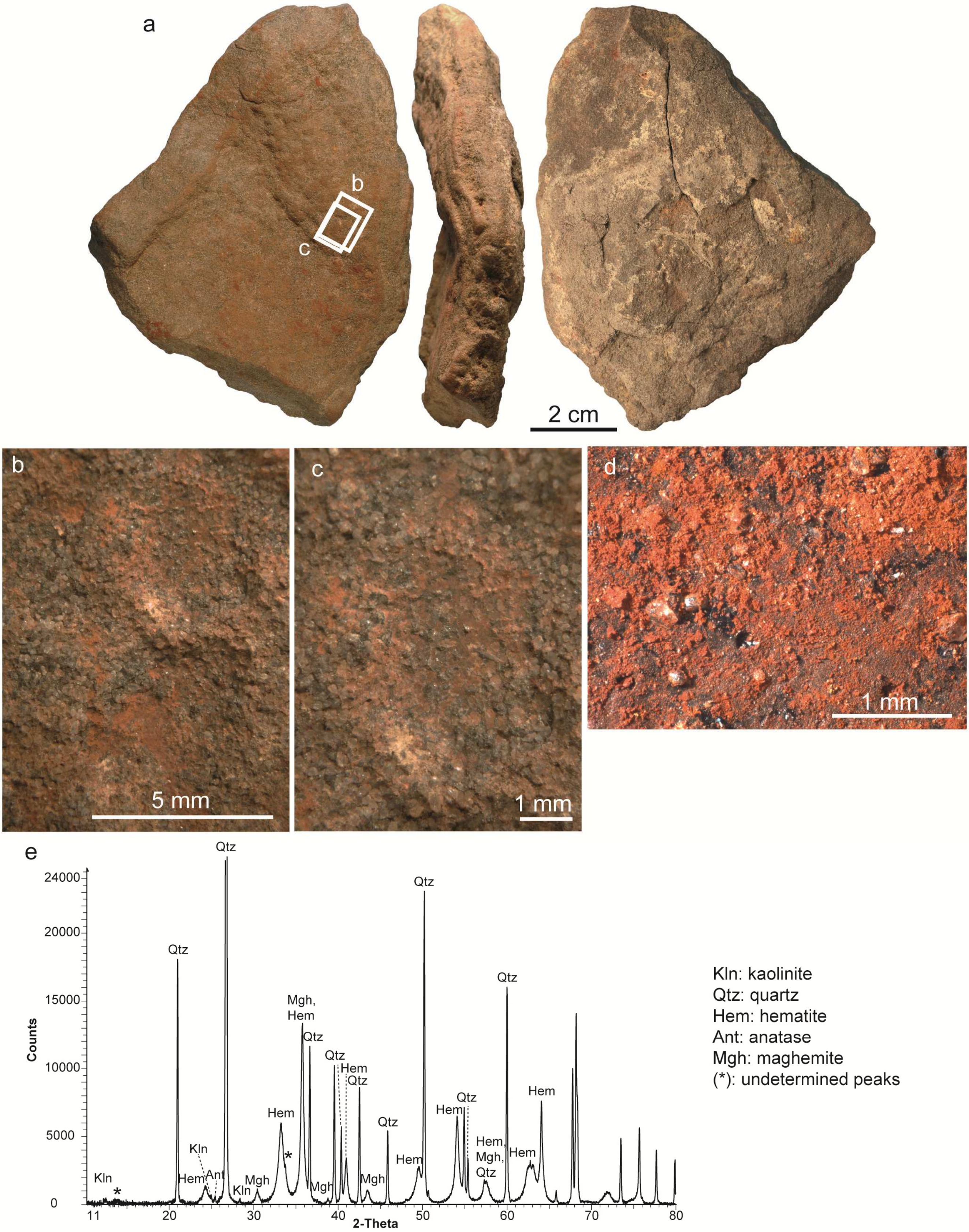

Fig H. Results of analyses conducted on ochre processing tool 8 (lower grindstone).
a: Photo of the object. Squares indicate the location of macro photos b and c; b, c: macro photos of smoothed area associated with red residues; d: photo of the sampled ochre residue (sample AT8); e: X-ray diffractogram of sample T8.

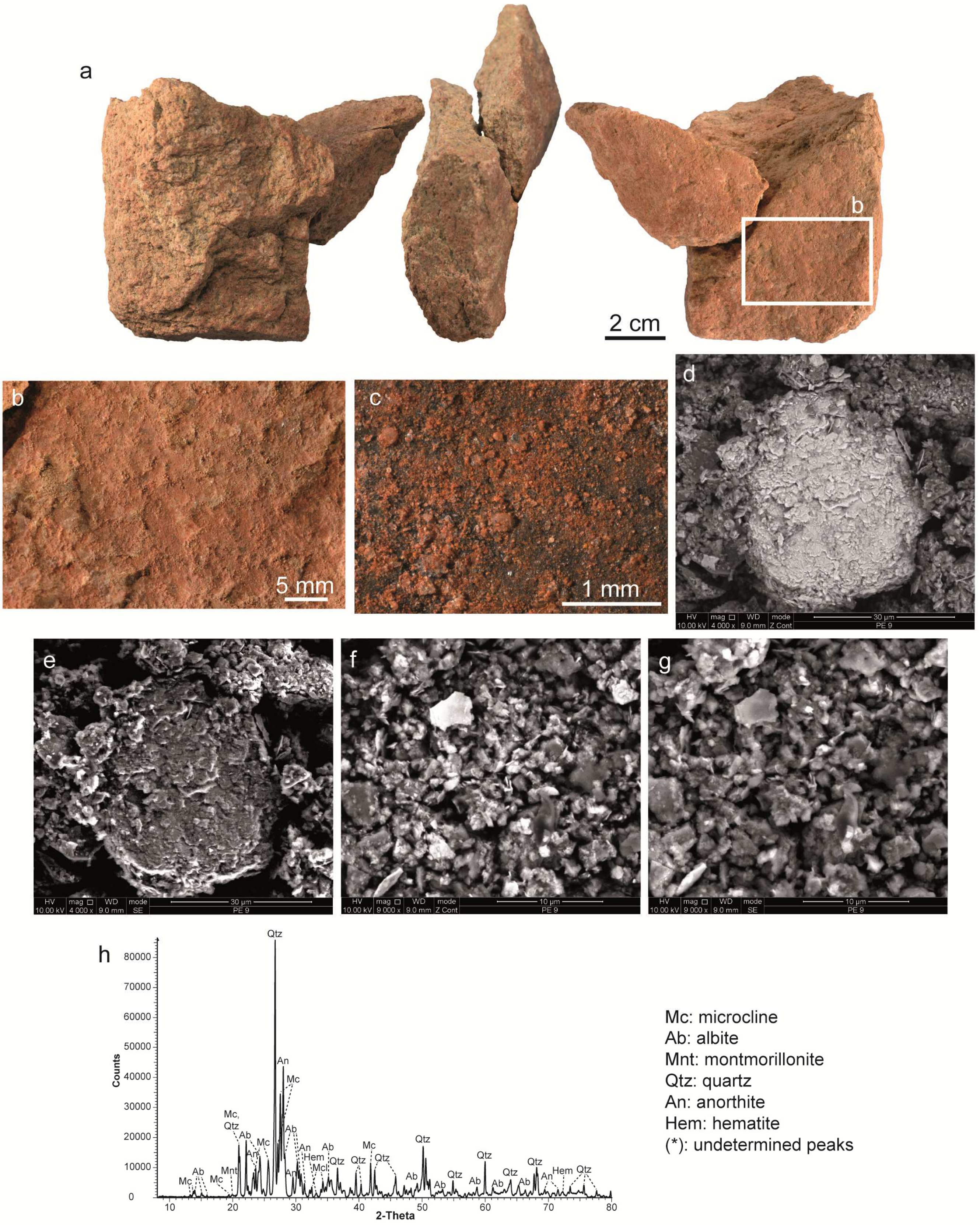

Fig I. Results of analyses conducted on ochre processing tool 9 (lower grindstone). a: Photo of the object. Square indicates the location of macro photo b; b: macro photo of smoothed areas associated with red residues; c: photo of the sampled ochre residue (sample AT9); SEM images in BSE (d) and SE (e) modes of sample AT9 zone 1; SEM images in BSE (f) and SE (g) modes of sample AT9 zone 2; h: X-ray diffractogram of sample T9.

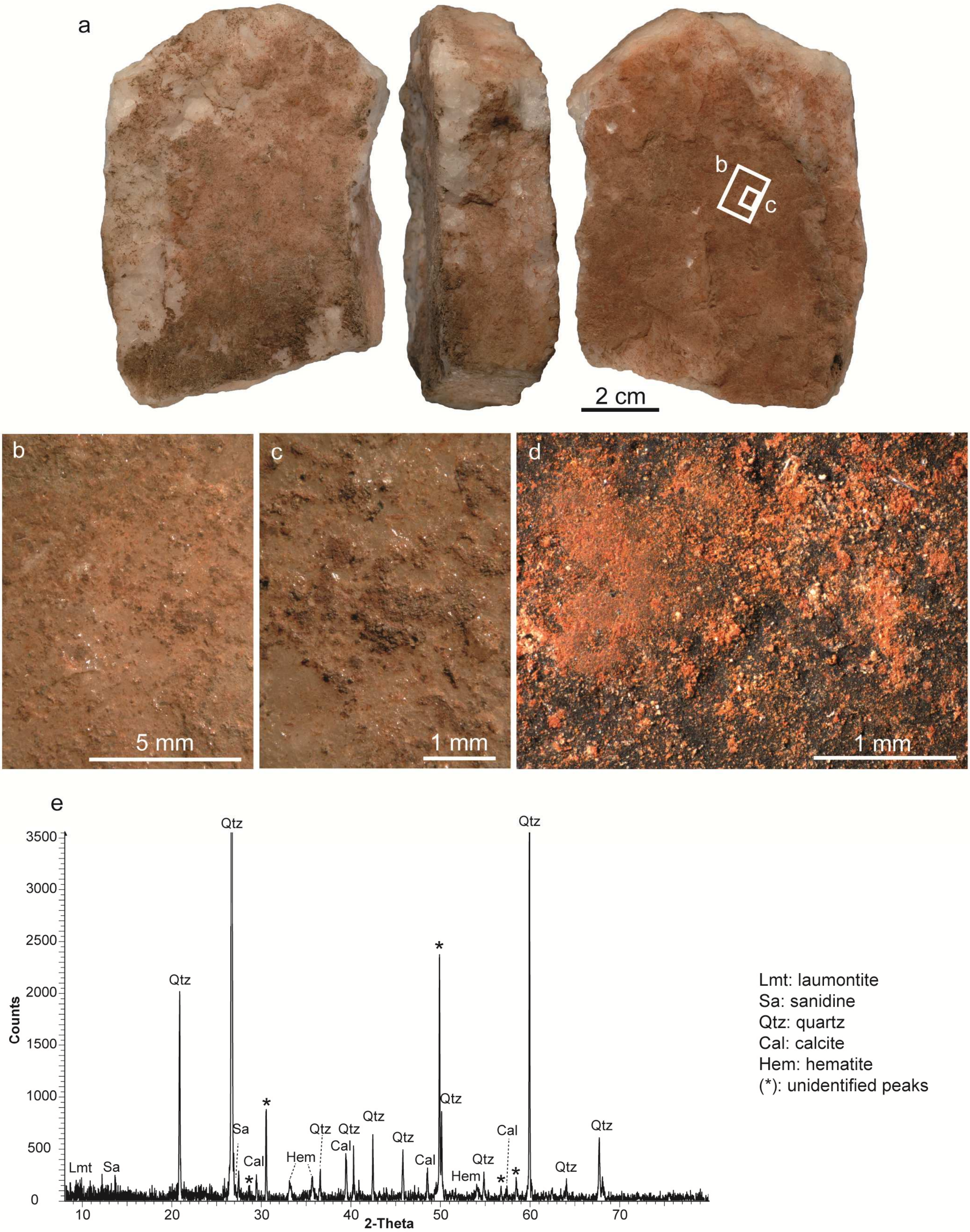

Fig J. Results of analyses conducted on ochre processing tool 10 (lower grindstone).

a: Photo of the object. Squares indicate the location of macro photos b and c; b, c: macro photos of smoothed area associated with red residues; d: photo of the sampled ochre residue (sample AT10); e: X-ray diffractogram of sample T10.

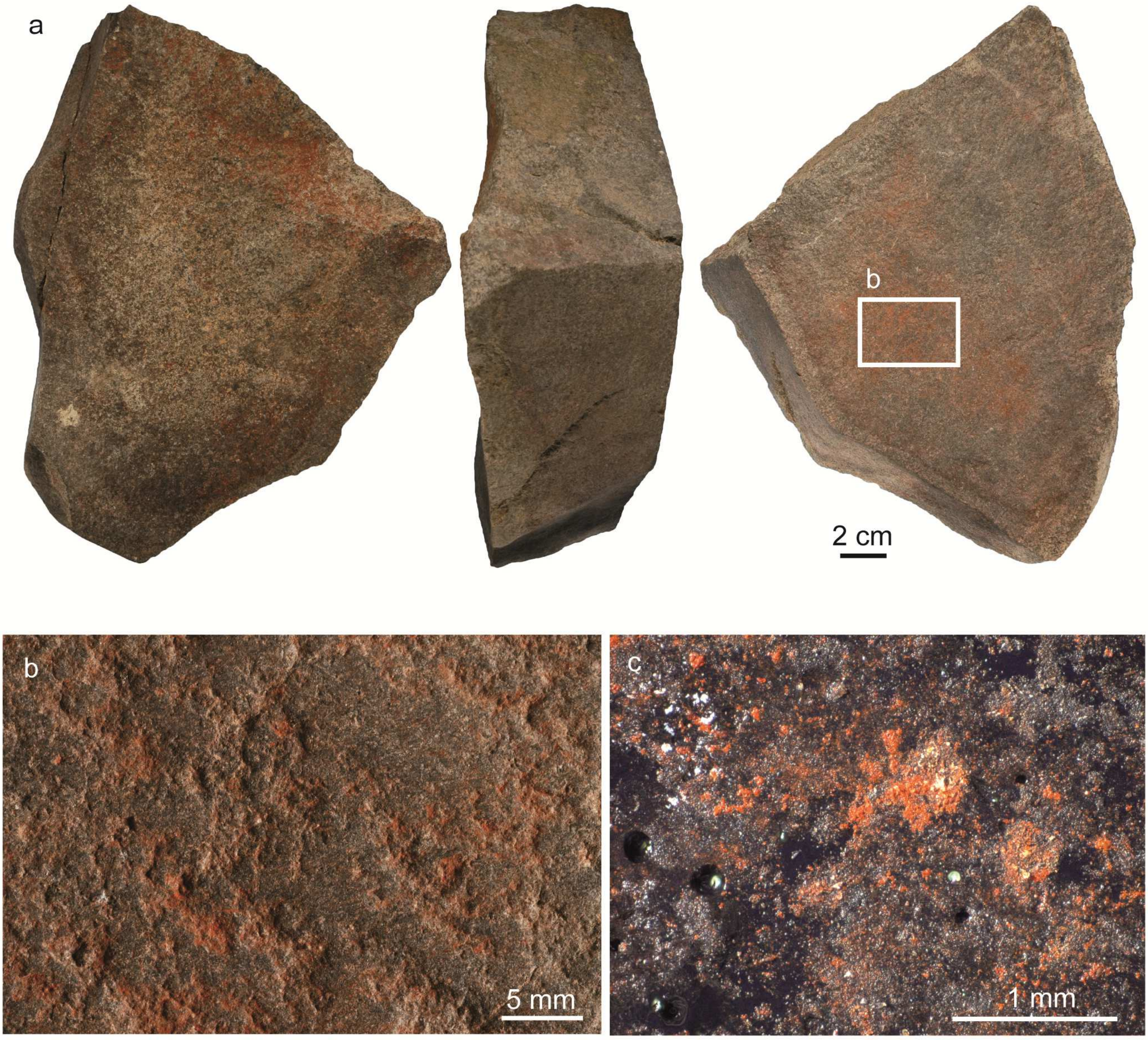

Fig K. Results of analyses conducted on ochre processing tool 11 (lower grindstone).
a: Photo of the object. Square indicates the location of macro photo b; b: macro photo of smoothed areas associated with microstriations and red residues; c: photo of the sampled ochre residue (sample AT11).
