# Supplementary material for: Middle Stone Age Ochre Processing and Behavioural Complexity in the Horn of Africa: Evidence from Porc-Epic Cave, Dire Dawa, Ethiopia
Source: PLoS One. 2016 Nov 2;11(11):e0164793. doi: 10.1371/journal.pone.0164793 (PMC5091854; doi:10.1371/journal.pone.0164793)
Supplement: S2 Fig — Photos of the artefacts, modification marks and residues, SEM-EDS images and XRD diffractograms. The objects' identification number is the same as presented in Figs 4–11, Tables 1–5, S1 Fig, S1 Table, S1 Text. (PDF) [file pone.0164793.s002.pdf]

Middle Stone Age Ochre Processing and Behavioural Complexity in the Horn of Africa: Evidence from Porc-Epic Cave, Dire Dawa, Ethiopia

Daniela Eugenia Rosso*, Africa Pitarch Martí, Francesco d'Errico

* Corresponding author
E-mail: d.rosso@pacea.u-bordeaux1.fr (DR)

S2 Figures. Results of analyses conducted on ochre processing tools 12–14; 16–23 and ochre-stained artefact 15.

Photos of the artefacts, modification marks and residues, SEM-EDS images and XRD diffractograms. The objects' identification number is the same as presented in Figs 4–11, Tables 1–5, S1 Figs, S1 Tables, S1 Texts.

Figure A. Results of analyses conducted on ochre processing tool 12 2
Figure B. Results of analyses conducted on ochre processing tool 13 3
Figure C. Results of analyses conducted on ochre processing tool 14 4
Figure D. Results of analyses conducted on ochre-stained artefact 15 5
Figure E. Results of analyses conducted on ochre processing tool 16 6
Figure F. Results of analyses conducted on ochre processing tool 17 7
Figure G. Results of analyses conducted on ochre processing tool 18..........................8
Figure H. Results of analyses conducted on ochre processing tool 19..........................9
Figure I. Results of analyses conducted on ochre processing tool 20..........................10
Figure J. Results of analyses conducted on ochre processing tool 21.........................11
Figure K. Results of analyses conducted on ochre processing tool 22 12
Figure L. Results of analyses conducted on ochre processing tool 23.........................13

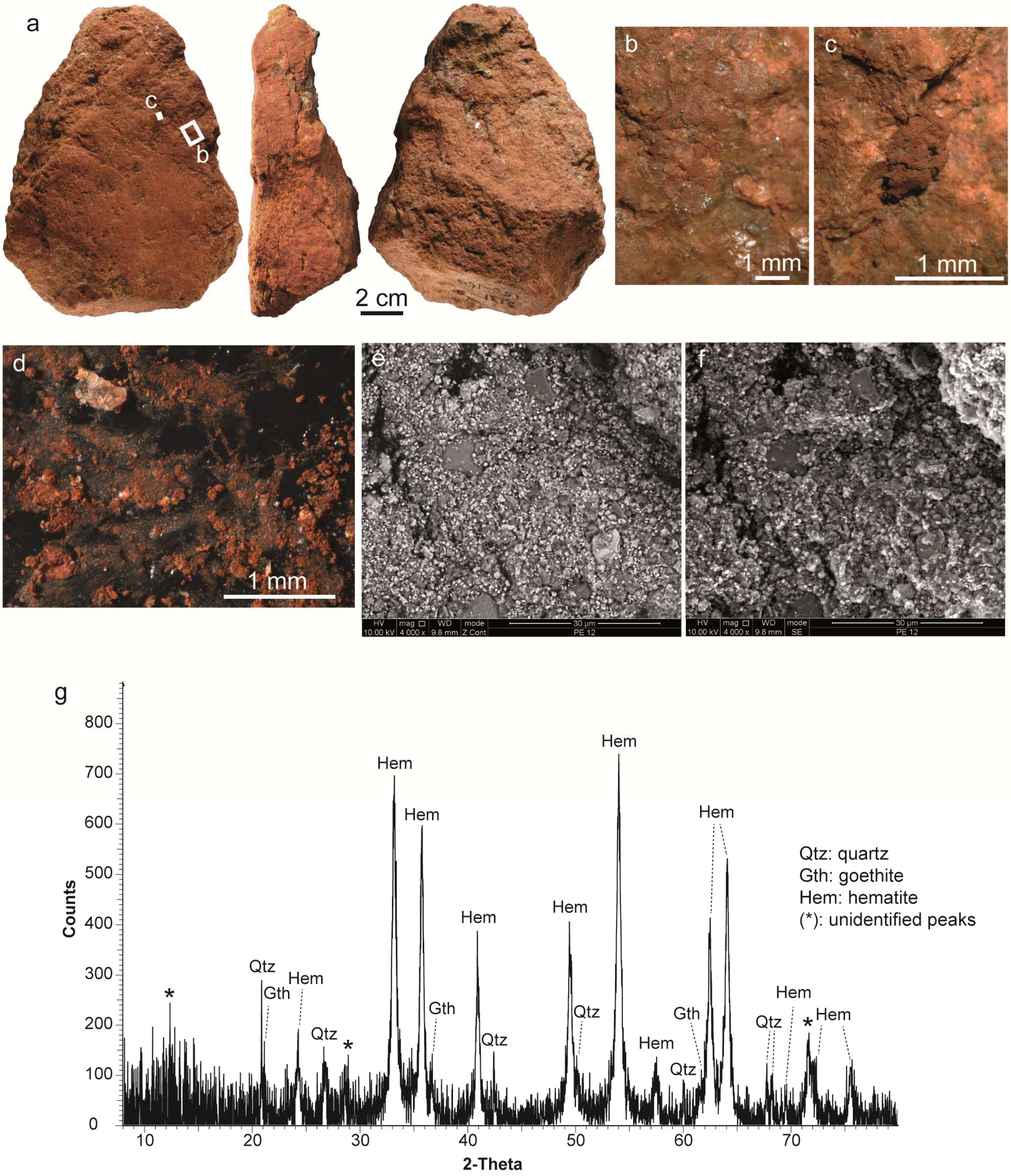

Fig A. Results of analyses conducted on ochre processing tool 12 (lower grindstone). a: Photo of the object. Squares indicate the location of macro photos b and c; b, c: macro photos of smoothed areas associated with red residues; d: photo of the sampled ochre residue (sample AT11); SEM images in BSE (e) and SE (f) modes of sample AT12; g: X-ray diffractogram of sample T12.

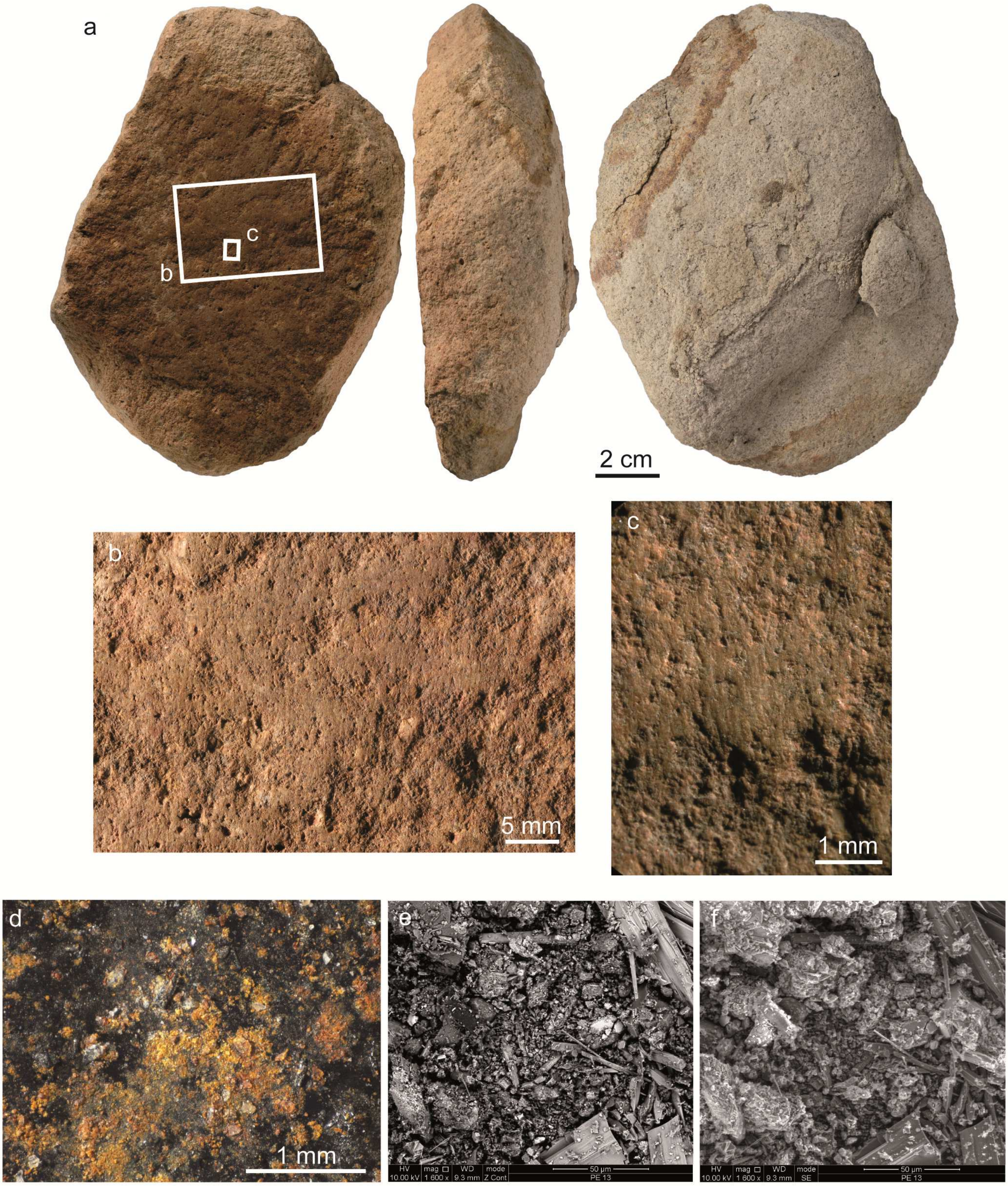

Fig B. Results of analyses conducted on ochre processing tool 13 (lower grindstone). a: Photo of the object. Squares indicate the position of macro photos b and c; b, c: macro photos of smoothed areas associated with microstriations and red residues; d: photo of the sampled ochre residue (sample AT13); SEM images in BSE (e) and SE (f) modes of sample AT13.

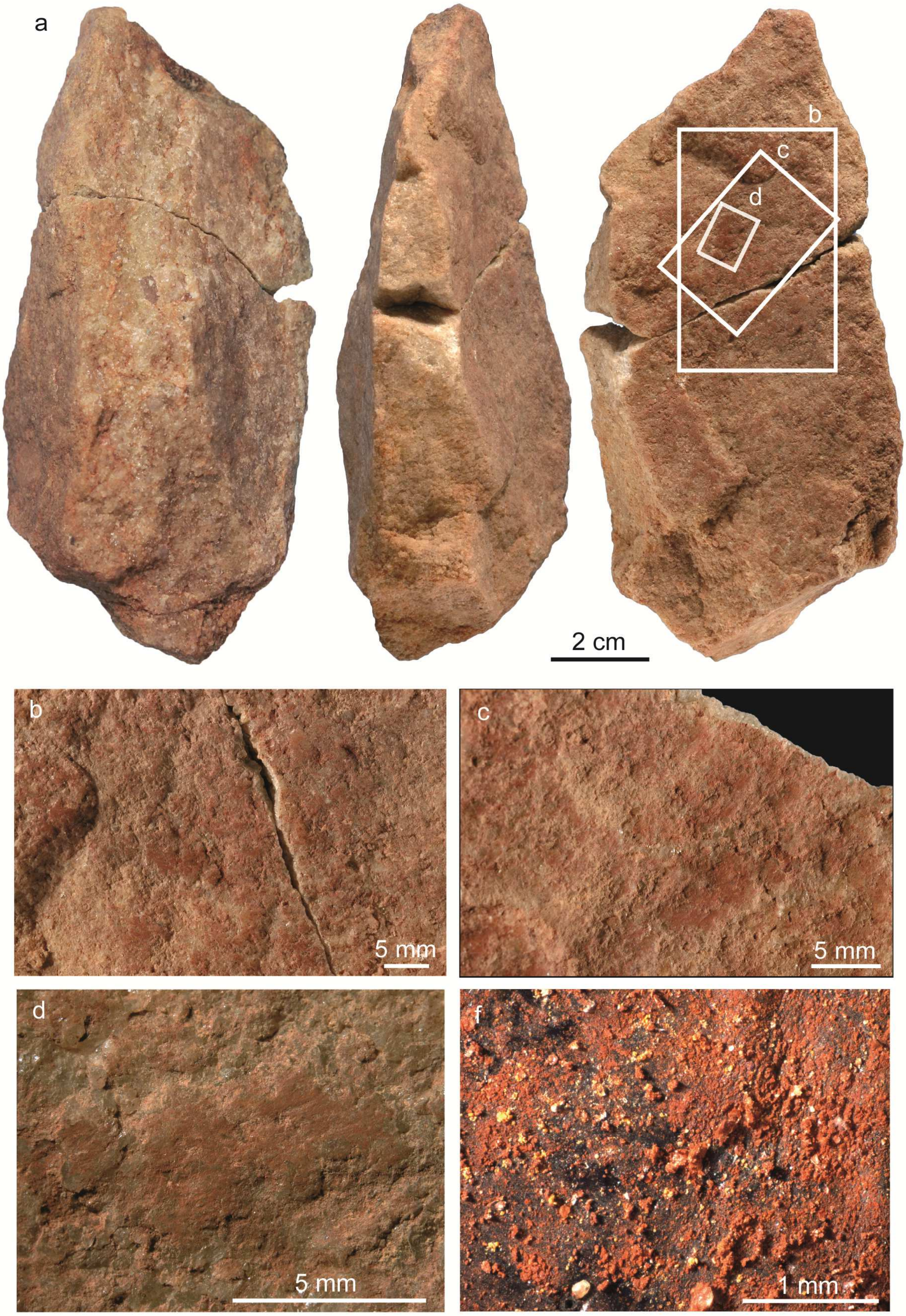

Fig C. Results of analyses conducted on ochre processing tool 14 (lower grindstone). a: Photo of the object. Squares indicate the position of macro photos b–d; b–d: macro photos of smoothed areas associated with microstriations and red residues; f: photo of the sampled ochre residue (sample AT14).

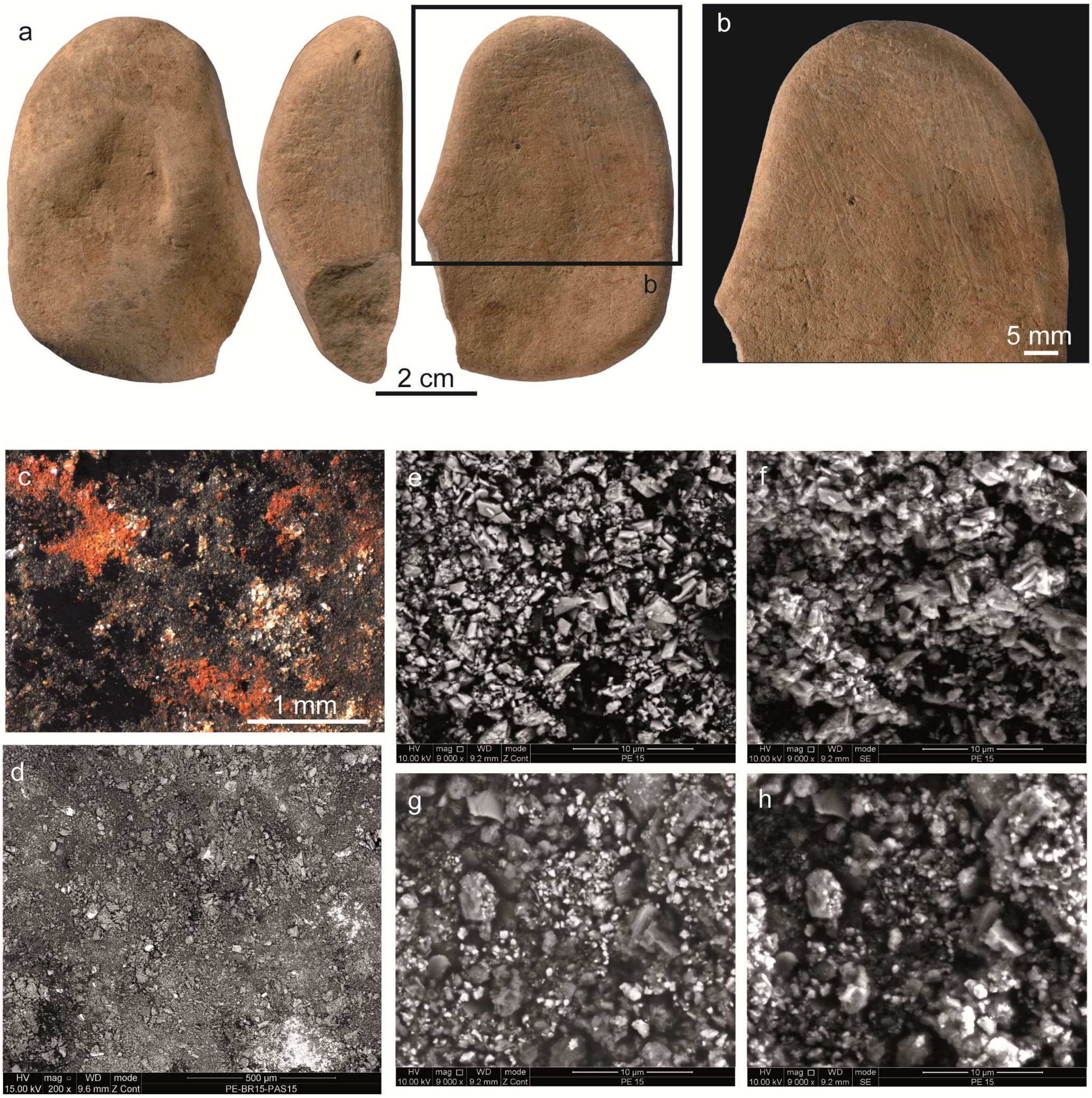

Fig D. Results of analyses conducted on ochre-stained artefact 15.
a: Photo of the object. Square indicates the position of macro photo b; b: macro photo of linear impressions associated with striations and red residues; c: photo of the sampled ochre residue (sample AT15); d: SEM image in BSE mode of sample AT15; SEM images in BSE (e) and SE (f) modes of sample AT15 zone 1; SEM images in BSE (g) and SE (h) modes of sample AT15 zone 2.

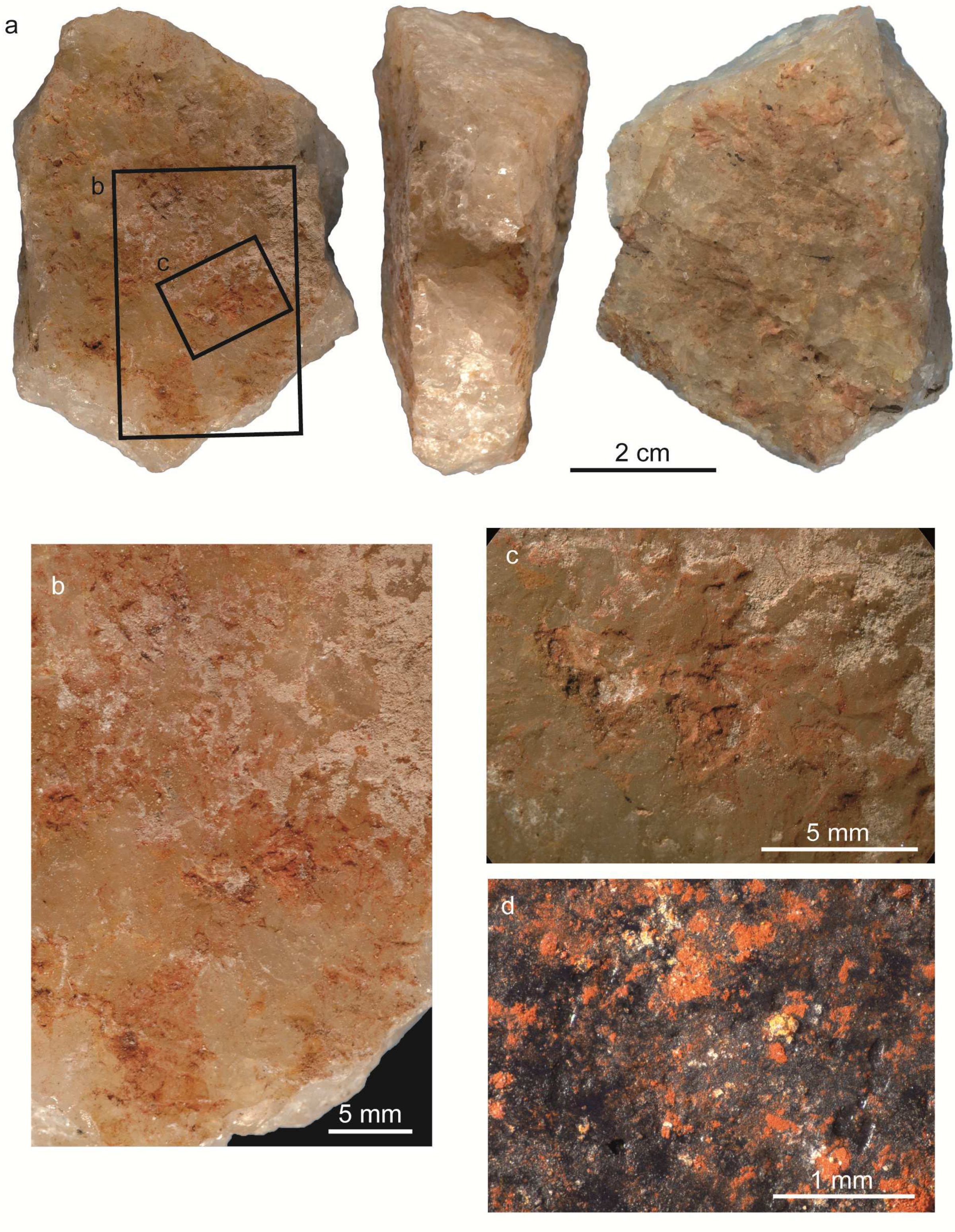

Fig E. Results of analyses conducted on ochre processing tool 16 (lower grindstone). a: Photo of the object. Squares indicate the location of macro photos b and c; b, c: macro photos of red residues; d: photo of the sampled ochre residue (sample AT16).

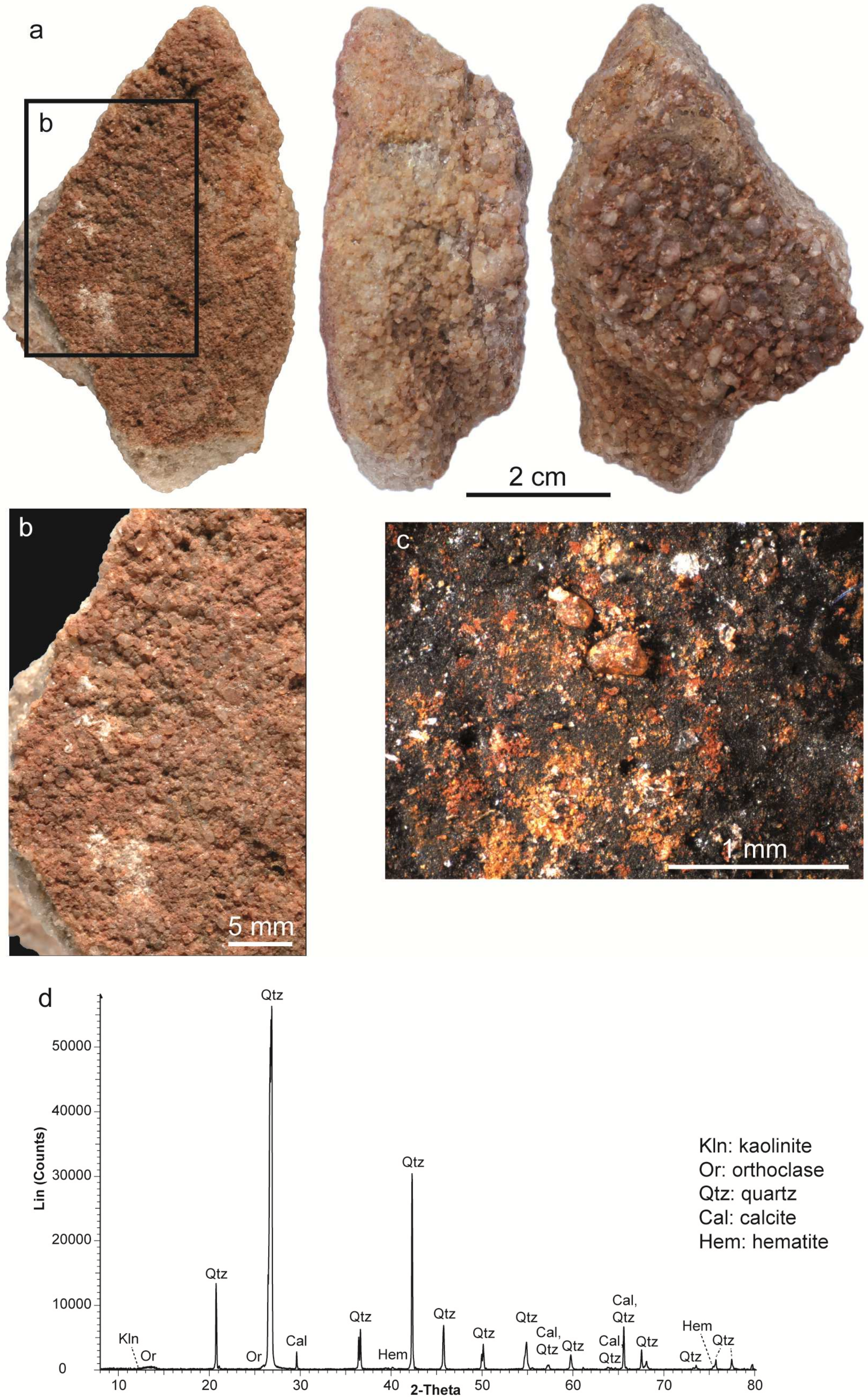

Fig F. Results of analyses conducted on ochre processing tool 17 (lower grindstone). a: Photo of the object. Square indicates the position of macro photo b; b: macro photo of red residues; c: photo of the sampled ochre residue (sample AT17); d: X-ray diffractogram of sample T17.

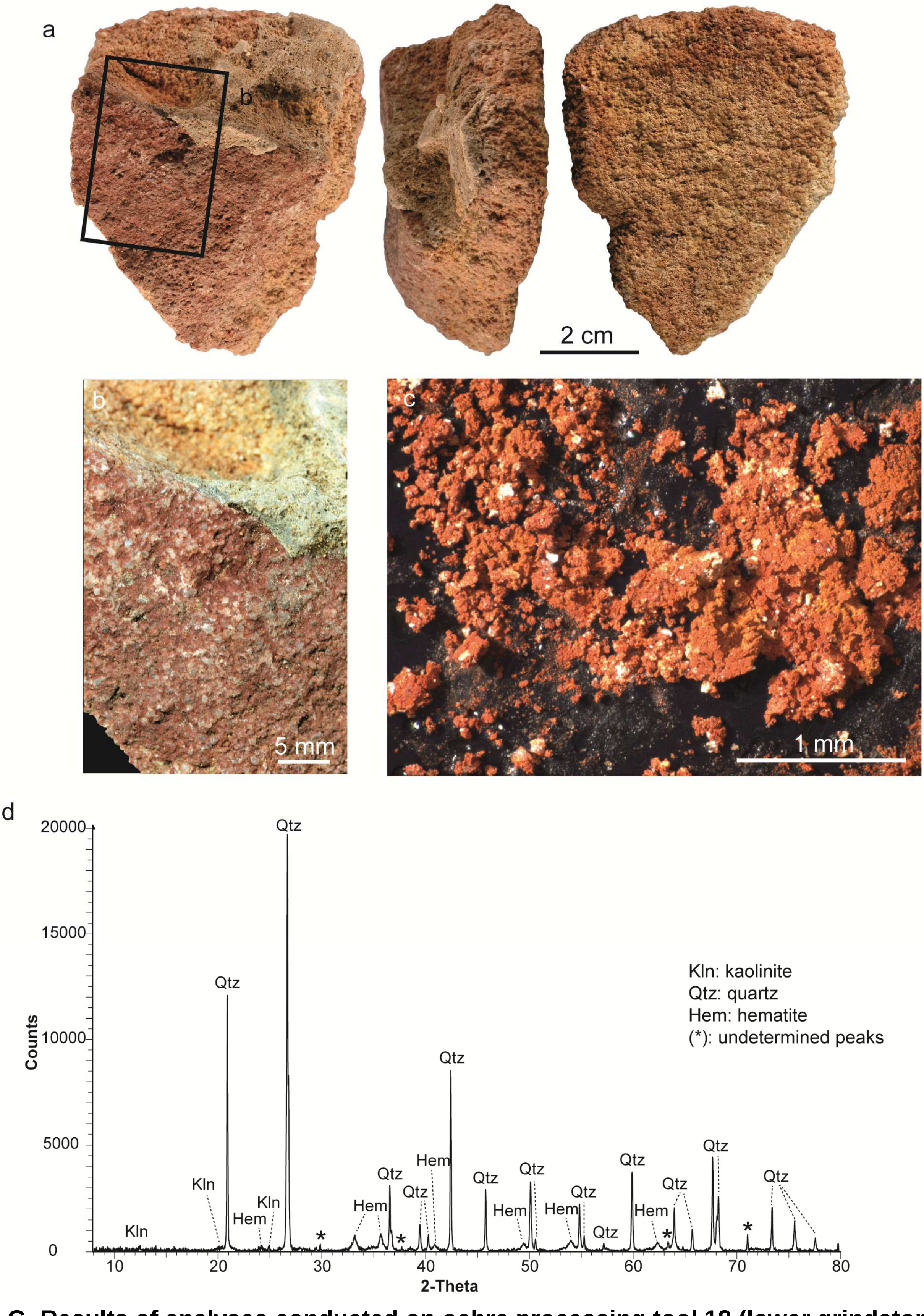

Fig G. Results of analyses conducted on ochre processing tool 18 (lower grindstone). a: Photo of the object. Square indicates the position of macro photo b; b: macro photo of red residues; c: photo of the sampled ochre residue (sample AT18); d: X-ray diffractogram of sample T18.

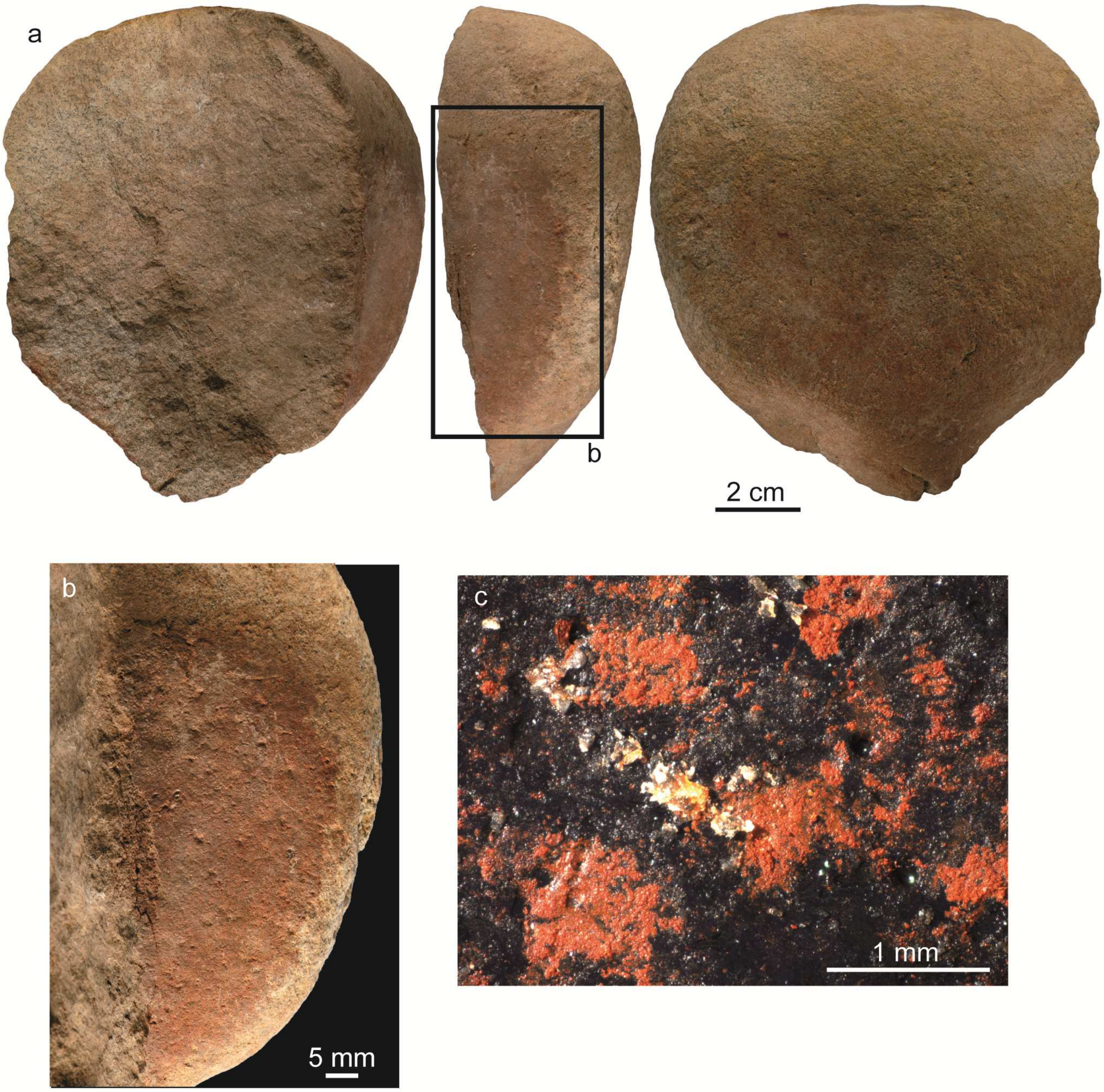

Fig H. Results of analyses conducted on ochre processing tool 19 (upper grindstone).
a: Photo of the object. Square indicates the position of macro photo b; b: macro photo of pits associated with red residues; c: photo of the sampled ochre residue (sample AT19).

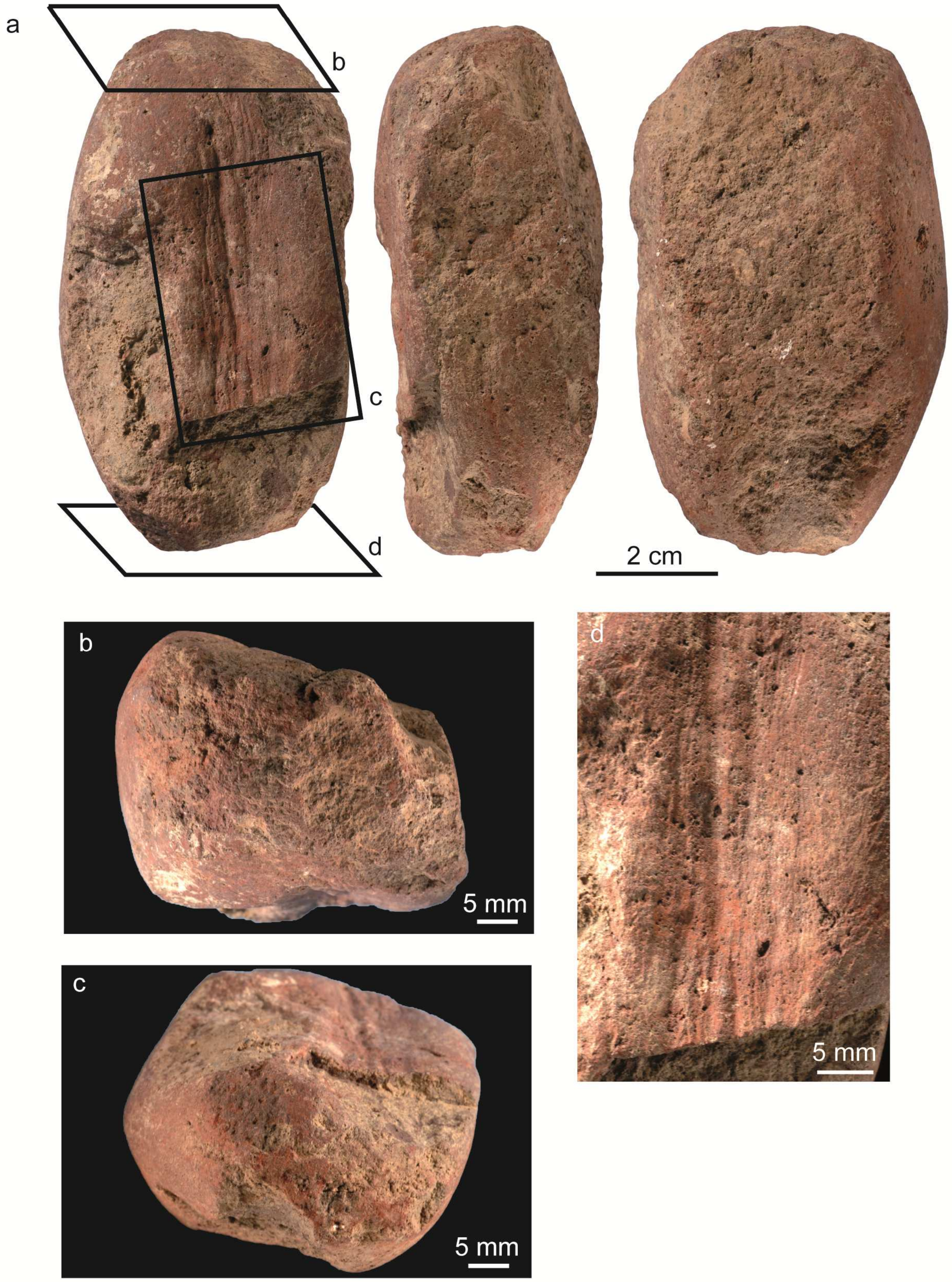

Fig I. Results of analyses conducted on ochre processing tool 20 (upper grindstone). a: Photo of the object. Squares indicate the location of macro photos (b–d); macro photos of pits associated to smoothed areas (b, c), flake scar (c), and deep grooves (d).

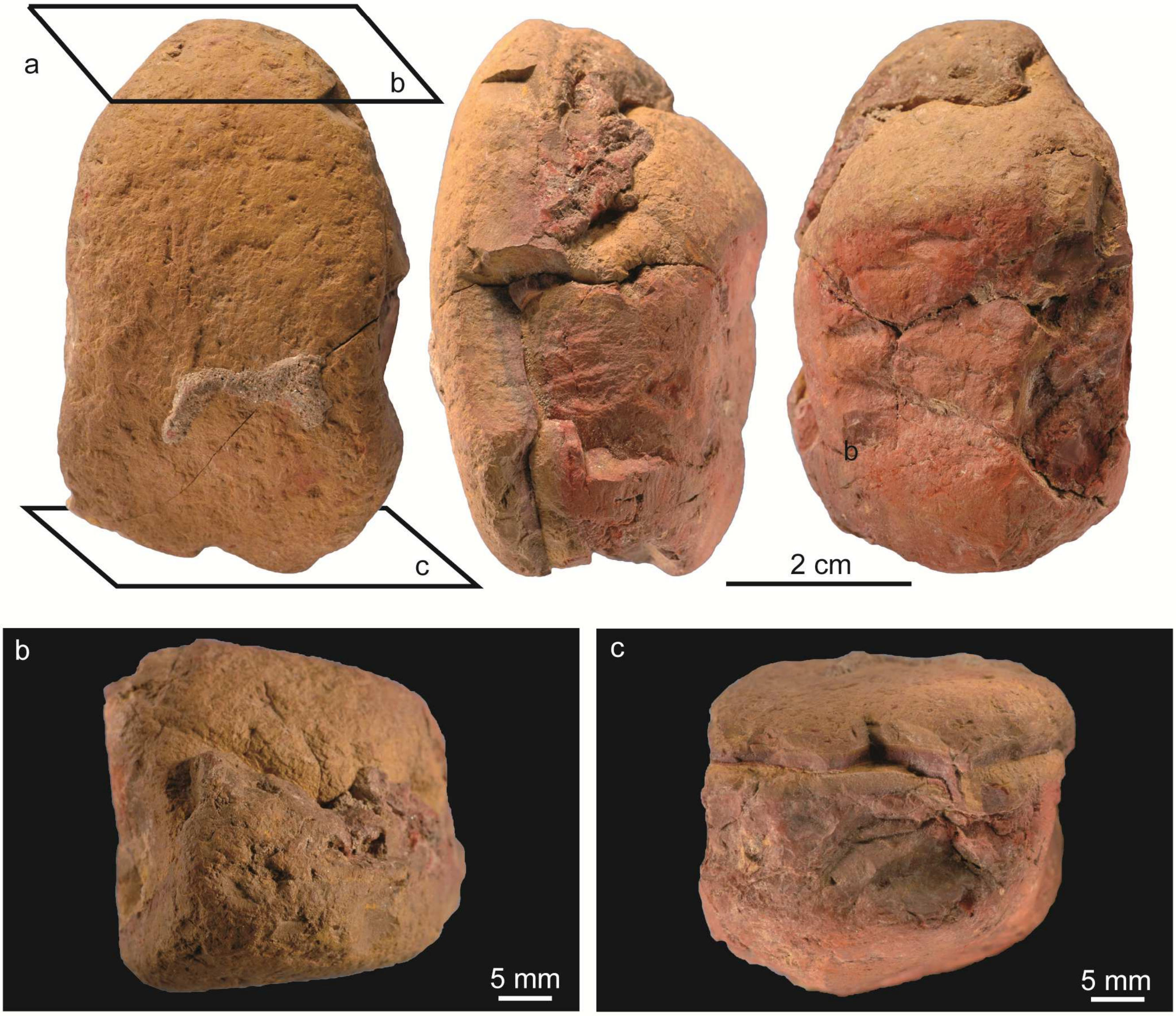

Fig J. Results of analyses conducted on ochre processing tool 21 (upper grindstone). a: Photo of the object. Squares indicate the location of photos b and c; photos of pits (b, c) and flake scars (c).

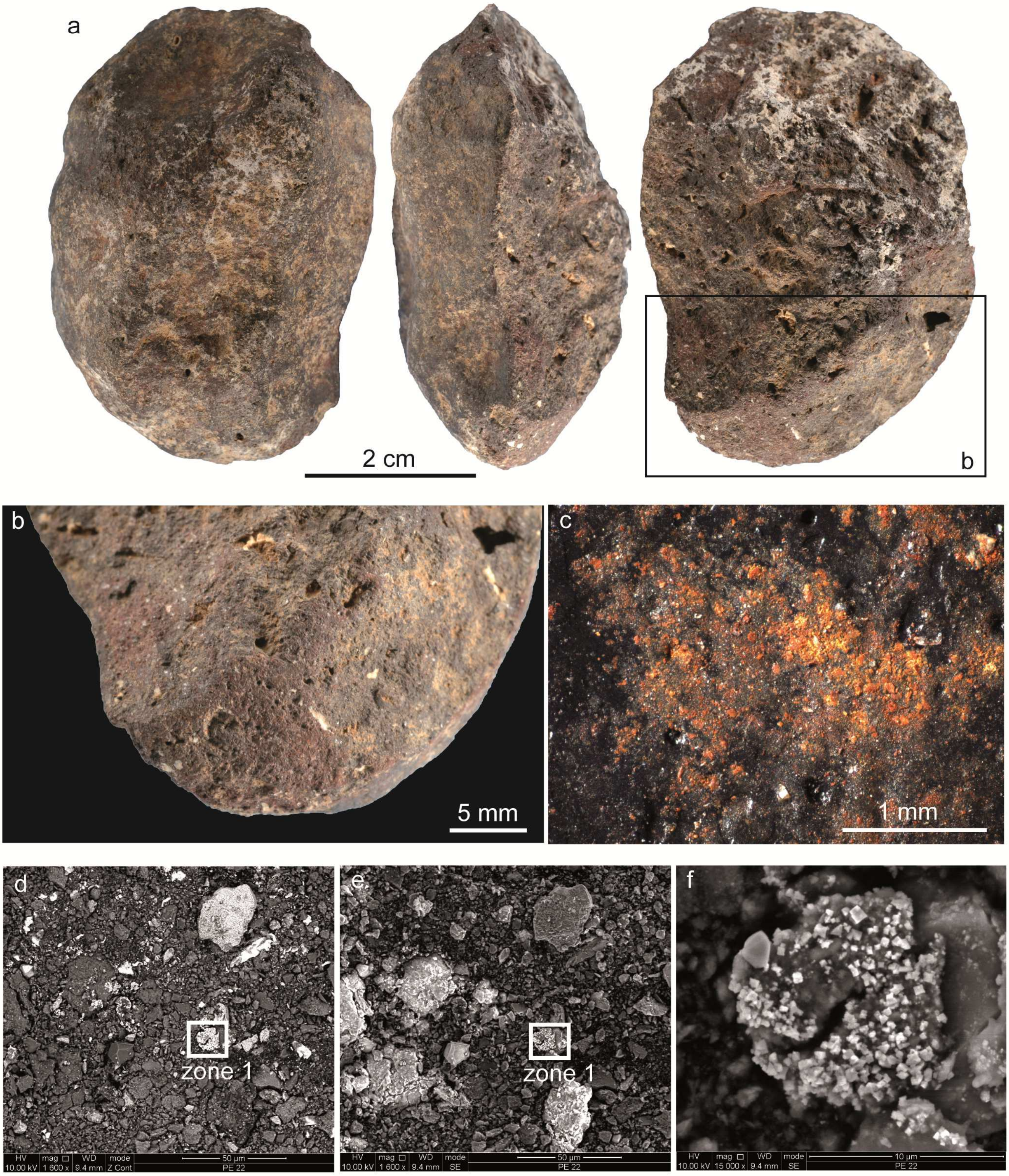

Fig K. Results of analyses conducted on ochre processing tool 22 (upper grindstone).

a: Photo of the object. Square indicates the location of macro photo b; b: macro photo of pits; c: photo of the sampled ochre residue (sample AT22); SEM images in BSE (d) and SE (e) modes of sample AT22 (zone 1 indicates the area represented in c); SEM image in SE mode of sample AT22 zone 1.

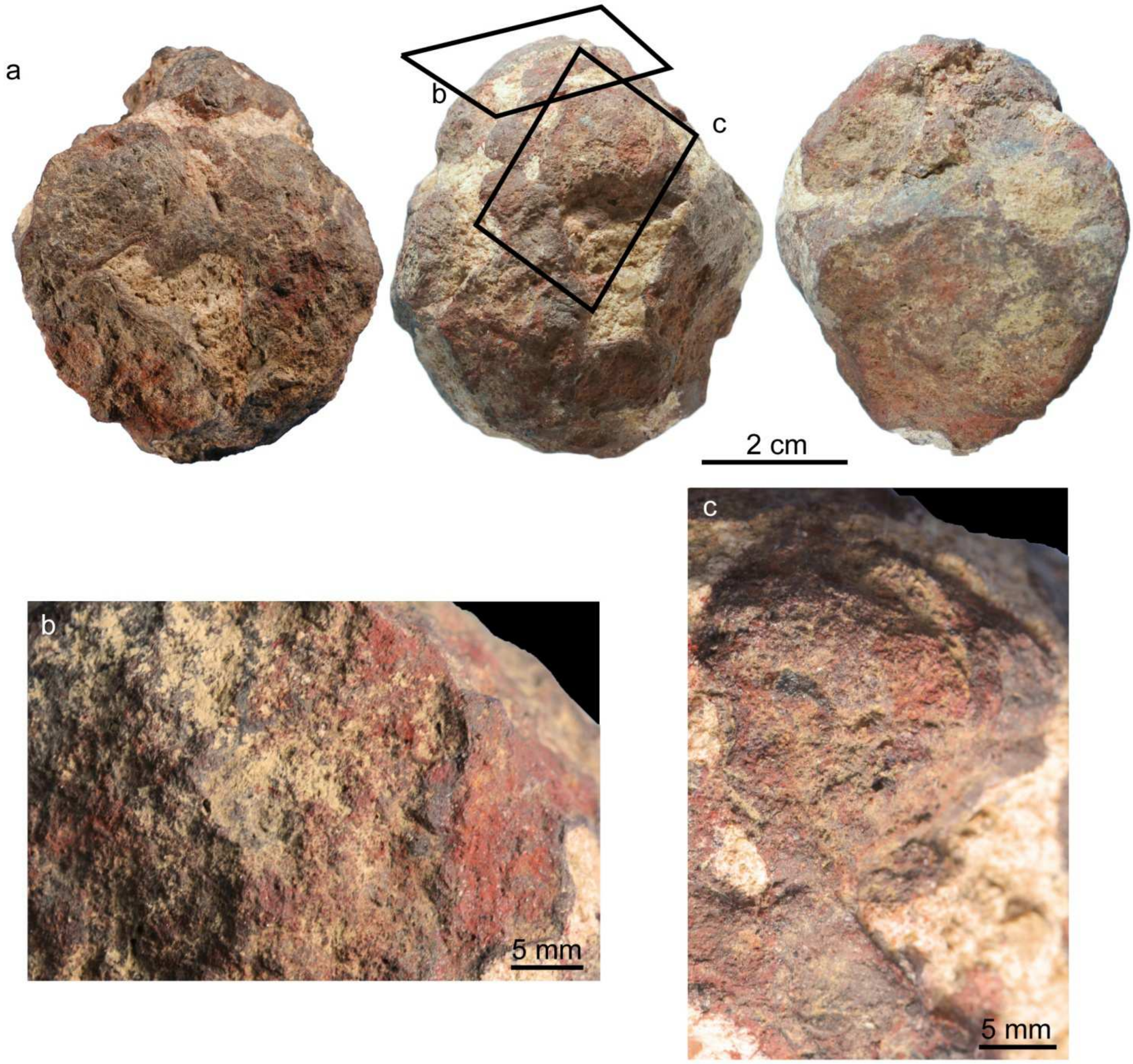

Fig L. Results of analyses conducted on ochre processing tool 23 (upper grindstone). a: Photo of the object. Squares indicate the position of macro photos b and c; b, c: macro photos of pits.
