# Supplementary material for: Middle Stone Age Ochre Processing and Behavioural Complexity in the Horn of Africa: Evidence from Porc-Epic Cave, Dire Dawa, Ethiopia
Source: PLoS One. 2016 Nov 2;11(11):e0164793. doi: 10.1371/journal.pone.0164793 (PMC5091854; doi:10.1371/journal.pone.0164793)
Supplement: S1 Table — SEM-EDS and μ-RS analyses. The objects' identification number is the same as presented in Figs 4–11, Tables 1–5, S1 and S2 Figs, S1 Text. (PDF) [file pone.0164793.s003.pdf]

Middle Stone Age Ochre Processing and Behavioural Complexity in the Horn of Africa: Evidence from Porc-Epic Cave, Dire Dawa, Ethiopia

Daniela Eugenia Rosso*, Africa Pitarch Martí, Francesco d'Errico

* Corresponding author
E-mail: d.rosso@pacea.u-bordeaux1.fr (DR)

S1 Tables. Detailed results of elemental and mineralogical analyses conducted on ochre processing tools and ochre-stained artefacts.

SEM-EDS and μ-RS analyses. The objects' identification number is the same as presented in Figs 4–11, Tables 1–5, S1 and S2 Figs, S1 Texts.

Table A. Results of SEM-EDS analyses......2
Table B. Results of μ-RS analyses......4

Table A. Results of SEM-EDS analyses.

Num	Sam	Num of an	Description of analysed item				Semi-quantitative EDX analyses **				Interpretation***
			Grain morph	BSE cont*	Length range (µm)	Width range (µm)	>10%	10-3%	3-1%	<1%	
1	AT1A	1	Irreg	W	39	26	**Fe**	Mn, (Si)	(Ca, Al, K)	(Cl, Mg, Ti, Na, P)	Iron ox (+ clay min)
1	AT1A	4	Aggl	W	Submicr	Submicr	**Fe**	(Si, Al)	(Ca, K)	(Mg), Mn, (Ti, P, Cl, Na)	Iron ox (+ clay min)
1	AT1A	2	Plat	G	34	26	Si, (Fe)	Al, K, Na	(Ca)	(Mg)	K-rich mica (+ iron ox)
1	AT1A	1	Ang	G	30	19	Ca, Mg	(Fe, Si)	(P, Al, K)	(Mn)	Carbonate (+ clay min)
1	AT1A	1	Irreg	W	24	10	**Ba**	S, (Si, Fe)	(Al, Ca, K)	(Mg, Na, Mn, P)	Ba-rich Sulphate (+ clay min + iron ox)
1	*AT1A*	*3*	-	*W*	-	-	***Fe**, Si*	*Al, Ca, K*	-	*Mg, (Cl), Ti, P, Mn, Na, Cr*	*Mixture of iron ox and aluminosil*
1	*AT1A*	*2*	-	*G*	-	-	*Fe, Si*	*Al, Ca, K*	*Mg*	*P, Ti, (Cl) ,Na, S*	*Mixture of iron ox and aluminosil*
2	AT2A	2	Irreg	W	21-34	13	**Fe**, Cr	(Si), Ni, (Al)	(Ca, Cl)	(K, Mg, P, S, Na, Ti)	Undet
2	AT2A	1	Ang	LG	42	42	Si, (Fe)	(Ca, Al, Mg)	(K)	(Cl, Na, Ti, S, P)	Silicate (+ Ca-rich aluminosil)
2	AT2A	1	Irreg	G	62	35	(Fe), Si	Al, Ca, K	(Cl, S), Na, (Ti)	Mg, P	Ca-rich feldspar (+ iron ox)
2	*AT2A*	*3*	-	*G*	-	-	*Fe, Si*	*Al, Ca*	*K, (Cl), S*	*Mg, Ti, P, Na*	*Mixture of iron ox and clay min*
2	*AT2A*	*1*	-	*W*	-	-	*Fe, (Si)*	*(Cl, Al, Ca, K)*	*(Mg)*	*(S, Na), Ti, (P)*	*Iron ox (+ clay min)*
2	AT2B	1	Aggl	W	Submicr	Submicr	**Fe**, (Si)	(Ca, Al, Cl)	(K)	(Mg), Ti, (Na, P)	Iron ox (+ Ca-rich feldspars)
2	AT2B	2	Ang	G	96-156	54-90	Si, (Fe)	(Al, Ca, Mg)	(Cl, K)	(Na)	Silicate (+ aluminosil)
2	*AT2B*	*3*	-	*W*	-	-	*Fe, Si*	*Ca, Al, (Cl)*	*Mg, K, Na*	*Ti, P, S*	*Mixture of iron ox and clay min*
3	AT3	2	Subcirc	W	15-17	12	**Fe**	(Si)	(Al)	(Cl, Ca, S, K, Mg, P)	Iron ox (+ silicates + clay min)
3	AT3	2	Aggl	W	Submicr	Submicr	**Fe**	(Si, Al)	-	(Ti, Cl, Ca, S, P, K, Mg)	Iron ox (+ silicates + Ti-rich clay min)
3	AT3	2	Irreg	W	18-19	12-14	**Fe**, Cr	Ni, (Si)	(Al)	(Mn, Ca, Cl, Ti, S, K)	Undet
3	AT3	2	Irreg	G	522-807	413-531	**Si**	(Fe)	(Al)	(Ca, Cl, Ti)	Silicate (+ iron ox + Ti-rich clay min)
3	AT3	1	Ang	G	5-10	5-10	**Si**	(Fe)	(Al)	-	Silicate (+ iron ox)
3	*AT3*	*3*	-	*G*	-	-	*Si, Fe*	*Al, (Cl), Ca*	-	*K, Ti, Mg, P, (Cr), S*	*Mixture of iron ox and Ti-rich clay min*
4	AT4	4	Oct	G	58-63	41-51	Fe, (Si), Ti	(Al)	(Ca, K, Mg, Na)	(P, Cl), Mn, (S)	Undet ox (+ silicates + clay min)
4	*AT4*	*4*	-	*G*	-	-	*Fe, Si*	*Al*	*Ca, K, Mg, Na, Ti*	*(Cl), P*	*Mixture of iron ox and aluminosil*
5	AT5	4	Irreg	W	17-38	13-27	**Fe**, (Si)	(Al)	(Cr, K)	(Ca, Cl, Mg, Na, Ti, S, P)	Iron ox (+ feldspars + clay min)
5	AT5	1	Irreg/acic	W	1-2	1-2	Fe, (Si)	(Al, K)	(Ca, Cr, Cl)	(Mg, Na, Ti)	Iron ox (+ feldspars + clay min)
5	AT5	1	Irreg	G	24	15	Si	Al, Na, K	(Fe)	(Cl), P	Feldspar (+ clay min)
5	AT5	1	Aggl	G	Submicr	Submicr	(Fe), Si	Al, (Cr), K	Ca, (Cl)	Mg, Ti, Na	Clay min (+ iron ox)
5	AT5	1	Irreg	G	30	25	Si, (Fe)	(Al)	(K, Ca)	(Cl, Mg, S, Na)	Silicate (+ clay min)
5	*AT5*	*3*	-	*G*	-	-	*Si, Fe*	*Al*	*K, Ca, (Cl)*	*Mg, S, Ti, Na*	*Mixture of iron ox and aluminosil*
6	AT6B	3	Irreg/plat	W	55-134	39-113	**Fe**, Ti	(Si, Al)	(Mg)	(Cr), Mn, (Ca, K, Cl, Na, P)	Undet ox (+ aluminosil)
6	AT6B	2	Aggl	G	Submicr	Submicr	Si, Al	(Fe)	Mg, K, (Cl, Ti)	Ca, Na, P, S	Clay min (+ iron ox)
6	AT6B	1	Ang	G	194	111	Si, Ca	Mg, Fe, Al	Ti	Na,(K, Cl)	Undet silicate (+ clay min)
6	*AT6B*	*3*	-	*G*	*Submicr*	*Submicr*	*Si, Fe*	*Al*	*Ca, Ti, Mg, K, (Cl)*	*Cr, Mn, Na, P*	*Mixture of iron ox and aluminosil*

7	AT7	2	Irreg	W	20-29	9-20	**Fe**	-	(Si, Al, Cl, Ca)	(Ti, K, Mg, P)	Iron ox (+ aluminosil)
7	AT7	1	Irreg	G	56	34	Si, Al	(Fe), Ca	Na, (Cl)	K, Mg, Mn, Ti	Ca, Na-rich feldspar (+ iron ox)
7	AT7	2	Aggl	W	2-4	2-4	Fe, (Si)	(Cl, Ca, Al)	-	(K, Mg), Mn, (Ti, P)	Iron ox (+ aluminosil)
7	AT7	1	Aggl	G	211	131	(Fe)	Si, Al	(Cl), Ca	Mg, K, Ti, Mn, Na, P	Undet Aluminosil (+ iron ox)
7	AT7	1	Irreg	G	326	241	Si, (Fe)	(Al)	(Cl, Ca)	(K, Mg, Ti, P)	Silicate (+ iron ox + aluminosil)
7	*AT7*	*3*	*-*	*W*	*-*	*-*	*Fe*	*Si, Al, (Cl), Ca*	*-*	*K, Mg, Ti, P, Na, S*	*Mixture of iron ox and aluminosil*
7	*AT7*	*3*	*-*	*W*	*-*	*-*	***Fe***	*(Cl, Ca, Al, Si)*	*-*	*(P, K, Mg, S, Ba, Ti, Na, Cr)*	*Iron ox (+ aluminosil)*
9	AT9	5	Aggl	W	5-10	5-10	**Fe**	(Si, Cl)	(Al, Ca)	(K, Na, Mg, P, Ti)	Iron ox (clay min)
9	AT9	2	Irreg	G	131-153	109-129	**Si**	**-**	(Al, Fe)	(Ca, Cl, K, Mg, Na)	Silicate (+ iron ox + clay min)
9	AT9	3	Irreg	G	27-194	21-150	Si, K, Al	(Fe)	**-**	Na, Mg, (P)	Feldspar (+ iron ox + clay min)
9	*AT9*	*1*	*-*	*G*	*-*	*-*	*Si, Fe*	*Al, K*	*Ca, (Cl), Na*	*Mg, (P)*	*Mixture of iron ox and aluminosil*
9	*AT9*	*3*	*-*	*W*	*-*	*-*	*Si, Fe*	*Al, Ca*	*K, (Cl), Na*	*Mg, (P), Ti*	*Mixture of iron ox and aluminosil*
12	AT12	1	Subcirc	W	1-2	1-2	**Fe**	(Si, Al, Ca)	(Cl, K)	Mn, (Mg, P, Ti)	Iron ox (+ aluminosil)
12	AT12	1	Plat	G	2	1	(Fe), Si	Al, (Cl), Ca	K, Mg	P, Ti	Clay min (+ iron ox)
12	AT12	1	Subcirc	G	23	19	(Fe), Si, Ca	(Cl), Al	K	P, Ti, Mg, Na	Ca-rich feldspar (+ iron ox)
12	AT12	1	Plat	G	50	26	Si, Al	(Fe), K	-	(Cl), Ti, Ca, Mg, Na	K-rich mica (+ iron ox)
12	AT12	1	Irreg	G	8	8	Fe, Si	Al, Mg, K	Cl, Ca	Ti, P	Undet
12	*AT12*	*1*	*-*	*G*	*-*	*-*	***Fe***, *Si*	*Cl, Al, Ca*	*K*	*Ti, P, Mg*	*Mixture of iron ox and aluminosil*
13	AT13	3	Aggl	W	Submicr	Submicr	Fe, Si	Al, Ce, La, Nd	Na	K, Ca, (P), Mg, Cl, Ti, (S), Mn	Mixture of iron ox and clay min
13	AT13	3	Tab	LG	113-202	30-48	(Fe), Si	Na	Al, K	Ca, Ti, (Mn), Mg, Cl	Na-rich feldspar (+ iron ox + clay min)
13	*AT13*	*3*	*-*	*G*	*-*	*-*	*Fe, Si*	*Al*	*Na*	*Ca, K, (Cl), Mg, P*	*Mixture of iron ox and aluminosil*
15	AT15	4	Irreg	W	20-79	14-66	**Fe**, Ti	(Si, Al)	-	(Ca, Mg, K), Mn, (Na, Cl, P, Cr, S)	Undet ox (+ clay min)
15	AT15	2	Aggl	W	Submicr-micr	Submicr-micr	**Ba**, S	(Si)	(Fe, Al)	(Ca, K, Na, Mg)	Ba-rich sulphate (+ clay min)
15	*AT15*	*3*	*-*	*G*	*-*	*-*	*Si, Fe*	*Al, Ca*	*K, S*	*Na, Mg, (Cl), Ti, P*	*Mixture of iron ox and clay min*
22	AT22	5	Ang	W	20-64	6-20	**Fe**	(Si)	(Al)	Mn, (Ti, Ca, K, Cl, P, Mg, S)	Iron ox (+ aluminosil)
22	AT22	1	Irreg	W	36	14	**Fe**, Si	-	Al	Ti, K, Mg, (Cl), Na	Fe-rich silicate
22	AT22	6	Irreg/ang	LG	28-70	19-61	(Fe), Si, Al	-	-	Ti, Ca, (Cl), K, P, Mg, S	Undet aluminosil (+ iron ox)
22	*AT22*	*6*	*-*	*G*	*-*	*-*	*Fe, Si*	*Al*	*-*	*K, Ca, Ti, (Cl), Mg, P, Na*	*Mixture of iron ox and aluminosil*

Num: number; sam: sample; an: analyses; morph: morphology; cont: contrast; irreg: irregular; aggl: agglomerate; plat: platy; ang: angular; subcirc: subcircular; oct: octahedral; acic: acicular; tab: tabular; W: white; G: gray; LG: light gray; submicr: submicrometric; micr: micrometric; ox: oxide; min: minerals; aluminosil: aluminosilicates; undet: undetermined.

(*): BSE cont. refers to the contrast observed on backscattered electron (BSE) images.

(**): Weight percentages including C and O, normalised to 100 %. Elements in brackets play no role in the mineralogical composition of the analyzed items, elements in bold are present in a proportion equal or higher than 40 %.

(***) Text in italic indicates analyses conducted on areas of 4 µm2 instead than on points.

Table B. Results of μ-RS analyses.

Num	Sample	Num of an	Grain morph	Grain colour	Identified compounds*
1	AT1A	4	Elo, amo	B	gth + (ms + undet aluminosil)
1	AT1B	1	Aggl	R	hem
2	AT2A	2	Aggl	Y	gth
2	AT2A	1	Irreg	B	gth + (ms + undet Mn oxide)
2	AT2A	1	Aggl	R	hem
2	AT2B	3	Aggl	R	hem + (qz + undet Mn oxide + undet aluminosil)
3	AT3	4	Aggl	R + G	hem + (qz)
3	AT3	1	Aggl	B + W	qz + hem
4	AT4	1	Aggl	R	gp + hem + (qtz + undet aluminosil)
4	AT4	5	Aggl	R + Y	gth + hem + (undet aluminosil)
5	AT5	4	Aggl	R	hem + (qz + cal)
6	AT6A	1	Tab	B	hem + gth
6	AT6A	2	Aggl	R + W	hem + (gth)
7	AT7	1	Irreg	B	C
7	AT7	2	Aggl	R + G	hem + qz
7	AT7	1	Tab	B	hem
7	AT7	3	Subcirc	B	hem
7	AT7	2	Aggl	Y	lep, (gth)
7	AT7	1	Subcirc	Y	gth
7	AT7	1	Angular	W	qz + (hem)
7	AT7	1	Subcirc	B	qz
8	AT8	3	Aggl	B	hem + (gp)
8	AT8	1	Angular	B	qz + (gth)
8	AT8	1	Subcirc	B	qz + (gp)
9	AT9	4	Aggl	B	hem
9	AT9	2	Subcirc	B	hem + (gp)
9	AT9	1	Angular	W + R	qz + (hem)
9	AT9	1	Irreg	G	qz + (hem + gp + gth)
10	AT10	6	Aggl	R	hem + (cal + man + undet aluminosil)
10	AT10	1	Irreg	B	hem + (qz)
10	AT10	1	Subcirc	Y	gth
11	AT11	6	Aggl	R + B	hem + (an + gp + qz + undet aluminosil)
11	AT11	2	Angular	B	mag + (gp)
11	AT11	1	Elo, amo	W	qz + (hem + gp)
12	AT12	1	Irreg	W	ab + (hem)
12	AT12	3	Aggl	R	hem
12	AT12	1	Subcirc	G	qz + (hem + undet aluminosil)
13	AT13	3	Aggl	Y	gth + (ab + hem)
13	AT13	1	Angular	G	qz + (hem + gth + C)
14	AT14	1	Aggl	Y	gth + (hem + mnt)
14	AT14	1	Irreg	B	hem
14	AT14	3	Aggl	R	hem + (cal + gth + qz + undet aluminosil)
14	AT14	2	Angular	B + G	qz + (C + gth + hem)
15	AT15	1	Irreg	B	hem + (gth + dol)
15	AT15	2	Aggl	R	hem + (mnt + undet aluminosil)
15	AT15	1	Subcirc	Y	gth
15	AT15	3	Irreg	T	dol
16	AT16	2	Irreg	Y	gth + (gp + hem)
16	AT16	3	Aggl	R	hem
16	AT16	1	Subcirc	B	qz + (hem)

17	AT17	2	Aggl	R	hem
17	AT17	2	Subcirc	Y	gth
17	AT17	1	Aggl	Y	kln
17	AT17	1	Angular	G	qz + (gp)
17	AT17	2	Irreg	B	qz + (gp + hem)
17	AT17	1	Tab	W	qz + kln + (gp + hem)
18	AT18	3	Irreg	B	hem + (gp)
18	AT18	3	Aggl	R	hem + (gth)
18	AT18	1	Irreg	G	qz + (mag)
19	AT19	6	Aggl	R	hem + (aug)
19	AT19	1	Ang	G	qz + gth
22	AT22	1	Aggl	Y	gth + (mnt)
22	AT22	1	Aggl	R	hem + (qz)

Num: number; an: analyses; morph: morphology; elo: elongated; amo: amorphous; aggl: agglomerate; irreg: irregular; tab: tabular; subcirc: subcircular; ang: angular; B: black; R: red; Y: yellow; G: gray; W: white; T: translucent. Abbreviations of minerals are based on the nomenclature suggested by [121], except for lepidocrocite (lep), and manganite (man); C: carbon.

(*) Minerals in brackets reflect the composition of area located outside the analysed spot.
