# Supplementary material for: Middle Stone Age Ochre Processing and Behavioural Complexity in the Horn of Africa: Evidence from Porc-Epic Cave, Dire Dawa, Ethiopia
Source: PLoS One. 2016 Nov 2;11(11):e0164793. doi: 10.1371/journal.pone.0164793 (PMC5091854; doi:10.1371/journal.pone.0164793)
Supplement: S1 Text — SEM-EDS, μ-RS and XRD analyses. The objects' identification number is the same as presented in Figs 4–11, Tables 1–5, S1 and S2 Figs, S1 Table. (PDF) [file pone.0164793.s004.pdf]

Middle Stone Age Ochre Processing and Behavioural Complexity in the Horn of Africa: Evidence from Porc-Epic Cave, Dire Dawa, Ethiopia

Daniela Eugenia Rosso*, Africa Pitarch Martí, Francesco d'Errico

* Corresponding author
E-mail: d.rosso@pacea.u-bordeaux1.fr (DR)

S1 Texts. Results of residue analysis on ochre processing tools and ochre-stained artefacts from Porc-Epic Cave.

SEM-EDS, µ-RS and XRD analyses. The objects' identification number is the same as presented in Figs 4–11, Tables 1–5, S1 and S2 Figs, S1 Tables.

Text A. Results of residue analysis from ochre processing tool 1 2
Text B. Results of residue analysis from ochre processing tool 2 3
Text C. Results of residue analysis from ochre processing tool 3 5
Text D. Results of residue analysis from ochre processing tool 4 6
Text E. Results of residue analysis from ochre-stained artefact 5 7
Text F. Results of residue analysis from ochre processing tool 6 8
Text G. Results of residue analysis from ochre processing tool 7 9
Text H. Results of residue analysis from ochre processing tool 8 10
Text I. Results of residue analysis from ochre processing tool 9 11
Text J. Results of residue analysis from ochre processing tool 10 12
Text K. Results of residue analysis from ochre processing tool 11 13
Text L. Results of residue analysis from ochre processing tool 12 13
Text M. Results of residue analysis from ochre processing tool 13 15
Text N. Results of residue analysis from ochre processing tool 14 16
Text O. Results of residue analysis from ochre-stained artefact 15 16
Text P. Results of residue analysis from ochre processing tool 16 17
Text Q. Results of residue analysis from ochre processing tool 17 18
Text R. Results of residue analysis from ochre processing tool 18 19
Text S. Results of residue analysis from ochre processing tool 19 20
Text T. Results of residue analysis from ochre processing tool 22 20

Text A. Results of residue analysis from ochre processing tool 1

Microscopic description and results of SEM-EDS, μ-RS and XRD analyses of samples AT1A, AT1B and T1.

Sample description

The ochre residue, relatively abundant in the irregularities of the tool's surface, was easy to remove with a scalpel. When observed through optical microscopy (Fig 8a, b, Fig A in S1 Figs), all sampled residues appear composed of aggregates of mostly red and, to a lesser degree, yellow fine-grained powder. Large white translucent and black grains, which probably come from the minerals composing the tool, are also present in the residue.

Sample composition

SEM-EDS analysis (Fig 10a, b, Fig 11, Fig A in S1 Figs, Table A in S1 Tables) shows that sample AT1A is composed of two different types of residue. Residue from zone 1 has a loose appearance and shows the presence of aggregates including what we interpreted as iron oxides (Fe) with clay minerals (Si, Al, Ca, K, Mg, and traces of Ti and Na), and K-rich micas (Si, Al, K, Na; approximately 34 μm in length). Residue from zone 2 has a compact appearance, and is composed of sub-micrometric and large (approximately 39 μm in length) irregular grains of iron oxides (Fe, Mn) and submicrometric platy grains (Si, Al, K, Ca, with traces of Mg and Na) interpreted as clay minerals. Irregular particles of barium sulphates (Ba, S; approximately 24 μm in length), and carbonates (Ca, Mg; approximately 30 μm in length) are also sporadically detected.

Micro-RS analyses (Table 4, Table B in S2 Tables) identify in sample AT1A the presence of goethite (FeO(OH)), muscovite (potassic mica, $KAl_2(AlSi_3O_{10})(OH)_2$) and other undetermined aluminosilicates. Sample AT1B shows the presence of hematite (Fe_2O_3). This is consistent with the results obtained through SEM-EDS.

XRD analyses (Table 5, Fig A in S1 Figs) confirm the presence in sample T1 of hematite, clay minerals (probably from the kaolinite group, $Al_2Si_2O_5(OH)_4$), and calcite ($CaCO_3$), and identify plagioclase feldspars (probably anorthite, $CaAl_2Si_2O_8$) and quartz (SiO_2).

Interpretation

Microscopic, elemental and mineralogical analyses suggest that this tool was used to process ochre lumps rich in iron oxides, clay minerals, and micas. The relationship between these components seems to indicate that they were naturally associated before being processed.

Differences in grain size and texture between two zones of sample AT1A analysed with SEM-EDS indicate the possible presence of two different types of ferruginous clay rocks. Iron oxides from zone 1 differ from those of zone 2, the first being submicrometric and composed of Fe, and the second being composed of larger grains, and composed of Fe and Mn. Additionally, clay minerals from zone 1 contain Ti, which is absent in zone 2.

Feldspar grains are detected through mineralogical analysis, but they were not directly analysed through SEM-EDS. However, SEM images from zone 2 seem to show some embedded irregular grains that could be interpreted as feldspars, absent in zone 1. Coarse quartz grains do not seem well blended with ochre residues, which may indicate that they originate from the limestone composing the processing tool. The low presence of barium sulphates suggests that they originate from surrounding sediments or post-depositional processes. This is also the case of carbonates detected by SEM-EDS.

Text B. Results of residue analysis from ochre processing tool 2

Microscopic description and results of SEM-EDS and μ-RS analyses of samples AT2A and AT2B.

Sample description

Small accumulations of ochre residues were detected in irregularities of the tool surface, making easy their extraction. Samples AT2A (Fig 8c, Fig B in S1 Figs) and AT2B (Fig 8d, Fig B in S1 Figs) are different macroscopically and microscopically. The former is yellow, the latter red, with very few yellow grains. In both cases, the ochre powder is fine-grained, and coarse translucent grains are present.

Sample composition

SEM-EDS analysis indicates that sample AT2A (Fig 10c, Fig 11, Fig B in S1 Figs, Table A in S1 Tables) contains compact agglomerates of submicrometric acicular grains of iron oxides (Fe, with traces of Ti), associated with platy submicrometric grains matching clay minerals (Si, Al, Ca, K, with traces of Mg, Ti, Na). Irregular Ca-rich grains, probably corresponding to plagioclase feldspars (Si, Al, Ca, K, Na; approximately 62 μm in length), and a few angular silicate grains (Si; approximately 42 μm in length) are also recorded.

Sample AT2B (Fig 10d, Fig 11, Fig B in S1 Figs, Table A in S1Tables) contains less compact agglomerates of fine submicrometric grains of iron oxides (Fe, Ti) associated with submicrometric platy grains corresponding to clay minerals (Si, Al, Ca, K, and traces of Mg, Na). Several large, angular silicate grains (approximately 42–156 μm) are present among the iron oxides.

Micro-RS analyses (Table 4, Table B in S1 Tables) show the presence in sample AT2A of goethite, and hematite. Manganese-rich minerals, muscovite and other undetermined aluminosilicates are also detected. In sample AT2B, hematite, quartz, undetermined aluminosilicates and manganese-rich minerals are recorded.

Interpretation

Microscopic, elemental and mineralogical analysis suggests that this tool was used to process two different types of ochre. The first type is a rock rich in fine-grained goethite (sample AT2A), and the second (sample AT2B) is almost exclusively composed of a fine-grained hematite.

In both cases, the blended appearance of iron oxides and clay minerals seems to indicate that they were naturally associated in origin. Both residues can be interpreted as ferruginous clay rocks.

Large quartz grains, identified in both samples, do not seem closely associated with the ochre powder. They seem more likely to be part of the minerals composing the schist tool. This is also the case with muscovite, undetermined manganese-rich minerals, and Ca-rich feldspars.

Text C. Results of residue analysis from ochre processing tool 3

Microscopic description and results of SEM-EDS, μ-RS and XRD analyses of samples AT3 and T3.

Sample description

Ochre residues on this tool are abundant, and were easy to remove. Samples AT3 and T3 are composed of fine red ochre powder, and translucent coarse grains (Fig 8e, Fig C in S1 Figs).

Sample composition

SEM-EDS analysis of sample AT3 (Fig 10e, Fig 11, Fig C in S1 Figs, Table A in S1 Tables) shows the presence of coarse silicate grains (Si, approximately 522–807 μm in length) cemented with iron oxides (Fe). Small subcircular grains probably corresponding to iron oxides, silicate grains (Si; approximately 5–10 μm in length), and submicrometric platy grains interpreted as clay minerals (Si, Al, Ca, K, Mg, and traces of Ti) are also identified.

Micro-RS analyses (Table 4, Table B in S1 Tables) identify hematite and quartz in sample AT3, which is consistent with the presence of iron oxides and silicates detected by SEM-EDS.

The presence of hematite and quartz is confirmed by XRD (Table 5, Fig C in S1 Figs) on sample T3. Clay minerals from the kaolinite-serpentine group (consistent with results of SEM-EDS analyses), and bernalite ($Fe(OH)_3$, – consistent with the presence of iron oxides– are also detected.

Interpretation

Results suggest the presence of two types of mineral associations. The first, characterized by large quartz grains cemented by iron oxides, probably originate from the ferruginous sandstone composing the tool. The second, made of fine-grained iron oxides associated with clay minerals such as kaolinite, correspond to a ferruginous clay rock processed on the tool. The small size and clean edges of quartz grains found in the residues, which differentiate them from those originating from the tool, indicate that they may have been added on purpose.

Text D. Results of residue analysis from ochre processing tool 4

Microscopic description and results of SEM-EDS and μ-RS analyses of sample AT4.

Sample description

Ochre residues were identified in small accumulations on the whole surface of the object, making the sample extraction relatively easy. Through optical microscopy (Fig 8f, Fig D in S1 Figs), sample AT4 shows the presence of a fine red powder, a few yellow grains, and translucent and black subcircular grains.

Sample composition

SEM-EDS analyses on sample AT4 (Fig 10f, Fig 11, Fig D in S1 Figs, Table A in S1 Tables) show the presence of octahedral grains (Fe, Ti; approximately 58–63 μm in length), agglomerates of submicrometric grains of iron oxides (Fe), clay minerals (Si, Al, Ca, with traces of K, Mg, Na), and coarse grains (Si) interpreted as silicates.

Micro-RS analyses on the same sample (Table 4, Table B in S1 Tables) show the presence of goethite, hematite, and quartz, which is consistent with the presence of iron oxides and silicates detected by SEM-EDS. Gypsum ($CaSO_4 \cdot 2H_2O$) is also detected.

Interpretation

Microscopic, elemental and mineralogical analyses suggest that this ochre processing tool was used to process two types of ochre: one rich in Fe/Ti oxides, and another type rich in submicrometric hematite and goethite grains associated with clay minerals and a few quartz grains. The blended appearance of submicrometric iron oxides and clay minerals seems to indicate that this second type of ochre was a ferruginous clay rock. Quartz does not appear to originate from the limestone of the tool, that shows a compact appearance, and is more likely to come from the processed ochre fragment, or from an additive mixed to the ochre powder.

Text E. Results of residue analysis from ochre-stained artefact 5

Microscopic description and results of SEM-EDS, μ-RS and XRD analyses of samples AT5 and T5.

Sample description

Ochre residues were abundant on this artefact but they were not easy to extract, as they appeared to be absorbed in the limestone, rather than disposed in accumulations. Through optical microscopy (Fig 8g, Fig E in S1 Figs), sample AT5 and sample T5 seem mostly composed of a light brown fine-grained powder, associated with an even finer bright red powder. Light brown and light gray coarse grains can be observed. A few black grains were also identified on sample T5.

Sample composition

When analysed through SEM-EDS (Fig 10g, Fig 11, Fig E in S1 Fig, Table A in S1 Tables), sample AT5 shows the presence of two ochre types. The first includes iron oxides in the form of small irregular grains (Fe; approximately 1–2 μm in length) embedded in a clay matrix (Si, Al, K, Ca, with traces of Mg, Na and Ti), and the second is composed of large irregular grains (Fe; approximately 17–38 μm). Larger irregular grains (Si, Al, Na K; approximately 24 μm in length) probably corresponding to feldspars, and irregular grains (Si; approximately 30 μm in length) interpreted as silicates are also observed.

Micro-RS analyses on the same sample (Table 4, Table B in S1 Tables) show the presence of hematite, quartz, and calcite.

XRD analyses on sample T5 (Table 5, Fig E in S1 Figs) confirm the presence of hematite and quartz. Manganese oxides, which were not detected through SEM-EDS or μ-RS, were also identified (ramsdellite, MnO_2). Analyses show the presence of other aluminosilicates, such as laumontite ($Ca(AlSi_2O_6)_2 \cdot 4H_2O$). Montmorillonite, $(Na,Ca)_{0,3}(Al,Mg)_2Si_4O_{10}(OH)_2 \cdot nH_2O$, and alkali feldspars, possibly sanidine, $KAlSi_3O_8$, are detected, which is consistent with results from SEM-EDS analyses. Gypsum was also found.

Interpretation

Microscopic, elemental and mineralogical analyses suggest that this tool was used to process two types of ochre, among which at least one was a ferruginous clay rock. It is probable that a few grains originating from the limestone composing the artefact were included in the sample. The identification of calcite and laumontite supports this view. The presence of quartz, sanidine and ramsdellite could come either from the minerals composing the artefact or from the processed ochre. Gypsum may have grown post-depositionnaly.

Text F. Results of residue analysis from ochre processing tool 6

Microscopic description and results of SEM-EDS, μ-RS and XRD analyses of samples AT6A, AT6B and T6.

Sample description

Samples from OPT 6 were easily removed, as small ochre accumulations were present in the irregularities of the surface. The three sampled residues are composed of agglomerates of fine red and yellow grains associated with black, white, and brown grains of different coarseness and shape (Fig 8h, i, Fig F in S1 Figs).

Sample composition

SEM-EDS analyses carried out on sample AT6B (Fig 10h, Fig 11, Fig F in S1 Figs, Table A in S1 Tables) show the presence of large grains containing thin platelets rich in Fe and Ti (approximately 55–134 μm in length), coated by agglomerates of submicrometric grains interpreted as clay minerals (Si, Al, Mg, K), associated with angular, coarse grains (Si, Ca, Mg, Fe, Al, Ti, with traces of Na; approximately 194 μm in length) probably corresponding to silicates.

Micro-RS analyses on sample AT6A (Table 4, Table B in S1 Tables) show the presence of hematite and goethite.

XRD analyses on sample T6 (Table 5, Fig F in S1 Figs) confirm the presence hematite and detected plagioclase feldspars, possibly albite ($NaAlSi_3O_8$) and anorthite. The diffractogram also highlights the presence of

minerals from the smectite group, possibly saponite, $Ca_{0.25}(Mg,Fe)_3((Si,Al)_4O_{10})(OH)_2 \cdot n(H_2O)$, and halloysite, $Al_2Si_2O_5(OH)_4$, which is consistent with the identification of clay minerals through SEM-EDS. Clinochlore, $(Mg,Fe^{2+})_5Al(AlSi_3O_{10})(OH)_8$, augite, $(Ca,Na)(Mg,Fe,Al,Ti)(Si,Al)_2O_6$, and quartz are also identified.

Interpretation

Microscopic, elemental and mineralogical analyses suggest that OPT 6 was used to process ochre composed of Fe-Ti oxides. The way in which clay minerals coat the Fe-Ti particles may indicate that they do not come from the same rock, and that they are part of additives, intentionally mixed during processing.

Albite, anorthite, clinochlore, augite and quartz, common in metamorphic rocks, are likely to come from the quartzite composing the processing tool.

Text G. Results of residue analysis from ochre processing tool 7

Microscopic description and results of SEM-EDS μ-RS and XRD analyses of samples AT7 and T7.

Sample description

All samples were very easily extracted, as they were disposed in small accumulations of residue on the surface of the tool. They are mostly composed of fine red grains and very few yellow agglomerates (Fig 8j, Fig G in S1 Figs). Translucent white subcircular grains can also be observed microscopically.

Sample composition

SEM-EDS analyses (Fig 10i, Fig 11, Fig G in S1 Figs, Table A in S1 Tables) show that sample AT7 contains agglomerates formed by platelets (approximately 2–4 μm in length) and larger irregular grains (approximately 20–29 μm in length), both interpreted as iron oxides (Fe). Other coarser, irregular grains are observed, and interpreted as Ca/Na-rich feldspars (Si, Al, Ca, Na; approximately 56 μm in length) and silicates (Si; approximately 326 μm in length).

Micro-RS on sample AT7 (Table 4, Table B in S1 Tables) and XRD analyses on sample T7 (Table 5, Fig G in S1 Figs) detected the presence of hematite, goethite, lepidocrocite ($Fe^{3+}O(OH)$), and quartz, which is consistent with SEM-EDS results. Carbon was also detected by μ-RS analysis.

Interpretation

Microscopic, elemental and mineralogical analysis suggest that OPT 7 was used to process an earthy hematite. Larger iron oxide grains, feldspars and quartz grains appear unrelated to the earthy hematite, and probably derive from the processing tool.

Text H. Results of residue analysis from ochre processing tool 8

Microscopic description and results of μ-RS and XRD analyses of samples AT8 and T8.

Sample description

Samples AT8 and T8 were easy to extract, as ochre residues were relatively abundant between the quartz grains composing the sandstone of which the processing tool is made. When observed through optical microscopy (Fig 8k, Fig H in S1 Figs), samples from this tool appear composed of a fine grained red powder, with a few yellow agglomerates. Rounded translucent grains are also part of the residues.

Sample composition

Micro-RS analyses on sample AT8 (Table 4, Table B in S1 Tables) show the presence of hematite, goethite, quartz and gypsum. Among these, only hematite and quartz are identified through XRD in sample T8 (Table 5, Fig H in S1 Figs), in addition to another iron oxide (maghemite, Fe_2O_3), as well as clay minerals (kaolinite) and anatase (TiO_2).

Interpretation

Mineralogical analyses confirm that residues from this tool contain hematite, goethite and kaolinite. This can either indicate that they come from a

ferruginous clay rock, or that clay minerals were used as additives. The presence of quartz is likely to come from the minerals composing the sandstone tool. It is less likely that quartz grains were used as additives, or that they were part of a ferruginous sandstone processed on the tool.

Text I. Results of residue analysis from ochre processing tool 9

Microscopic description and results of SEM-EDS, μ-RS and XRD analyses of samples AT9 and T9.

Sample description

Samples AT9 and T9 were difficult to extract, as the surface of the OPT was covered by a patina. Both samples show the presence of coarse, rounded, translucent and brownish grains, surrounded by small agglomerates of fine red grains (Fig 8l, Fig I in S1 Figs). A few yellow grains are recorded in sample AT9.

Sample composition

SEM-EDS analyses on sample AT9 (Fig 10j, Fig 11, Fig I in S1 Figs, Table A in S1 Tables) show the presence of compact agglomerates composed of micrometric platelets (Fe, 5–10 μm in length) and irregular submicrometric iron-rich grains (Fe). Both are interpreted as iron oxides. The second type is associated with agglomerates of clay minerals (Si, Al, Ca, and traces of, K, Na Mg and Ti), irregular grains possibly corresponding to K-rich feldspars (Si, K, Al; approximately 27–194 μm in length), and large grains (Si; 131–153 μm in length) interpreted as silicates.

Micro-RS analyses on the same sample (Table 4, Table B in S1 Tables) show the presence of hematite, goethite and quartz, which is consistent with the presence of iron oxides and silicates detected through SEM-EDS. Gypsum is also found.

XRD analyses on sample T9 (Table 5, Fig I in S1 Figs) confirm the presence of hematite and quartz. Plagioclase feldspars, possibly albite and anorthite, and alkali feldspars (possibly microcline, $KAlSi_3O_8$) are detected. The presence of clay minerals from the smectite group, is also confirmed by the identification of montmorillonite.

Interpretation

Samples from this tool include compact agglomerates of iron-rich platelets interpreted as residues originating from the processing of earthy hematite. Adjacent to these agglomerates, but not blended to them, are the plagioclase and alkali feldspars, quartz grains, clay minerals and other iron oxide particles (unrelated to those characterised as earthy hematite). They are interpreted as part of the minerals composing the granite of which is made the tool. Gypsum probably originates from the surrounding sediments.

Text J. Results of residue analysis from ochre processing tool 10

Microscopic description and results of µ-RS and XRD analyses of samples AT10 and T10.

Sample description

Samples AT10 and T10 were difficult to extract, as the residue was diffuse and there were no ochre accumulations in the irregularities of the surface. Samples are mostly composed of a fine red and yellow powder, associated with translucent grains (Fig 8m, Fig J in S1 Figs).

Sample composition

Micro-RS analyses on sample AT10 (Table 4, Table B in S1 Tables) show the presence of hematite, goethite, manganite (MnO(OH)), calcite, quartz and undertermined aluminosilicates.

Hematite, calcite, quartz, sanidine and laumontite are identified through XRD (Table 5, Fig J in S1 Figs).

Interpretation

Hematite and goethite-rich rocks were processed on this tool. Aluminosilicates (sanidine and laumontite), silica (quartz) and carbonates (calcite) may originate from the quartzite composing the tool. However, the manganite cannot come from the tool, as its black colour would have been visible on the tool surface, which is white. It is more likely that the manganite

derives from the processed ochre, an additive intentionally mixed to the ochre powder, or the cave sediment.

Text K. Results of residue analysis from ochre processing tool 11

Microscopic description and results of μ-RS analyses of sample AT11.

Sample description

The ochre residue, relatively abundant in the irregularities of the working surface of OPT 10, was easy to remove with the scalpel. When observed through optical microscopy (Fig 8n, Fig K in S1 Fig), sample AT11 is composed of a fine-grained red powder. Very few angular black and white grains are also observed.

Sample composition

Micro-RS analyses (Table 4, Table B in S1 Tables) show that sample AT11 contains hematite, magnetite ($FeO \cdot Fe_2O_3$), feldspars (anorthite), quartz, gypsum, and undetermined aluminosilicates.

Interpretation

Residues from this tool include a hematite-rich component. Other identified minerals (anorthite, quartz, and magnetite) probably come from the tool, which is made of a fine grained, mafic (dark-colored) igneous rock. The presence of gypsum is probably due to post-depositional processes.

Text L. Results of residue analysis from ochre processing tool 12

Microscopic description and results of SEM-EDS, μ-RS and XRD analyses of samples AT12 and T12.

Sample description

Sample AT12 was difficult to extract, as ochre residues were diffuse and there were no ochre accumulations on the irregularities of the surface. Microscopically, samples AT12 and T12 are composed of a fine-grained red

powder (Fig 8o, Fig A in S2 Figs). Coarse angular translucent grains and a few black particles are also identified.

Sample composition

SEM-EDS analysis (Fig 10k, Fig 11, Fig A in S2 Figs, Table A in S1 Tables) shows that sample AT12 is composed of 1–2 μm long subcircular grains (Fe, with traces of Mn), probably corresponding to iron oxides, consistently associated with micrometric platelets interpreted as clay minerals (Si, Al, Ca, K, Mg), as well as coarser grains including platelets identified as micas (Si, Al, K, Na), and subcircular grains identified as plagioclase feldspars (Si, Ca, Al, K).

Results of μ-RS analysis in the same sample (Table 4, Table B in S1 Tables) are consistent with SEM-EDS results: hematite, albite and undetermined aluminosilicates are detected. Quartz is also found.

XRD analyses on sample T12 (Table 5, Fig A in S2 Figs) only allow us to confirm the presence of hematite and quartz. Goethite is also detected, even though yellow residues cannot be observed microscopically. However, SEM identifies a few acicular particles that are consistent with this type of iron oxide.

Interpretation

Microscopic, elemental and mineralogical analyses of sample AT12 suggest that OPT 12 was used to process one or more ochre fragments rich in hematite, and, to a lesser degree, goethite. The embedded appearance of clay minerals, feldspars and micas with respect to iron oxides seems to indicate that they come from the same ochre. Given the difference in granulometry and unblended appearance of quartz grains, we interpret them as originating from the processing tool.

Text M. Results of residue analysis from ochre processing tool 13

Microscopic description and results of SEM-EDS and μ-RS analyses of sample AT13.

Sample description

Sample AT13 was difficult to remove, as ochre residues on this piece are small and spread all over the surface. Under optical microscopy, sample AT13 reveals the presence of small agglomerates of fine-grained brown-red and yellow powder (Fig 8p, Fig B in S2 Figs). A few angular translucent grains are also observed.

Sample composition

SEM-EDS analyses (Fig 10l, Fig 11, Fig B in S2 Figs, Table A in S1 Tables) identify agglomerates of submicrometric acicular grains of iron oxides (Fe), associated with submicrometric grains interpreted as clay minerals (Si, Al, Na, with low contents of K, Ca, Mg, and Ti). Some areas show the presence of rare earth elements (La, Ce, Nd). Large tabular grains (Si, Na, Al, K, 113–202 μm in length) are also identified, and interpreted as Na-rich feldspars.

Micro-RS analyses (Table 4, Table B in S1 Tables) confirm the presence, in the same sample, of iron oxides (hematite and goethite), and plagioclase feldspars (albite). Quartz and traces of carbon are also detected.

Interpretation

Microscopic, elemental and mineralogical analyses of sample AT13 show that iron oxides (hematite and goethite) and clay minerals are consistently associated. It is therefore likely that a ferruginous clay rock was processed on this tool.

The granulometry and morphology of feldspars indicate that they do not originate from the same rock as the iron oxides and clay minerals, and suggest that these particles were not ground. They can be part of the minerals composing the tool, which is made of a granitoid rock. Quartz is probably part of the minerals composing the tool. La, Ce, Nd also probably come from the processing tool since granitoid rock may include rare earth elements.

Text N. Results of residue analysis from ochre processing tool 14

Microscopic description and results of μ-RS analyses of sample AT14.

Sample description

Residues from this tool were difficult to sample, as ochre residues were diffuse, and there were no ochre accumulations in the irregularities of the surface. Through optical microscopy (Fig 8q, Fig C in S2 Figs), sample AT14 appears composed of agglomerates of fine-grained red ochre powder, associated with a few agglomerates of yellow fine grains, fine irregular black grains and coarse angular translucent grains.

Sample composition

Micro-RS analyses (Table 4, Table B in S1 Tables) show that sample AT14 contains goethite, hematite, quartz, montmorillonite, calcite, carbon and undetermined aluminosilicates.

Interpretation

This tool was used to process ochre rich in hematite and goethite. The presence of montmorillonite could indicate that the processed ochre was a ferruginous clay rock. However, it could also indicate a use of clayish additives. Detected aluminosilicates and silicates could originate from the processed ochre, from the quartzite composing the tool, or be part of additives intentionally mixed to the ochre powder. Carbonates may originate from the quartzite, where they play the role of cementing material, or result from post-depositional processes.

Text O. Results of residue analysis from ochre-stained artefact 15

Microscopic description and results of SEM-EDS and μ-RS analyses of sample AT15.

Sample description

Sample AT15 was easy to extract. Under optical microscopy (Fig 8r, Fig D in S2 Figs), the sample is composed of agglomerates of a fine red powder

including fine yellow grains. A fine white powder is also detected, in addition to a few angular translucent grains.

Sample composition

SEM-EDS analyses (Fig 10m, Fig 11, Fig D in S2 Figs, Table A in S1 Tables) show that sample AT15 is composed of submicrometric grains of iron oxides (Fe, with traces of Ti) consistently associated with submicrometric grains interpreted as clay minerals (Si, Al, Ca, K, with traces of Na and Mg). Large particles rich in Fe and Ti, with traces of Mn (approximately 20–79 μm in length) are also detected, as well as fine-grained agglomerates submicrometric to micrometric interpreted as Ba-rich sulphates (Ba, S).

Micro-RS analyses (Table 4, Table B in S1 Tables) show the presence on the same sample of hematite, goethite, montmorillonite, and other undetermined aluminosilicates, which is consistent with SEM-EDS results. Dolomite ($CaMg(CO_3)_2$) was also detected.

Interpretation

Microscopic, elemental and mineralogical analyses of sample AT15 identify two types of ochre: one composed of iron oxides and clay minerals, the other of iron oxides rich in titanium. Grain size, morphology and composition indicate that these two ochres do not come from the same rock. The dolomite and Ba-rich sulphates are more likely part of the minerals composing the limestone of the artefact.

Text P. Results of residue analysis from ochre processing tool 16

Microscopic description and results of μ-RS analyses of sample AT16.

Sample description

Sample AT16 was easy to remove. The irregularities of the working surface of the OPT were rich in ochre residue. The sample is composed of a very fine-grained bright red powder, with a few yellow agglomerates (Fig 8s, Fig E in S2 Figs).

Sample composition

Micro-RS spectroscopy (Table 4, Table B in S1 Tables) identifies hematite, goethite, quartz, and gypsum.

Interpretation

The brightness of the red powder may indicate that a pure iron oxide was processed. Clay minerals and other aluminosilicates are not detected by μ-RS, but this does not mean that they are not present. Quartz grains probably originate from the tool, made of quartzite. The presence of gypsum is attributed to post-depositional processes.

Text Q. Results of residue analysis from ochre processing tool 17

Microscopic description and results of μ-RS and XRD analyses of sample AT17 and T17.

Sample description

Samples AT17 and T17 were easy to remove, as ochre residues are abundant on the surface of this tool. They are mostly composed of fine red and yellow agglomerates (Fig 8t, Fig F in S2 Figs). Large subcircular translucent grains coming from the friable sandstone composing the tool are also incorporated in the sampled residue.

Sample composition

Micro-RS analyses on sample AT17 (Table 4, Table B in S1 Tables) show that the sample is rich in hematite and goethite. The most present mineral in the sample is quartz, but clay minerals (kaolinite) and gypsum are also detected.

XRD analyses on sample T17 (Table 5, Fig F in S2 Figs) confirm the presence of hematite, quartz and kaolinite. Calcite and possibly alkali feldspars (orthoclase, $KAlSi_3O_8$) are also identified.

Interpretation

The detection of hematite, goethite and kaolinite in sample OPT 17 indicates that a ferruginous clay rock may have been processed on this tool. Kaolinite, however, could also be present in the sandstone composing the tool. Orthoclase and calcite could also come from the tool, the former originating from the matrix, and the latter from the cement. The presence of gypsum could be due to a post-depositional contamination.

Text R. Results of residue analysis from ochre processing tool 18

Microscopic description and results of μ-RS and XRD analyses of sample AT18 and T18.

Sample description

Samples AT18 and T18 were easy to extract, as ochre residues are abundant on OPT 18. Through optical microscopy (Fig 8u, Fig G in S2 Figs), samples are composed of agglomerates of a bright red, yellow and orange powder. A few white and dark irregular grains are also present in the residue.

Sample composition

Micro-RS analyses on sample AT18 (Table 4, Table B in S1 Tables) show the presence of hematite, goethite, magnetite, quartz and gypsum.

XRD analyses on sample T18 (Table 5, Fig G in S2 Figs) confirm the presence of hematite and quartz, and detect the presence of kaolinite.

Interpretation

Microscopic observations and mineralogical analyses show the presence of different types of iron oxides associated with clay minerals that could indicate that this tool was used to process a ferruginous clay rock. However, this tool is made of a coarse sandstone cemented with different types of iron oxides, which makes it difficult to determine which are part of the minerals composing the tool, and which are part of the processed ochre. Quartz particles probably originate from the sandstone of the tool, and the presence of gypsum is likely due to post-depositional processes.

Text S. Results of residue analysis from ochre processing tool 19

Microscopic description and results of μ-RS analyses of sample AT19.

Sample description

Ochre samples were relatively easy to extract as a small ochre accumulation was identified on the surface of the tool. Sample AT19 is characterized by the presence of a fine-grained red residue, with agglomerates of fine yellow grains (Fig 8v, Fig H in S2 Fig). A few irregular white and translucent grains can also be observed.

Sample composition

Micro-RS analyses on sample AT19 (Table 4, Table B in S1 Tables) show the presence of hematite, goethite, quartz and augite.

Interpretation

Microscopic and mineralogical analyses indicate that OPT 19 was used to process hematite and goethite-rich rocks. Clay minerals are not detected with μ-RS, but this is no proof of their absence in the residue.

Quartz and augite can derive from the granite composing the tool. The fact that these minerals are not blended with the ochre powder supports this hypothesis.

Text T. Results of residue analysis from ochre processing tool 22

Microscopic description and results of SEM-EDS and μ-RS analyses of sample AT22.

Sample description

Sample AT22 was not easy to remove, as residues were not abundant on this tool. Microscopically (Fig 8w, Fig K in S2 Fig), the residue appears composed of agglomerates of very fine red and yellow grains. A few white irregular grains are also present in the residue.

Sample composition

SEM-EDS analyses of sample AT22 (Fig 10n, Fig 11, Fig K in S2 Fig, Table A in S1 Tables) identify agglomerates of submicrometric iron-rich cubic grains, larger, angular grains of iron oxides (Fe with traces of Mn, approximately 20–64 μm in length), irregular iron-rich silicate grains (Fe, Si, Al, approximately 36 μm in length), and undetermined aluminosilicates (Si, Al, 28–70 μm in length).

Micro-RS analyses detect (Table 4, Table B in S1 Tables) goethite and hematite. Clay minerals, identified as montmorillonite and quartz are also identified.

Interpretation

Microscopic observations, elemental and mineralogical analysis identify iron oxides in the residue. However, the tool is made of a volcanic rock (andesite) that naturally contains iron oxides, generated by oxidation during cooling or subsequent meteorization of the lava. It is therefore difficult to determine whether the detected hematite and goethite originate from the processed ochre or from surface alteration. The detected montmorillonite may derive from the alteration of feldspars originally present in the andesite. Quartz may also come from the tool.
